# Supplementary material for: Molecular Simulations of Intact Anion Exchanger 1 Reveal Specific Domain and Lipid Interactions
Source: Biophys J. 2019 Aug 30;117(7):1364–79. doi: 10.1016/j.bpj.2019.08.029 (PMC6818359; doi:10.1016/j.bpj.2019.08.029)
Supplement: Document S1. Figs. S1–S12 and Tables S1–S3 [file mmc1.pdf]

Biophysical Journal, Volume *117*

Supplemental Information

Molecular Simulations of Intact Anion Exchanger 1 Reveal Specific Domain and Lipid Interactions

Dario De Vecchis, Reinhart A.F. Reithmeier, and Antreas C. Kalli

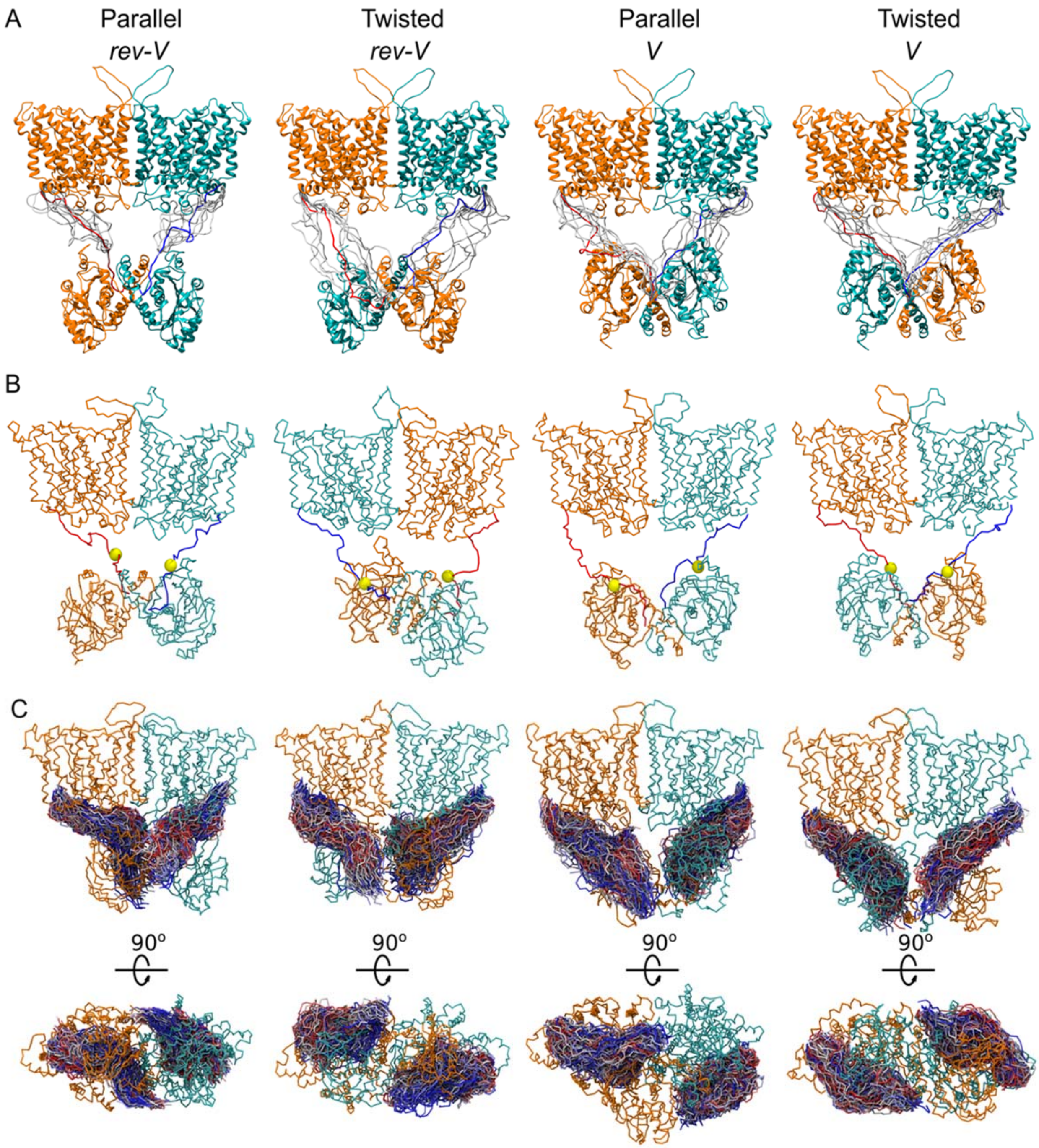

Figure S1. Conformational dynamics of the AE1 linker region. Related to Figures 1, 2 and Methods. (A) Structures are side views of all dimeric AE1 conformations after the modeling stage, showing the relative positions of the mdAE1, the cdAE1, and the linker region. The conformation of the linker of the structures that were used to initiate our simulations are shown in *red* and *blue*. All other conformations of the linker region that were generated during modelling are shown in *grey*. (B) The less sampled conformation for each conformer during the CG-MD simulations. The Lys360 residue is shown as *yellow* sphere. (C) The most sampled conformation for each conformer in the CG-MD simulations. The position of the linker region from all the centroids from all clusters are shown colored from *red* (more populated) to *blue* (less populated). In all panels, the two AE1 subunits are represented in *orange* and *cyan*.

Table S1. Summary of simulations with AE1 in native lipid mixture. Related to Methods.

Simulation	Composition Outer leaflet	Composition Inner leaflet	Duration
Coarse-grained:			
pa_rev-V_AE1-native	POPC:SM:POPE:CHOL (23:22:5:50)	POPC:SM:POPE:CHOL:POPS:PIP_2 (8:4:23:50:13:2)	5 × 5 µs
tw_rev-V_AE1-native	POPC:SM:POPE:CHOL (23:22:5:50)	POPC:SM:POPE:CHOL:POPS:PIP_2 (8:4:23:50:13:2)	5 × 5 µs
pa_V_AE1-native	POPC:SM:POPE:CHOL (23:22:5:50)	POPC:SM:POPE:CHOL:POPS:PIP_2 (8:4:23:50:13:2)	5 × 5 µs
tw_V_AE1-native	POPC:SM:POPE:CHOL (23:22:5:50)	POPC:SM:POPE:CHOL:POPS:PIP_2 (8:4:23:50:13:2)	5 × 5 µs
pa_rev-V_AE1-nolinker	POPC:SM:POPE:CHOL (23:22:5:50)	POPC:SM:POPE:CHOL:POPS:PIP_2 (8:4:23:50:13:2)	5 × 5 µs
tw_rev-V_AE1-nolinker	POPC:SM:POPE:CHOL (23:22:5:50)	POPC:SM:POPE:CHOL:POPS:PIP_2 (8:4:23:50:13:2)	5 × 5 µs
pa_V_AE1-nolinker	POPC:SM:POPE:CHOL (23:22:5:50)	POPC:SM:POPE:CHOL:POPS:PIP_2 (8:4:23:50:13:2)	5 × 5 µs
tw_V_AE1-nolinker	POPC:SM:POPE:CHOL (23:22:5:50)	POPC:SM:POPE:CHOL:POPS:PIP_2 (8:4:23:50:13:2)	5 × 5 µs
Atomistic:			
pa_rev-V_AE1_AT_native	POPC:SM:POPE:CHOL (23:22:5:50)	POPC:SM:POPE:CHOL:POPS:PIP_2 (8:4:23:50:13:2)	3 × 500 ns
tw_rev-V_AE1_AT_native	POPC:SM:POPE:CHOL (23:22:5:50)	POPC:SM:POPE:CHOL:POPS:PIP_2 (8:4:23:50:13:2)	3 × 500 ns
pa_V_AE1_AT_native	POPC:SM:POPE:CHOL (23:22:5:50)	POPC:SM:POPE:CHOL:POPS:PIP_2 (8:4:23:50:13:2)	3 × 500 ns
tw_V_AE1_AT_native	POPC:SM:POPE:CHOL (23:22:5:50)	POPC:SM:POPE:CHOL:POPS:PIP_2 (8:4:23:50:13:2)	3 × 500 ns

Lipid species are: 1-palmitoyl-2-oleyl-phosphtidylcholine (POPC), 1-palmitoyl-2-oleyl-phosphtidylethanolamine (POPE), 1-palmitoyl-2-oleyl-phosphtidylserine (POPS), phosphatidylinositol 4,5-bisphosphate (PIP_2), sphingomyelin (SM) and cholesterol (CHOL).

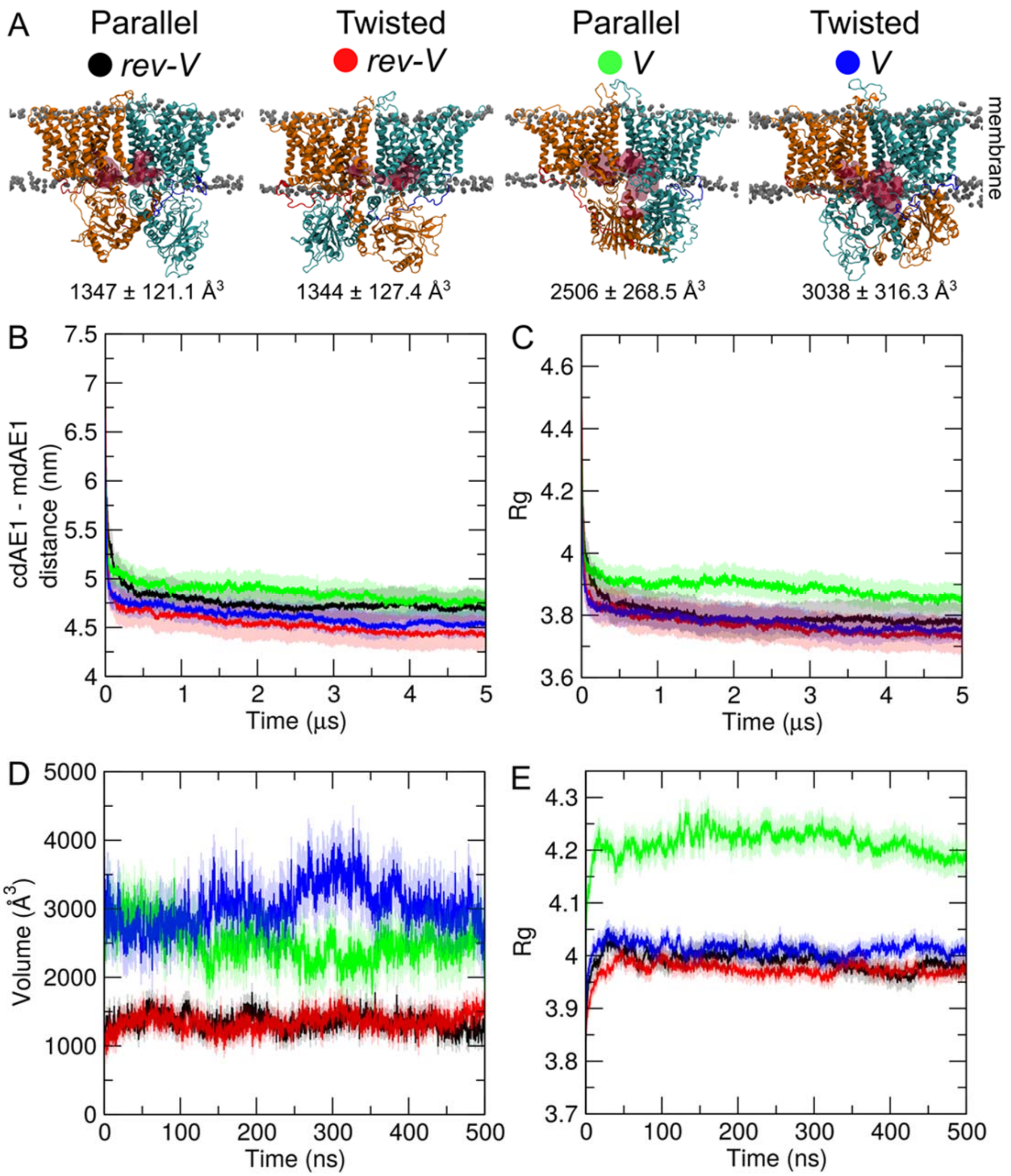

Figure S2. Distance and radius of gyration. Related to Figure 1. (A) Volume of the cavity between the cdAE1 and the mdAE1 dimers. The cavity is shown as a red surface in snapshots from our atomistic simulations. The average value ± standard deviation of the volume is also shown. The AE1 dimer subunits are shown in *orange* and *cyan* ribbon with the linker regions in *red* and *blue*. Phosphate groups from the lipid bilayer are shown as *grey* spheres. (B) Average distance between the cdAE1 and the mdAE1 center of mass during the CG-MD simulations. (C) Radius of gyration of AE1 during the CG-MD simulations. (D) Volume of the cavity shown in A (between the cdAE1 and the mdAE1). (E) Radius of gyration of the AE1 dimer in the atomistic simulations. In all plots the errors are shown and each timeline represents the average over the five repeat CG-MD simulations or the three repeats of the atomistic simulations.

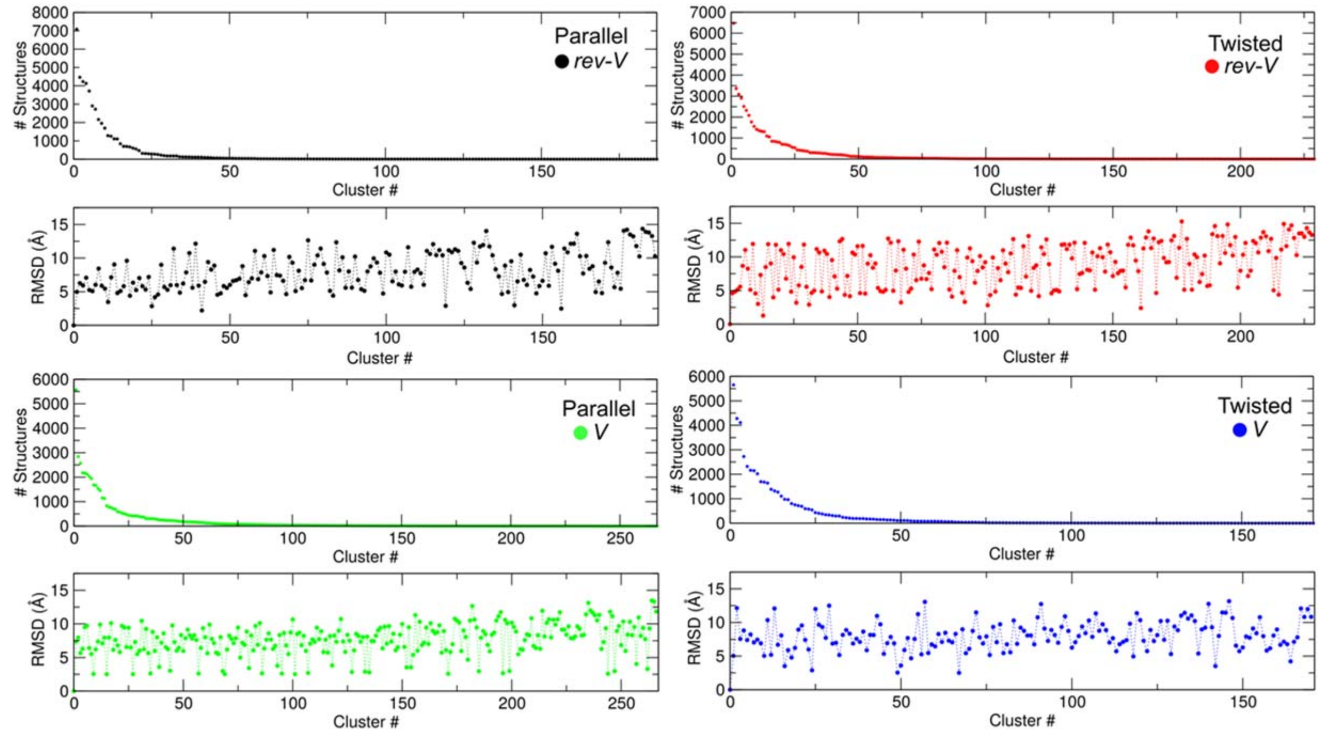

Figure S3. Details of the cluster analysis. Related to Figure 2. For each AE1 conformer (indicated with four different colors) two plots are shown. The plot at the top shows the total number of structures for each cluster. The plots at the bottom show the RMSD variation between the clusters. The structure of the first most populated cluster, has been used as a reference structure to calculate the RMSD of the protein in each cluster.

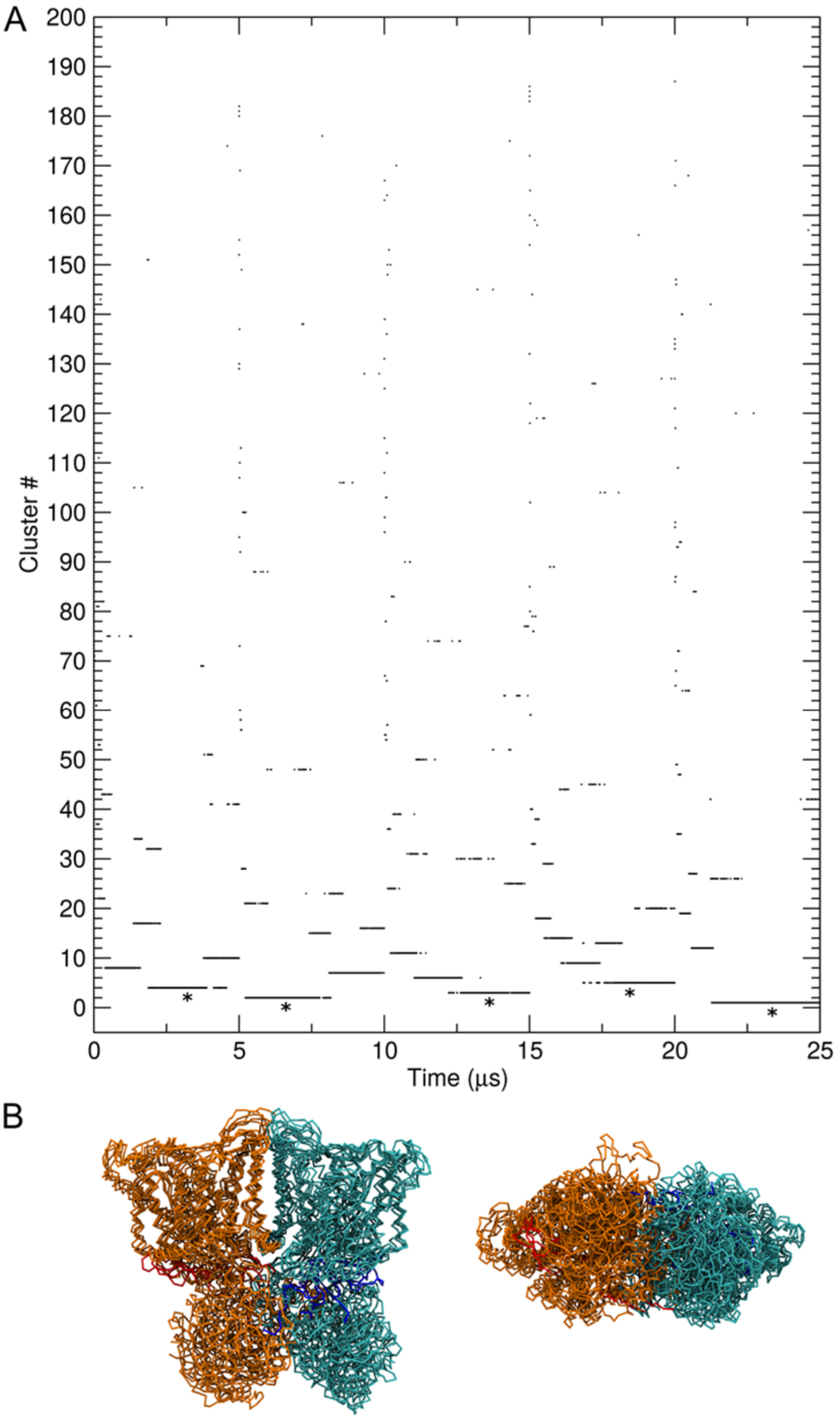

Figure S4. Cluster analysis for the *rev-V parallel* conformer. Related to Figure 2. (A) Occurrence of each cluster as a function of time over the total 25 µs of simulation. The asterisks indicate the first cluster for each repeat CG simulation (every 5 µs). (B) Structures of the centroid of the biggest cluster from each CG-MD repeat simulations (shown using an asterisks). The proteins have been aligned using the backbone atom of the mdAE1. The two AE1 chains are shown in *orange* and *cyan*, with the linker region in *red* and *blue*, respectively.

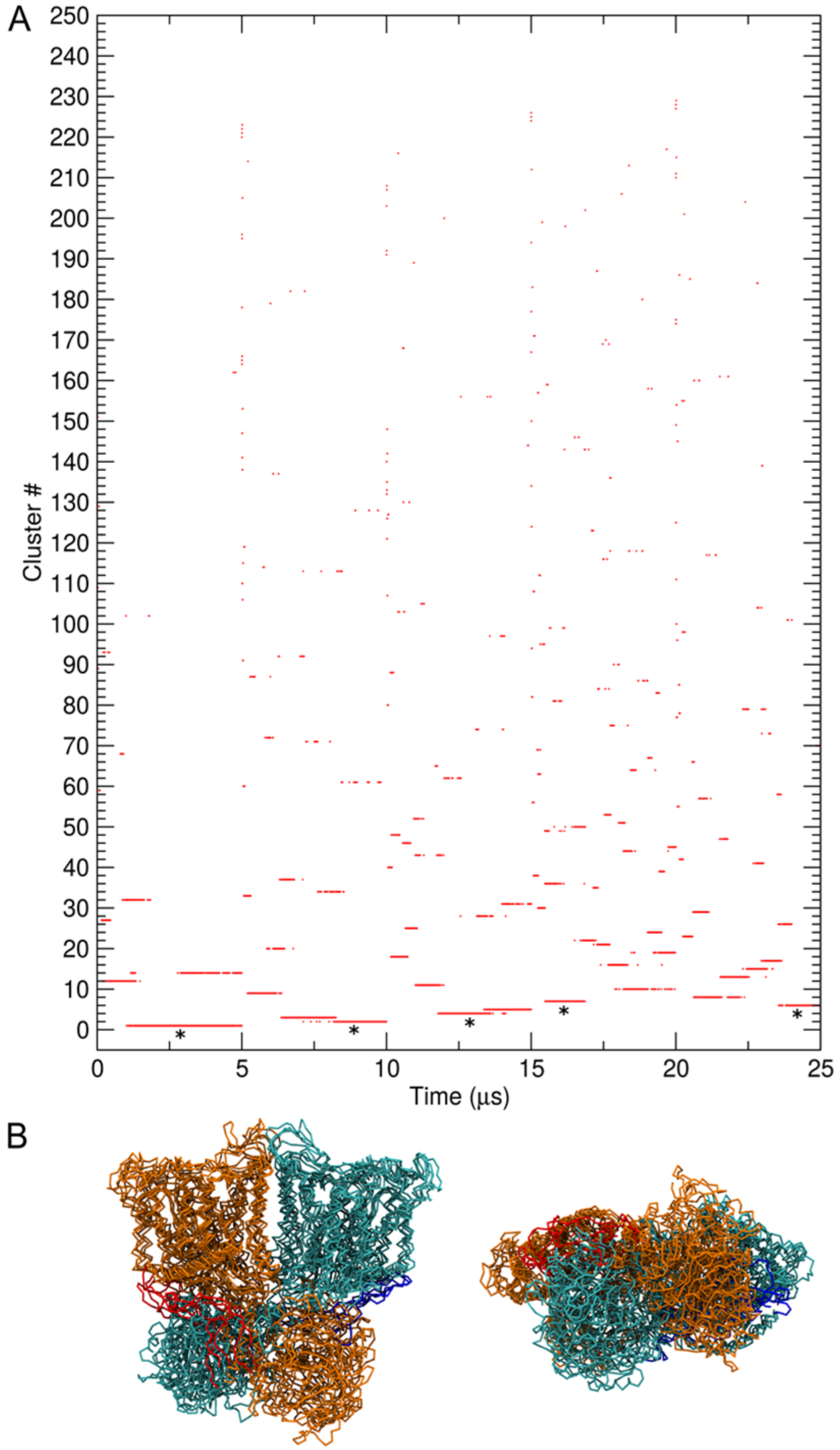

Figure S5. Cluster analysis for the *rev-V Twisted* conformer. Related to Figure 2. A) Occurrence of each cluster as a function of time over the total 25 µs of simulation. The asterisks indicate the first cluster for each repeat CG simulation (every 5 µs). (B) Structures of the centroid of the biggest cluster from each CG-MD repeat simulations (shown using an asterisks). The proteins have been aligned using the backbone atom of the mdAE1. The two AE1 chains are shown in *orange* and *cyan*, with the linker region in *red* and *blue*, respectively.

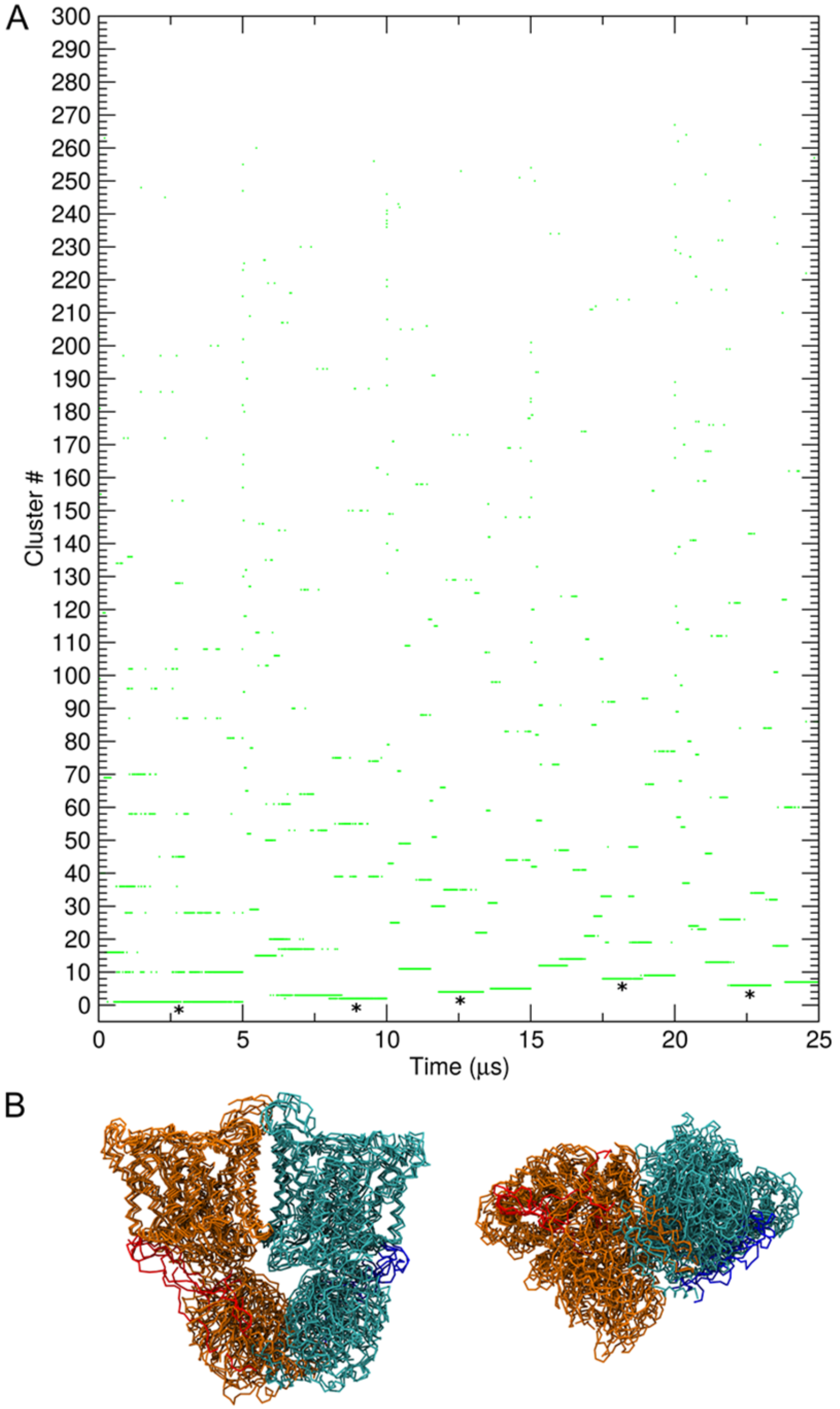

Figure S6. Cluster analysis for the *V Parallel* conformer. Related to Figure 2. A) Occurrence of each cluster as a function of time over the total 25 µs of simulation. The asterisks indicate the first cluster for each repeat CG simulation (every 5 µs). (B) Structures of the centroid of the biggest cluster from each CG-MD repeat simulations (shown using an asterisks). The proteins have been aligned using the backbone atom of the mdAE1. The two AE1 chains are shown in *orange* and *cyan*, with the linker region in *red* and *blue*, respectively.

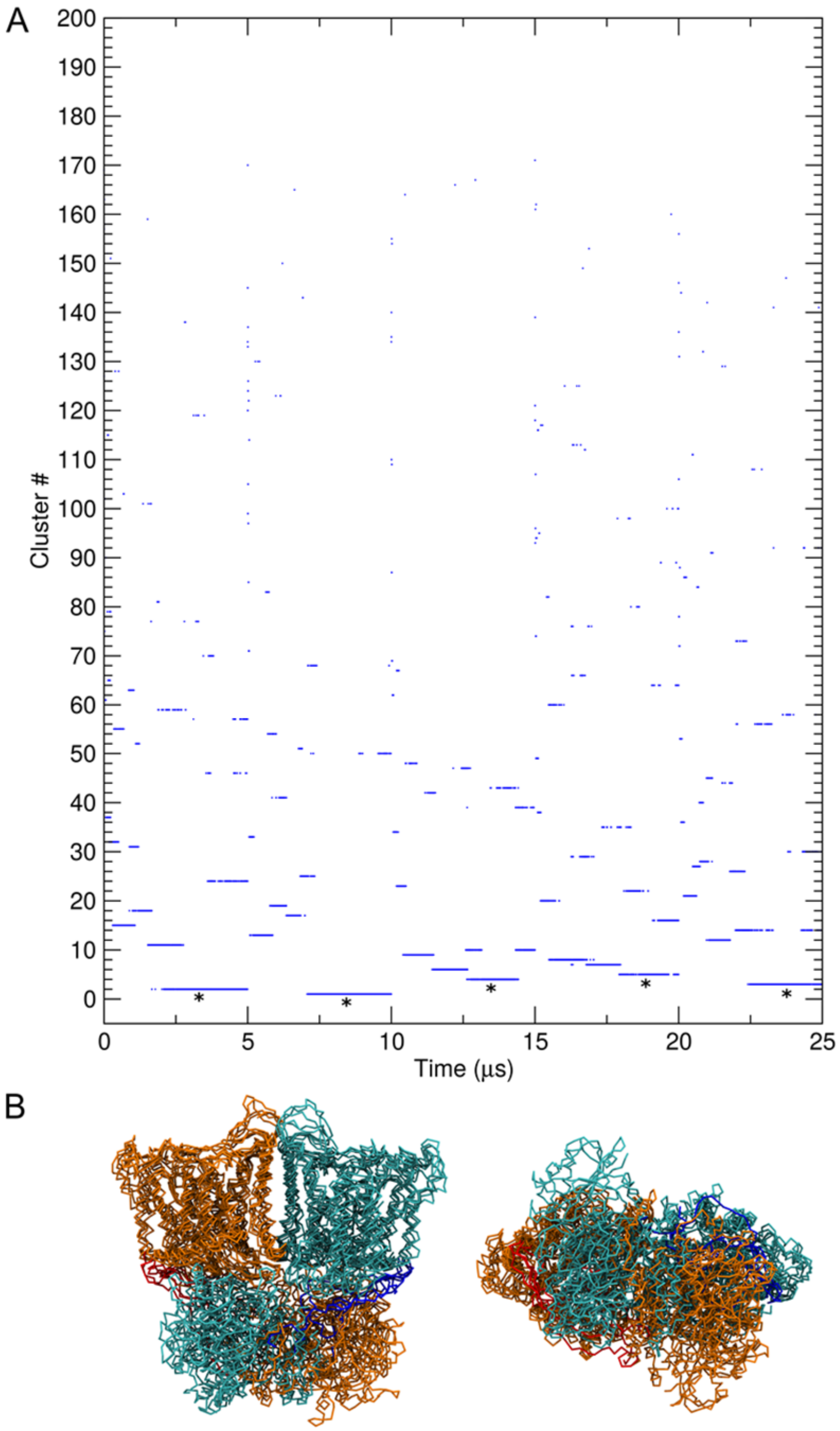

Figure S7. Cluster analysis for the *V Twisted* conformer. Related to Figure 2. A) Occurrence of each cluster as a function of time over the total 25 µs of simulation. The asterisks indicate the first cluster for each repeat CG simulation (every 5 µs). (B) Structures of the centroid of the biggest cluster from each CG-MD repeat simulations (shown using an asterisks). The proteins have been aligned using the backbone atom of the mdAE1. The two AE1 chains are shown in *orange* and *cyan*, with the linker region in *red* and *blue*, respectively.

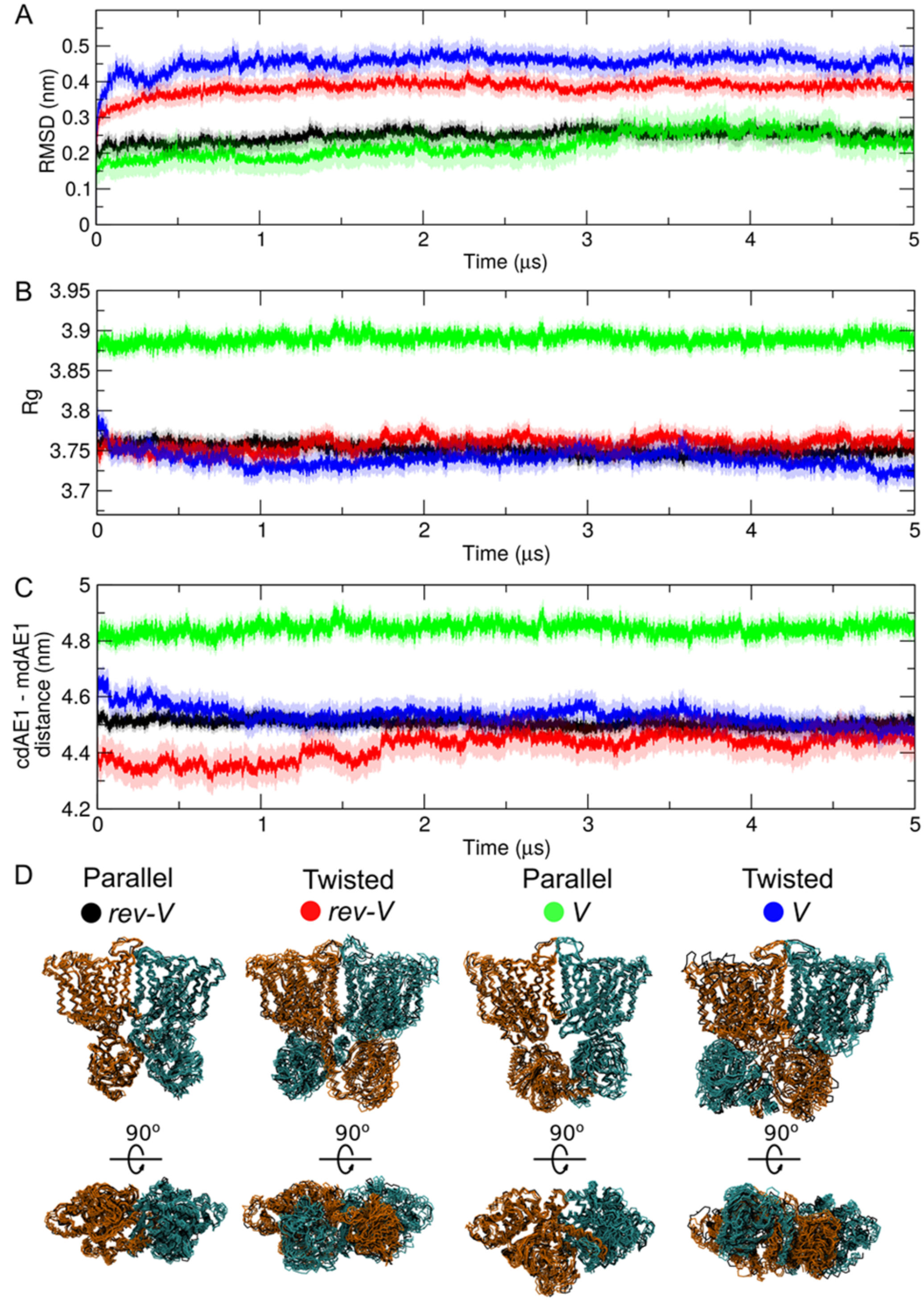

Figure S8 Molecular dynamics simulations without the linker region. Related to Figure 2. (A) RMSD and (B) radius of gyration, both calculated using the backbone particles of the protein. The color for each conformer is shown in D. (C) Distance between the center of mass of the cytoplasmic (cdAE1) and of the transmembrane (mdAE1) domain for each conformer. In all plots the errors are shown and each timeline represents the average over the five repeat CG simulations. (D) Alignment using the backbone particles of the last frame from each of the five repeat simulations and the first selected cluster from which simulations were initiated (in *black*). The two AE1 chains are shown in *orange* and *cyan*.

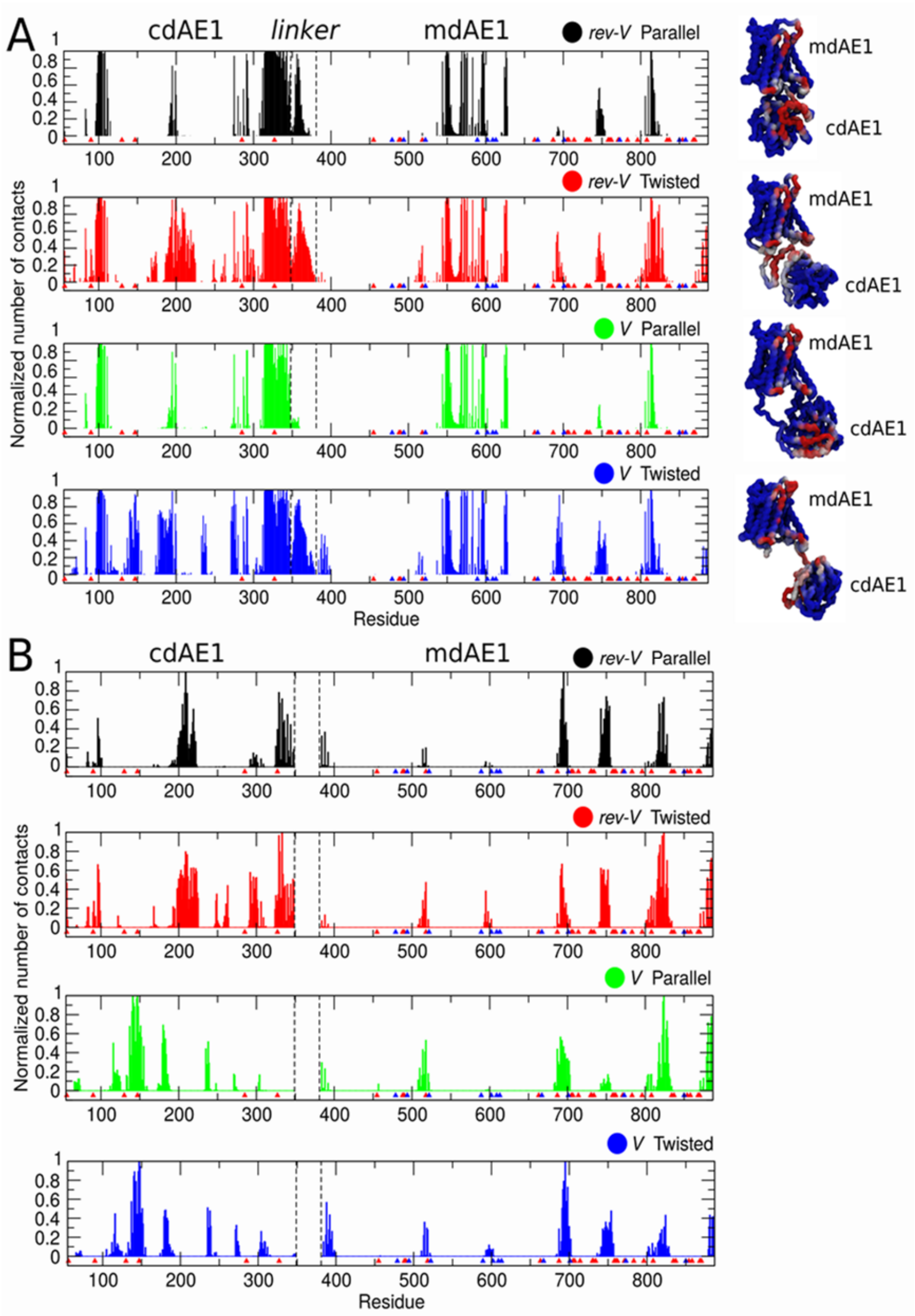

Figure S9. Interactions within the AE1 dimer. Related to Figure 4. Normalized average (over all repeat CG-MD simulations) number of contacts between the two AE1 monomers of the AE1 dimer (A) and between the cdAE1 and the mdAE1 domains (B). Note that for clarity the linker region was not included in the analysis in B and for this reason no contacts are shown between the dotted lines. The contact of the linker region with the mdAE1 and the cdAE1 are shown in the main text. The protein structures in A are snapshots from each conformer in which, the surface of the backbone beads is colored from *blue* (low number of contacts) to *red* (high number of contacts). The mdAE1 is positioned in the same orientation in all structures. In both analyses, the contacts of each AE1 chain were added together. Values close to 0 represents no/low number of contacts whereas 1 represents high number of contacts. A cutoff distance of 0.55 nm was used. Point mutations reported to cause spherocytosis (SPH) or distal renal tubular acidosis (AD-dRTA) are indicated as *red* and *blue* triangles, respectively.

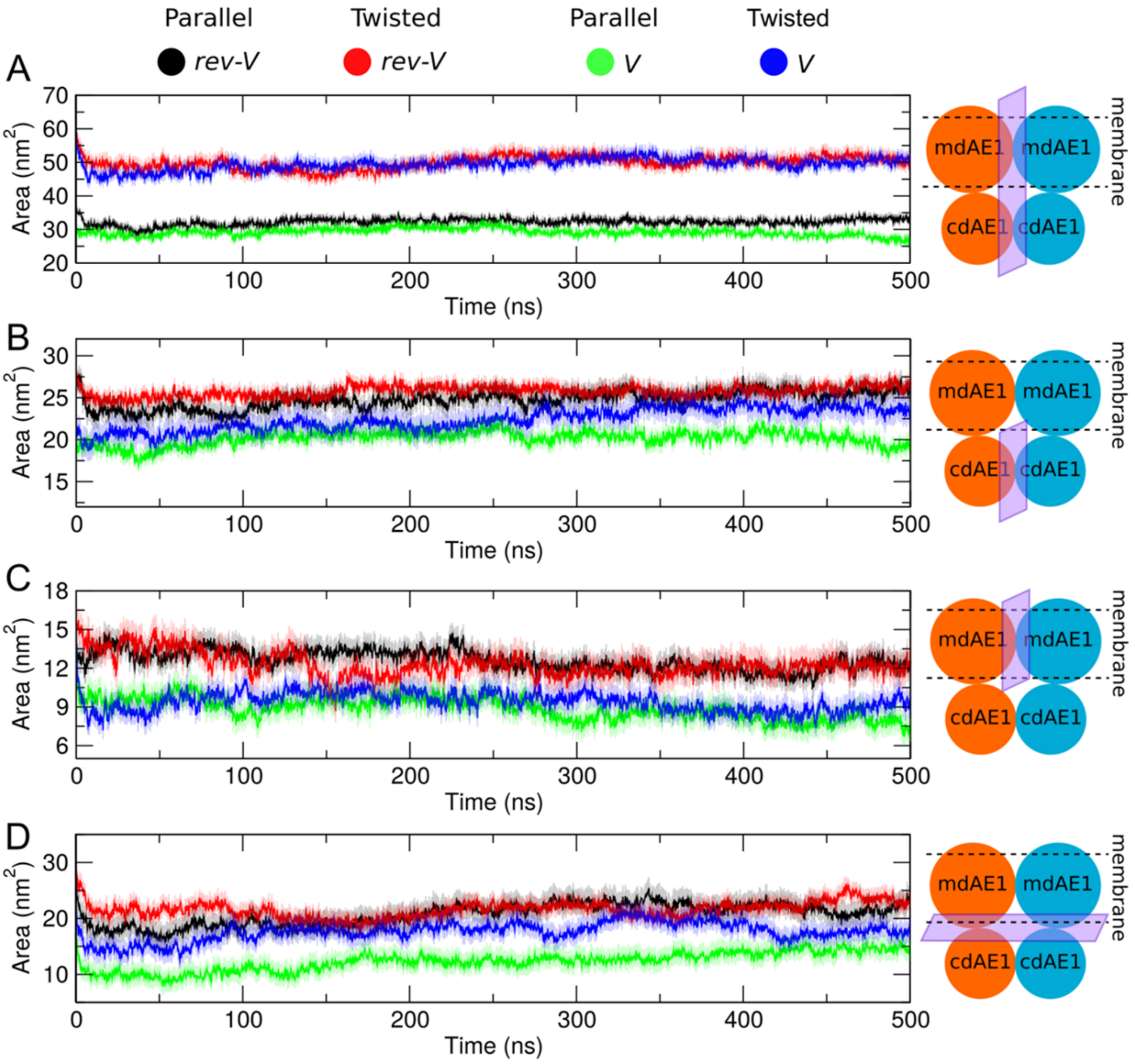

Figure S10. Buried surface area as a function of time for each of the AE1 conformers. Related to Table 1. Buried surface area of the AE1 dimer interface (A), the cdAE1.A/cdAE1.B (B), the mdAE1.A/mdAE1.B (C) and the dimeric cdAE1/mdAE1 (D). The cartoons on the right show the location of the calculated buried areas in a parallel orientation. In all plots the error bars are shown and each timeline represents the average over the five repeat CG simulations.

Table S2. H-bonds between the AE1 dimer in the atomistic simulations. Related to Table 1.

**Parallel *rev-V***	**Twisted *rev-V***	**Parallel *V***	**Twisted *V***
**Leu321.A – Leu319.B 99.29**	**Leu321.A – Leu319.B 99.07**	**Leu321.B – Leu319.A 99.45**	**Leu321.A – Leu319.B 98.96**
**Leu321.B – Leu319.A 98.16**	**Leu321.B – Leu319.A 95.47**	**Leu321.A – Leu319.B 99.36**	**Leu321.B – Leu319.A 95.46**
**Leu102.B – Val320.A 90.37**	His55.B – Asp887.A 86.15	**Phe597.B – Phe813.A 93.96**	Phe104.A – Ser318.B 87.44
**Leu319.A – Leu321.B 87.38**	Lys691.B – Asp205.A 56.51	Val320.B – Leu534.A 90.71	**Leu319.B – Leu321.A 80.17**
**Val320.A – Leu102.B 78.73**	Arg292.B – Asp887.A 55.62	**Val320.A – Leu102.B 86.95**	**Val320.A – Leu102.B 79.55**
Arg340.B – Aap325.A 54.26	Arg340.B – Glu329.A 55.26	**Leu319.A – Leu321.B 61.93**	Thr324.A – Glu291.B 68.50
**Phe597.A – Phe813.B 52.3**	Lys56.B – Asp887.A 52.31	**Leu319.B – Leu321.A 59.23**	**Val320.B – Leu102.A 63.04**
Arg340.A – Glu329.B 51.79	Val320.A – His101.B 51.56	**Leu102.B – Val320.A 56.52**	Lys691.A – Glu151.B 55.52
**Leu319.B – Leu321.A 48.72**	**Leu102.B – Val320.A 48.74**	Thr324.B – Gln339.A 54.61	**Leu102.B – Val320.A 54.30**
Thr324.B – Glu291.A 44.97	Arg340.A – Glu329.B 43.78	**Trp105.B – Glu312.A 44.97**	Lys691.A – Asp149.B 51.46
Asn569.B – Thr552.A 42.86	**Leu319.B – Leu321.A 43.49**	Phe104.A – Ser318.B 39.87	Lys353.B– Asp277.A 43.96
	Gln191.A – Asp363.B 41.48	Lys551.A – Asp626.B 39.72	Lys698.B – Glu238.A 42.03
			**Trp105.A – Asp316.B 40.34**
			**Trp105.B – Asp316.A 39.88**
			**Leu319.A – Leu321.B 39.70**

Values indicate the percentage of occurrence in the atomistic simulations for each conformer. Residues are donor and acceptor, respectively. Interactions highlighted in bold have been found in the crystal structures PDBs: 1HYN (Zhang et al., 2000), 4KY9 (Shnitsar et al., 2013) and 4YZF (4YZF) using UCSF Chimera with default settings (Pettersen et al., 2004).

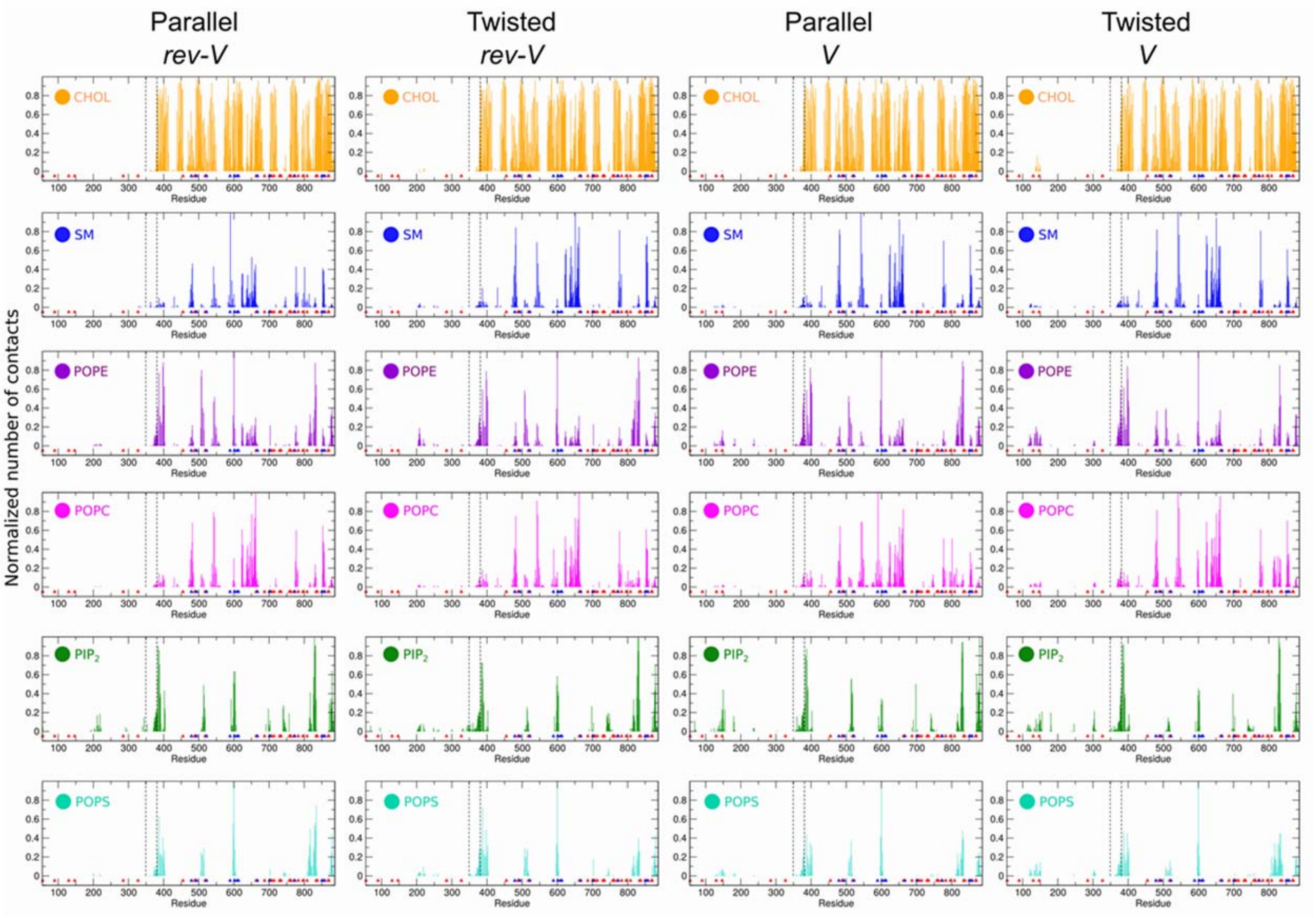

Figure S11. Normalized average number of contacts between AE1 and lipids. Related to Figures 5 and 6. The number of contacts between the protein and the lipids was averaged for each simulation system and merged for the two AE1 chains. The linker domain is shown by dotted lines. Point mutations reported to cause spherocytosis (SPH) or distal renal tubular acidosis (AD-dRTA) are indicated as *red* and *blue* triangles, respectively. Lipid species are: 1-palmitoyl-2-oleyl-phosphtidylcholine (POPC), 1-palmitoyl-2-oleyl-phosphtidylethanolamine (POPE), 1-palmitoyl-2-oleyl-phosphtidylserine (POPS), Phosphatidylinositol 4,5-bisphosphate (PIP_2), sphingomyelin (SM) and cholesterol (CHOL).

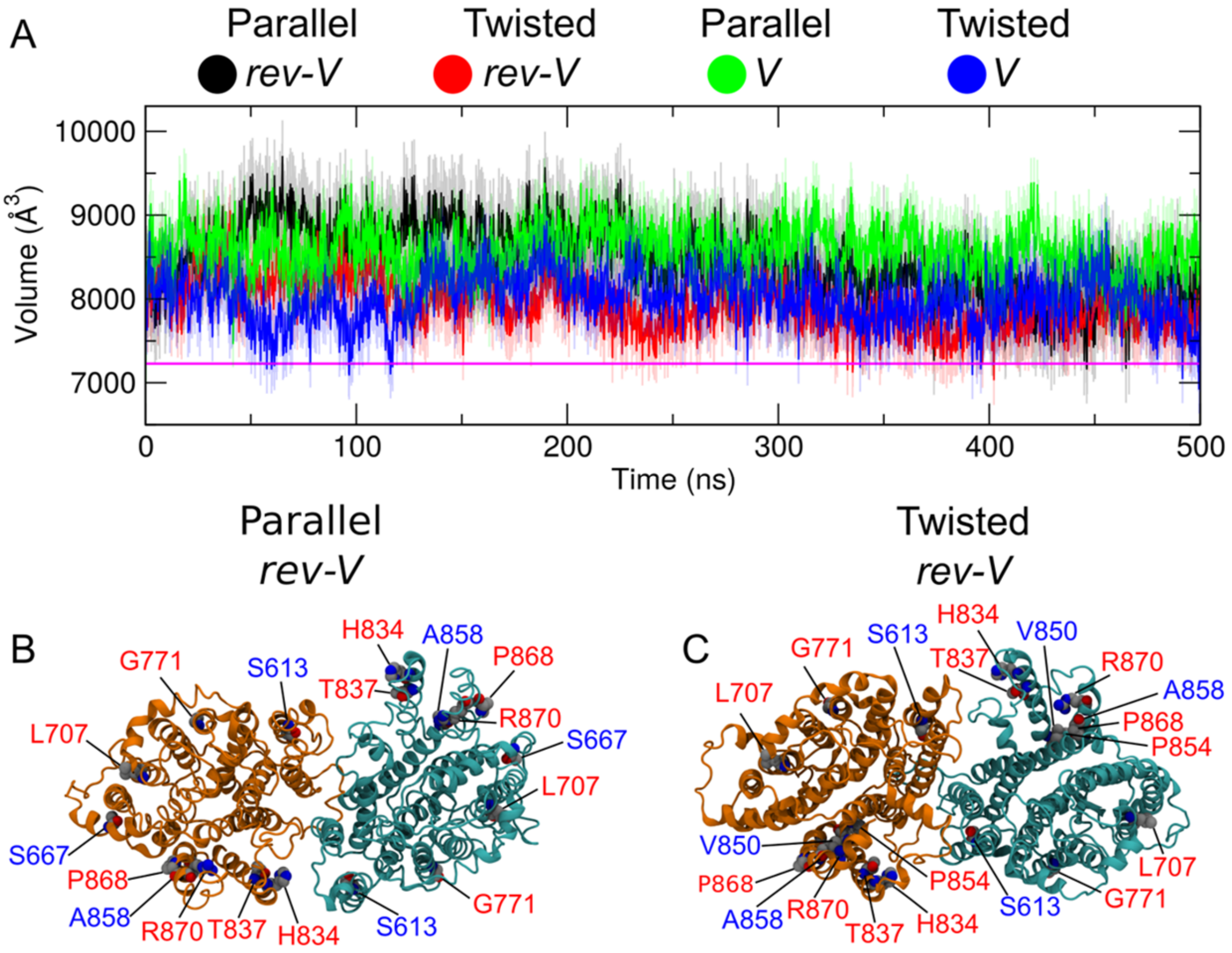

Figure S12. Cavity between the mdAE1 dimer interface and critical residues associated with diseases. Related to Figure 5. (A) Volume of the cavity at the interface of the mdAE1 in all conformers during the atomistic simulations. The calculation has been conducted considering residues 519-546, 569-26 and 785-816 as the boundary of the cavity. The *pink* line is the value calculated, over the same selection of residues, in the mdAE1 crystal structure. The error bars are shown and each timeline represents the average over the three repeat atomistic simulations. (B and C) Residues with normalized number of contacts with cholesterol above 0.5 which have been shown to cause human spherocytosis (SPH) and distal renal tubular acidosis (AD-dRTA) when mutated are shown in *red* and *blue*, respectively.

Table S3. H-bonds between AE1 and lipids in the atomistic simulations. Related to Figure 6.

Parallel *rev-V*		Twisted *rev-V*		Parallel *V*		Twisted *V*	
PIP_2 Lys600.B	95.82	PIP_2 Arg387.B	100	PIP_2 Arg832.B	99.31	PIP_2 Arg387.A	99.96
PIP_2 Arg879.A	95.04	PIP_2 Arg388.B	99.85	PIP_2 Arg387.A	98.71	PIP_2 Arg384.A	98.96
PIP_2 Arg603.A	93.82	PIP_2 Arg384.A	99.68	PIP_2 Arg388.A	94.33	PIP_2 Arg384.B	98.42
PIP_2 Lys600.B	95.55	PIP_2 Arg384.B	99.40	PIP_2 Arg827.B	89.46	PIP_2 Arg603.A	96.58
PIP_2 Lys829.A	93.42	PIP_2 Arg871.A	98.95	PIP_2 Arg871.A	84.37	PIP_2 Arg155.A	94.95
PIP_2 Arg384.B	92.55	PIP_2 Arg388.A	98.47	PIP_2 Lys829.A	75.62	PIP_2 Arg832.A	85.89
PIP_2 Lys600.A	91.89	PIP_2 Arg603.A	97.45	PIP_2 Arg514.A	74.71	PIP_2 Lys829.B	85.57
PIP_2 Arg387.B	91.84	PIP_2 Arg387.A	96.32	PIP_2 **Arg870.A**	72.99	PIP_2 Arg387.B	80.68
PIP2 Arg344.B	88.42	PIP_2 Arg344.B	91.50	POPE Arg387.A	72.88	PIP_2 Arg879.B	80.60
PIP_2 Arg832.A	73.93	PIP_2 Arg832.A	78.20	POPE Arg387.B	70.57	PIP_2 Lys600.A	78.37
PIP_2 Arg603.B	67.62	PIP_2 Arg832.B	77.29	CHOL**Gly714.B**	61.92	POPS Arg832.A	75.80
PIP_2 Arg384.B	66.57	PIP_2 Lys817.A	71.05	PIP_2 Lys829.B	61.19	PIP_2 Arg180.A	71.28
CHOL **His834.B**	60.79	PIP_2 Lys600.B	70.50	PIP_2 Lys817.B	60.17	POPC Arg646.A	67.53
PIP_2 Lys817.B	58.17	CHOL **His834.B**	69.41	PIP_2 Arg879.A	56.59	SM Arg782.A	66.97
PIP_2 Arg832.B	58.13	PIP_2 Lys829.A	69.14	PIP_2 Arg832.A	53.44	POPS **Arg870.A**	66.66
POPE Arg387.A	55.28	PIP_2 **Arg602.A**	65.96	POPE Arg832.B	46.79	PIP_2 Lys826.B	62.12
PIP_2 Arg340.B	53.70	PIP_2 Arg514.B	65.74	POPS Lys600.A	43.94	POPE Arg832.B	61.48
PIP_2 Val383.B	50.06	PIP_2 Gly599.B	65.56	PIP_2 **Arg518.A**	43.70	PIP_2 Trp831.A	56.99
SM Lys590.A	49.57	PIP_2 Arg514.A	65.36	POPE Arg827.B	43.21	PIP_2 Arg388.A	56.30
POPS Arg832.B	47.81	PIP_2 Lys829.B	64.02	POPC Lys590.A	42.66	POPS Lys829.A	56.21
POPE Glu658.B	45.14	PIP_2 Lys698.A	59.34	POPS Lys600.B	41.99	POPE Arg387.A	55.61
POPE Arg832.A	42.85	PIP_2 Arg879.B	58.71			PIP_2 Arg827.A	50.32
POPS Lys600.A	42.72	PIP_2 **Arg602.B**	58.67			POPS Lys817.A	48.23
POPS Arg514.B	42.28	POPS Lys600.B	58.56			PIP_2 Trp830.B	48.05
POPS Arg871.B	42.16	PIP_2 Trp831.A	56.61			POPS Lys600.B	46.57
PIP_2 Thr830.B	41.70	PIP_2 Arg827.B	55.19			POPS Glu882.A	43.76
PIP_2 Arg514.A	41.70	PIP_2 Arg345.B	54.34			POPS Lys600.A	39.34
POPS Arg879.B	41.37	POPC Arg782.A	49.61				
POPC Thr853.B	40.81	POPC Arg646.A	48.09				
POPC Arg387.A	40.43	PIP_2 Lys600.A	45.56				
POPE Arg879.A	39.94	PIP_2 Asn880.B	42.69				
SM Arg646.B	39.94						

Numbers are the percentage of occurrence in trajectories. Residues in red and blue cause SPH and AD-dRTA, respectively.
