# Supplementary material for: Molecular Simulations of Intact Anion Exchanger 1 Reveal Specific Domain and Lipid Interactions
Source: Biophys J. 2019 Aug 30;117(7):1364–79. doi: 10.1016/j.bpj.2019.08.029 (PMC6818359; doi:10.1016/j.bpj.2019.08.029)
Supplement: Document S2. Article plus Supporting Material [file mmc6.pdf]

Biophysical Journal
Article

Biophysical Society

Molecular Simulations of Intact Anion Exchanger 1 Reveal Specific Domain and Lipid Interactions

Dario De Vecchis,[1] Reinhart A. F. Reithmeier,[2] and Antreas C. Kalli[1,3,*]
[1]Leeds Institute of Cardiovascular and Metabolic Medicine, University of Leeds, Leeds, United Kingdom; [2]Department of Biochemistry, University of Toronto, Toronto, Canada; and [3]Astbury Centre for Structural Molecular Biology, University of Leeds, Leeds, United Kingdom

ABSTRACT Anion exchanger 1 (AE1) is responsible for the exchange of bicarbonate and chloride across the erythrocyte plasma membrane. Human AE1 consists of a cytoplasmic and a membrane domain joined by a 33-residue flexible linker. Crystal structures of the individual domains have been determined, but the intact AE1 structure remains elusive. In this study, we use molecular dynamics simulations and modeling to build intact AE1 structures in a complex lipid bilayer that resembles the native erythrocyte plasma membrane. AE1 models were evaluated using available experimental data to provide an atomistic view of the interaction and dynamics of the cytoplasmic domain, the membrane domain, and the connecting linker in a complete model of AE1 in a lipid bilayer. Anionic lipids were found to interact strongly with AE1 at specific amino acid residues that are linked to diseases and blood group antigens. Cholesterol was found in the dimeric interface of AE1, suggesting that it may regulate subunit interactions and anion transport.

SIGNIFICANCE Changes in proteins that are found in red blood cells may cause inherited diseases (e.g., anemias). For this reason, it is critical to understand how such proteins function at the molecular level. One such protein is anion exchanger 1 (AE1), which is responsible for the exchange of bicarbonate and chloride across the erythrocyte plasma membrane. In this study, we used computational modeling and simulations to build models of the intact AE1 in a model bilayer that mimics the native erythrocyte plasma membrane and discuss how mutations alter their function. Our results suggest that AE1 strongly interacts with specific lipids in the red blood cell membrane and highlight residues that, when mutated, may result in diseases.

INTRODUCTION

The red blood cell anion exchanger 1 (AE1, Band 3, SLC4A1) is responsible for the rapid exchange of bicarbonate and chloride across the red blood cell plasma membrane, a process necessary for efficient respiration (1,2). Human AE1 is a 911 residue glycoprotein consisting of a N-terminal cytoplasmic domain (cdAE1) that interacts with cytoplasmic proteins and a C-terminal membrane domain (mdAE1) responsible for its transport function (3). AE1 is found as a mixture of dimers and tetramers in membranes and in detergent solution (4,5). A poorly conserved linker region connects the mdAE1 with the cdAE1 that can be readily cleaved by trypsin at Lys360 separating the two domains (6). The mdAE1 monomer consists of 14 transmembrane (TM) helices organized as two inverted 7-helix repeat regions. In each monomer, helices TM1–4 and TM8–11 make up the core domain, whereas helices TM5–7 and TM12–14 form the gate domain (7). cdAE1 functions primarily as an anchoring site for cytoskeletal proteins such as ankyrin and is a major organization center of the red blood cell membrane (8). The cdAE1 monomer dimerizes with another monomer by a largely helical segment, the dimerization arm, in which a single β-strand plays a critical role (9,10). Mutations have previously been reported to alter AE1 function and localization to the plasma membrane, causing various diseases and conditions such as hereditary spherocytosis (6). Therefore, understanding the mechanistic details of the function of AE1 is physiologically and medically relevant.

Although crystal structures of the individual cdAE1 and the mdAE1 domains have been obtained in different crystallization conditions (7,9,10), a structure of the intact AE1 is not available. The presence of a flexible 33-residue linker region that connects the two domains makes it very challenging to identify the relative orientation of the cdAE1

Submitted May 13, 2019, and accepted for publication August 22, 2019.
*Correspondence: a.kalli@leeds.ac.uk
Editor: Philip Biggin.
https://doi.org/10.1016/j.bpj.2019.08.029
© 2019 Biophysical Society.
This is an open access article under the CC BY license (http://creativecommons.org/licenses/by/4.0/).

with respect to the mdAE1 and to obtain the structure of intact AE1 using lab-based structural methodologies. Denaturation of one domain does not alter the properties of the other domain (11), suggesting that the two domains are structurally independent. Accordingly, the anion transport function is retained after the proteolytic removal of the cytoplasmic domain (12–14), and the membrane domain is functional in transfected cells (10). However, several studies suggest structural and functional correlations between the two main domains. For example, binding of the anion transport inhibitor 4.4′-diisothiocyanostilbene-2,2′-disulfonate (DIDS) to mdAE1 from the cell exterior affects the interaction patterns of the cytoplasmic domain with hemoglobin (15), ankyrin, and spectrin (16). DIDS binding also results in the alteration of a quenching exposed cytoplasmic tryptophan (17). Moreover, the Memphis variant (K56E) in the cytoplasmic domain exhibits 20% reduction in anion transport, suggesting a functional interaction between the two domains (18–20). The cdAE1 also plays a critical role in the oligomerization of AE1 being essential for the formation of tetramers (5). For these reasons, obtaining structural data about the intact AE1 would be a major step in understanding the nature of interactions within the protein and with partner proteins like ankyrin.

Recently, a negative stain electron microscopy (EM) reconstruction using the bovine homolog revealed that the cdAE1 and mdAE1 are connected by a pillar-shape linker region (21). This study suggested two possible orientations for the cdAE1: twisted and parallel. In both orientations, the cdAE1 C-terminal region is oriented next to the linker density (21), possibly providing flexibility to the cdAE1 (22). In contrast, a recent modeling approach supported by cross-linking experiments proposed a more compact structure with the double-humped shape of cdAE1 facing the mdAE1 (23). In this model, the linker region appears to be not symmetrical. Therefore, the two aforementioned studies suggest very different orientations of the cdAE1 relative to the mdAE1. In the light of these discrepancies and given that it has been proven challenging to obtain a complete structure of AE1 using lab-based structural techniques, molecular dynamics simulations and modeling can be used to obtain novel structural data of the intact AE1, including the nature and dynamics of the interaction between the two domains. Constructing a complete model of the intact AE1 is critical as it will enable us to comprehend the complexities of the AE1 transport mechanism in the context of the intact protein, the formation of AE1 tetramers, and its interactions with partner proteins such as ankyrin (24,25).

In this study, we examined the interactions and dynamics of the cdAE1 and mdAE1 domains by developing a near full-length model of the red cell anion exchanger AE1 in the outward-facing conformation. The N- and C-terminal regions of AE1 (residues 1–54 and 888–911, respectively) that are predicted to be disordered are omitted from the models as was the inhibitor DIDS located at the interface of the core and gate domains in the crystal structure. Our results demonstrate the role of the linker region in mediating the interactions between the two AE1 domains and propose possible orientations of the cytoplasmic domain with respect to the membrane domain. Additionally, the models of the intact AE1 were simulated in a native-like model red blood cell plasma membrane, containing a full complement of phospholipids as well as cholesterol and sphingomyelin (SM) (26,27). Our results show that the intact AE1 interacts preferentially with anionic phospholipids present in the inner leaflet and that cholesterol is likely to stabilize the AE1 dimer interface. Residues, including those in the linker region that are involved in the interaction between the cdAE1 and the mdAE1 domain, were identified. Interestingly, a number of residues involved in domain interactions and interactions with lipids are sites of mutations linked to human diseases.

METHODS

Sequences alignments and predictors

The AE1 (Band 3, SLC4A1) protein sequence from *Homo sapiens* was obtained from the Universal Protein Resource Knowledgebase database ((28); entry: P02730). The tool DISOPRED v.2 and v.3 were used to predict disordered residues in AE1 protein sequence (29). The secondary structure of AE1 was predicted from its sequence using CONCORD (30) PsiPred (31), and PSSpred (32). The National Center for Biotechnology Information HomoloGene database (https://www.ncbi.nlm.nih.gov/homologene accessed 26-11-2018) was used to select three sets of homologous members from different species of AE1, AE2, and AE3 belonging to the SLC4 family (hgid: 133556, 128699, and 129474, respectively). They have been selected because they all mediate anion (Cl^-/HCO_3^-) exchange (33–35). The T-Coffee method (36) was used to align these sets of protein sequences.

Modeling of the intact AE1 conformers

The coordinates of the mdAE1 (residues 382–887) were obtained from the crystal structure Protein Data Bank (PDB): 4YZF (7,37). The models were built without the inhibitor DIDS that is present in the crystal structure. To model the cdAE1 (residues 55–348), we combined the coordinates from the two crystal structures 1HYN (9) and 4KY9 (10) that were crystallized under different pH conditions and added the missing regions using MODELLER (v 9.19) (38,39). Models were ranked according to the Discrete Optimized Protein Energy (DOPE) method (40), selecting the best model out of 20 candidates. For each conformer presented in Fig. 1, the cdAE1 domain was aligned in the *z* axis and manually positioned on the bottom of mdAE1 as suggested by the EM structure for the bovine homolog (21). The dimeric cdAE1 and mdAE1 were connected by adding the linker regions (residues 349–381) using the loop modeling routine of MODELLER (38,39). Ten models of the linker region were generated for each subunit of the dimeric AE1. We used the DOPE method to select the best loop configuration (40). Note that the final models were also carefully checked for inconsistencies. In the case of the *rev-V* twisted conformer, we excluded the best model suggested by the DOPE score because the linker of one of the AE1 monomers was knotted around the cdAE1. Similarly, for the *V* twisted, we excluded the best model suggested by the DOPE score because the linker of one monomer was passing throughout the V-shaped cavity of the cdAE1. The variation in the conformations of the linker models (10 for each monomer) generated during the modeling stage

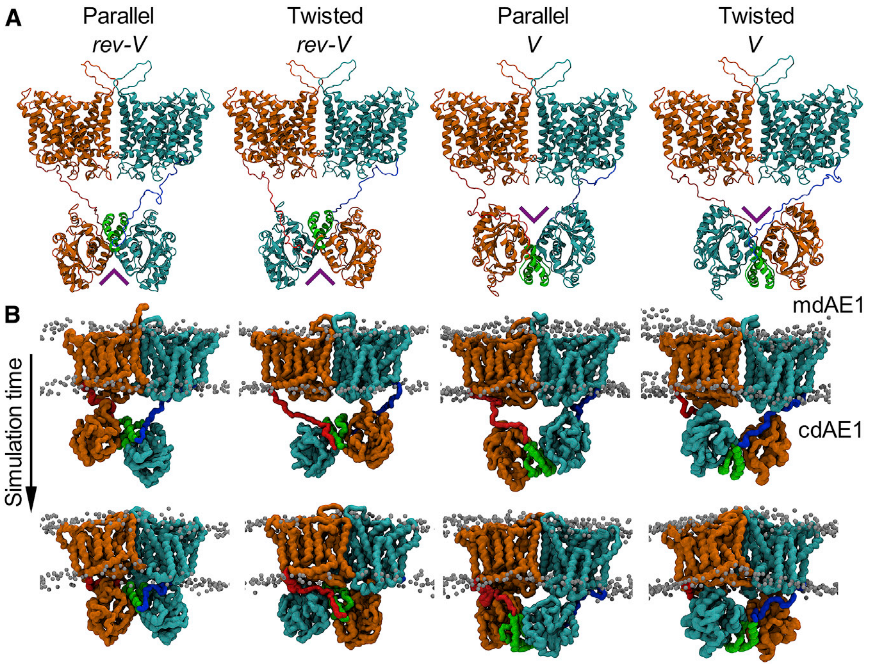

FIGURE 1 The four different AE1 configurations used in the simulations. (*A*) Each structure is a side view of the dimeric AE1 conformations after the initial model building (see Methods) showing the relative positions of the mdAE1 (*upper*) and cdAE1 (*lower*). The two AE1 subunits are represented in orange and cyan ribbon diagrams with the linker regions in red and blue, respectively. The cdAE1 C-terminal dimerization arms (residues 314–347) are shown in green, facing the mdAE1 (i.e., *rev-V* conformers) or the cytoplasmic side (i.e., *V* conformers). A purple arrow shows the direction of the V-shape groove formed by the cdAE1 dimerization arms. (*B*) Snapshots from different time points during the CG-MD simulations show the interaction of cdAE1 and mdAE1. Phosphate groups from the lipid bilayer are shown as gray spheres. See also Figs. S1 and S2. To see this figure in color, go online.

for each AE1 conformer is shown in Fig. S1 *A*. The resulting final distance between the dimeric cytoplasmic and membrane domains is ~24 and ~20 Å for the *rev-V* and the *V* conformers, respectively. This is approximately the distance detected in the three-dimensional map of the bovine AE1 (i.e., 30 Å) (21). All the models were energy minimized in vacuum before running the simulations using GROMACS 5.0.7 (41).

Coarse-grained molecular dynamics simulations

The coarse-grained molecular dynamics (CG-MD) simulations were performed using the Martini 2.2 force field (42,43) and GROMACS 5.0.7 (41). Each AE1 conformer, obtained as described above, was converted to a coarse-grained resolution and energy minimized. To model the protein secondary and tertiary structure, an elastic network model with a cut-off distance of 7 Å was used. The elastic network model used in this study is a network that is fairly standard for simulations with the Martini force field, and it was used in a large number of studies that showed good agreement with experimental data, including our previous study on AE1 (37,44). The distance between the dimeric cytoplasmic and membrane domains is greater than the aforementioned 7 Å cut-off distance that was used for the elastic network model (~24 and ~20 Å for the *rev-V* and the *V* conformers, respectively). Therefore, no elastic bonds are present between the TM and the cytoplasmic regions, but elastic bonds were present within the TM or the cytoplasmic region. Regarding the linker region, out of the 33 residues that comprise each linker region, 10 and 15 residues from each linker in the *rev-V* parallel, 9 and 6 residues from each linker in the *rev-V* twisted, 7 and 12 residues from each linker in the *V* parallel, 6 and 7 residues from each linker in the *V* twisted participate in elastic bonds. Most of these residues are in the N- and C-terminal that connects the linker with the TM or the cytoplasmic domains. It should be noted that our analysis also demonstrates the flexibility of the linker regions during the simulations (see Fig. S1 *C*; Videos S1, S2, S3, and S4). For the simulations, each conformer was inserted in a complex asymmetric bilayer using the INSert membrANE tool (45). The bilayer lipid composition used in the simulations is described in Table S1. Systems were neutralized with a 150 mM concentration of NaCl. Before the production simulation, each conformer was energy minimized and subsequently equilibrated for 10 ns with the protein particles restrained (1000 kJ $\times$ mol^{-1} $\times$ nm^{-2}) to allow the membrane bilayer to equilibrate around the AE1 models. Five repeat simulations starting from different initial velocities were simulated for a total of 5 μs with an integration step of 20 fs. All simulations were performed at 323 K, with protein, lipids, and solvent separately coupled to an external bath using the V-rescale thermostat (46) (coupling constant of 1.0). The temperature of 323 K is above the transition temperatures of all lipid species in the systems, therefore avoiding the lipids to undergo phase transitions to the gel phase. Pressure was maintained at 1 bar (coupling constant of 1.0) with semi-isotropic conditions and a compressibility of 3×10^{-6} bar^{-1} using the Berendsen barostat (47). Lennard-Jones and Coulombic interactions were shifted to zero between 9 and 12 Å and between 0 and 12 Å, respectively.

To test the stability of the mdAE1/cdAE1 complex in the absence of the linker region, CG-MD simulations were also initiated in which the linker region of each monomer (residues 349–381) was removed from the centroid of the first cluster of each system. These simulations were run using the same parameters as above. For each model, five repeat simulations were run for 5 μs.

Atomistic molecular dynamics simulations

Snapshots from the CG-MD simulations were selected using cluster analyses and converted to atomistic resolution as described in (48). The atomistic simulations allowed us to examine the stability and conformational dynamics of our AE1 models, thus addressing some of the limitations of our CG-MD simulations. Before the production simulations, the atomistic systems were minimized and subsequently equilibrated for 30 ns with the backbone atoms restrained (1000 kJ $\times$ mol^{-1} $\times$ nm^{-2}). Three repeat simulations of the same system were simulated starting from different initial velocities. Simulations were run using the CHARMM36 force field (49) for 500 ns with an integration step of 2 fs. The first 100 ns of each trajectory were not used in our analyses. The V-rescale thermostat (46) and the Parrinello-Rahman barostat (50) were used for temperature and pressure control (semi-isotropic). The reference temperature was 323 K, and the reference pressure was 1 bar (compressibility of 4.5×10^{-5} bar^{-1}). Verlet was used as the cut-off scheme, with the neighboring list updated every 20 steps. The cut-off distance for Lennard-Jones was 12 Å. Long-range electrostatics were managed using the particle-mesh Ewald method, with a cut-off

distance of 12 Å (51). The LINCS algorithm was used to constrain bond lengths (52).

Trajectory analyses and molecular graphics

Root mean-square fluctuation (RMSF) analysis was performed using the tool g_rmsf from the GROMACS package. Radius of gyration, distances, buried surfaces, and contact analyses were also performed using tools from the GROMACS package (g_gyrate, gmx distance, gmx sasa, and g_mindist, respectively). Hydrogen bonds (within a distance cut-off of 3.5 Å and up to 30° off-axis angle) and salt bridges (within a cut-off distance of 5.5 Å) were identified using g_hbond from GROMACS and VMD 1.9.3 (53) (http://www.ks.uiuc.edu/Research/vmd/), respectively. H-bonds on the crystal structures were identified with UCSF Chimera 1.12 (54). Other analyses were conducted using in-house scripts. The cluster analysis was performed using g_cluster from GROMACS with the GROMOS method (55). For this analysis, all five repeat simulations were concatenated, and the analysis was performed on the resulting 25-μs (5 $\times$ 5 μs) trajectory for each conformer. Because of the differences between conformers (i.e., different orientation of the cytoplasmic domain and linker regions), we have used a RMSD cut-off distance of 2.5 Å to be able to pick more subtle differences between the conformations of the linker region. Calculation of the volume of cavities was done using the trj_cavity tool (https://sourceforge.net/projects/trjcavity/) (56). Note that for these calculations, we have used .ndx files consisting of selections of AE1 protein regions. The error for the volume of the cavity is the SD of the volumes between the repeat simulations. The electrostatic potentials in aqueous solution at pH 7 was calculated using APBS (57). Molecular graphics were generated with the VMD 1.9.3 (53) (http://www.ks.uiuc.edu/Research/vmd/). Data were plotted using Grace (http://plasma-gate.weiz-mann.ac.il/Grace/).

RESULTS

Models of the intact AE1

The starting point for our study was to create a model of the intact AE1 using the available structures of cdAE1 (residues 55–348) and mdAE1 (residues 382–887) connected by a selected model of the linker region (residues 349–381). Using the orientations proposed for the cdAE1 relative to the mdAE1 from previous studies (21,23), two possible configurations were investigated: the cdAE1 C-terminal dimerization arms (residues 314–347) facing the mdAE1 or facing the cytoplasm, hereby called *reversed-V* (*rev-V*) and *V* conformation, respectively, because of the characteristic V-shape groove formed by the cdAE1 helices (purple arrow in Fig. 1 *A*).

The length of the linker region connecting the mdAE1 with the cdAE1 (33 residues) also allowed positioning of the cdAE1 in a parallel or a twisted orientation as suggested by a previous EM study (21) in both the *rev-V* and *V* orientations. Therefore, we simulated four different starting configurations (Fig. 1 *A*). This does not preclude that there is a dynamic interconversion of these states that changes the orientation of the cdAE1 and mdAE1. The N- and C-terminal regions of AE1 (residues 1–54 and 888–911, respectively) that are predicted to be disordered are omitted from the model (see Methods). The formation of the mdAE1/cdAE1 complex was investigated by CG-MD simulations in a model membrane consisting of a complex lipid mixture (Table S1). The purpose of this study was to determine whether there is a unique orientation of the cdAE1 and the mdAE1 and to identify key residues involved in the interaction, including the role of the linker.

Despite the differences in the initial orientation of the cdAE1 relative to the mdAE1, in all cases, the cdAE1 moves closer to the membrane and forms a complex with the cytoplasmic side of the mdAE1 domain, resulting in a more compact structure (Fig. 1 *B*). Calculation of the distances between the centers of mass of the cdAE1 and the mdAE1 showed that the cdAE1 interacts with the mdAE1 domain at the early stages of the simulations. The cdAE1/mdAE1 distance for the twisted (*rev-V* and *V)* conformers was ~0.25 nm smaller compared to the parallel (*rev-V* and *V)* conformers (Fig. S2 *B*). This is probably because a parallel orientation of the linker region provides more flexibility to the cdAE1, whereas the twisted orientation allows tighter packing of the cdAE1 to the mdAE1. Cluster analysis and calculation of the radius of gyration across all simulations of each conformer confirms that the structures increased their compactness with respect to the initial coordinates shown in Fig. 1 *A*, with the *V* parallel being the least compact conformer (Fig. 2; Fig. S2 *C*).

For the cluster analysis, a RMSD cut-off of 2.5 Å was used (see Methods), resulting in a large number of clusters, which allowed us to annotate a wide range of subtle differences between the conformations of the linker region (Fig. S1 *C*). The first cluster contained the majority of structures in all systems, showing that these protein conformations were the most observed within the simulation ensemble (Fig. S3). The snapshot in the center of the first cluster was used to initiate our atomistic simulations for all systems. The breakdown of the clusters is the following: for the *rev-V* parallel, we found 187 clusters with the first cluster containing 7080 structures (14.2%). For the *rev-V* twisted, we found 229 clusters with the first cluster containing 6473 structures (13.0%). For the *V* parallel, we found 267 clusters with the first cluster containing 5571 structures (11.1%). For the *V* twisted, we found 171 clusters with the first cluster containing 5652 structures (11.3%). Moreover, a more detailed analysis of the occurrence of each cluster in the repeat simulations within the simulation ensemble suggests that the structure from the top cluster from each individual repeat simulation is very similar to the structure used to initiate our atomistic simulations (overall top cluster; Figs. S4–S7). Our cluster analysis also enables us to calculate the RMSD of the centroid of each cluster with respect to the backbone atoms of the first centroid and then calculate the lowest and highest value of the RMSD observed in the clustering analysis. For the *rev-V* parallel, the minimal RMSD was 2.202 Å, and the maximum was 14.348 Å. For the *rev-V* twisted, the minimal RMSD was 1.271 Å, and the maximum was 15.275 Å. For the *V* parallel, the minimal RMSD was

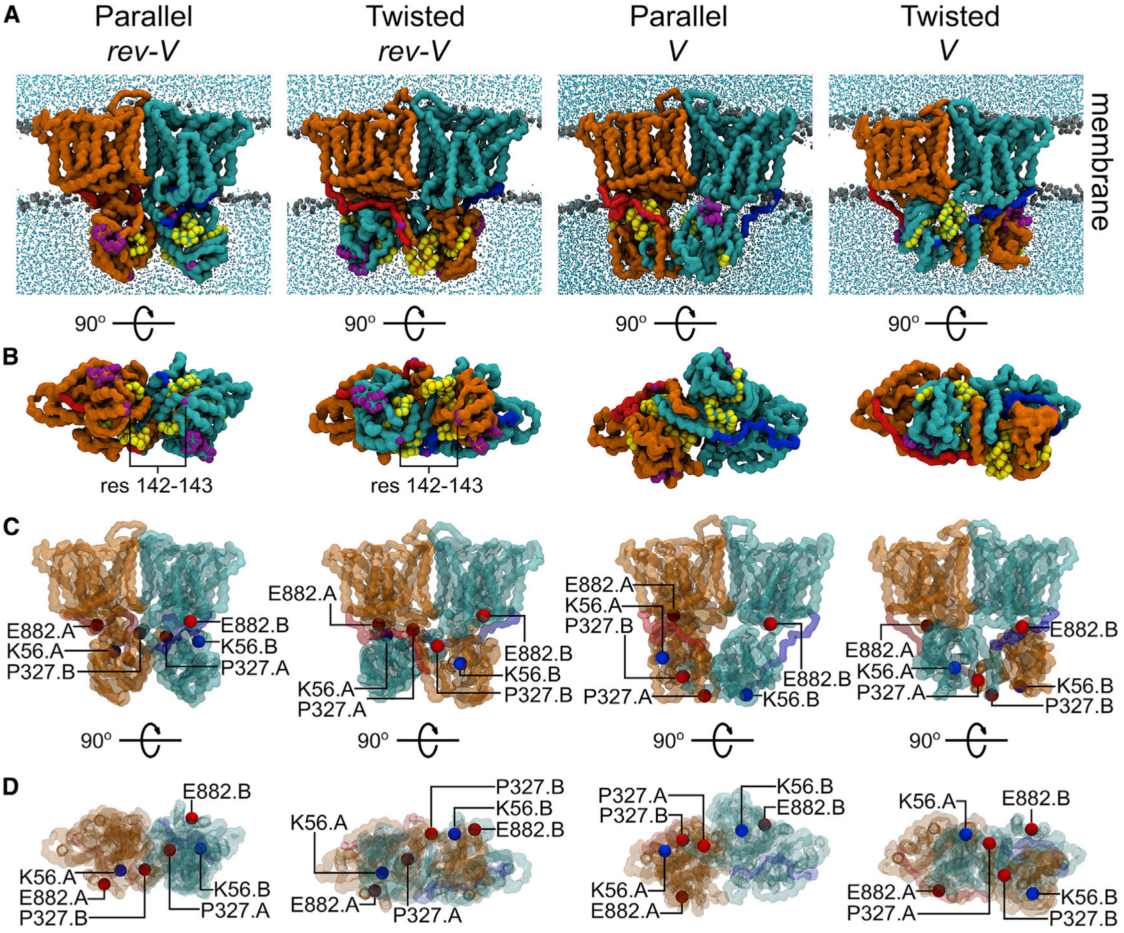

FIGURE 2 The AE1 configurations resulting from the simulations. (*A*) Structures represent the most sampled conformation (i.e., a centroid) for each conformer in the CG-MD simulations. The surface of the backbone particles of the AE1 complex dimer subunits are shown in orange and cyan with the linker regions in red and blue. Phosphate groups from the lipid bilayer and water molecules are shown in gray and cyan, respectively. (*B*) Shown is the cytoplasmic view of the systems in (*A*). Residues shown in other studies to interact with actin (residues 258–311) and ankyrin (63–73, 142, 143, 175–185, 353) are shown as yellow and purple spheres, respectively. (*C*) The position of the variants Memphis (K56E) (18,64,65) and Tuscaloosa (P327R) (20) is shown on the structures from A as blue and red spheres, respectively. (*D*) Shown are the cytoplasmic views of the systems in (*C*). The E882 residue, closer to the Tuscaloosa variant in the *rev-V* conformers, is also shown. See also Figs. S1 and S3–S8. To see this figure in color, go online.

2.520 Å, and the maximum was 13.473 Å. For the *V* twisted, the minimal RMSD was 2.512 Å, and the maximum was 13.157 Å.

To identify which of our models above most likely represents the native cdAE1/mdAE1 complex, we have examined where the binding sites of the cdAE1 to the cytoskeletal proteins are relative to the mdAE1 domain in each model. Exposed loops on the cytoplasmic domain (residues 63–73 and 175–185; purple spheres in Fig. 2, *A* and *B*) were previously reported to be involved in red cell membrane integrity via interactions with ankyrin (58–60). These residues are located on the side of the cdAE1, thus exposed to the solvent in all our models described above.

The recently discovered ankyrin cross-linking site (residues 142–143) (23) that is located at the tip of the cdAE1 (purple spheres in Fig. 2, *A* and *B*) is only exposed to the cytoplasm in the *rev-V* conformers in our models (Fig. 2 *B*). Additionally, the dimeric form of AE1 has been found to associate with the actin junctional complexes (61), with residues 258–311 suggested as the possible site of interaction between AE1 and cardiac α-actin (62). Residues 258–311 are located in close proximity to the V-shape groove formed by the dimeric cdAE1 (yellow spheres in Fig. 2, *A* and *B*). This groove in our models faces the cytoplasmic side in the *rev-V* conformers, and it is rather hidden in the *V* conformers.

AE1 has been also shown to interact with protein 4.2. Nearby residues Glu882, Asp889, and Asp890 were suggested to be part of a mdAE1 negative patch that would interact with protein 4.2 (23). Mapping the position of Glu882 in our models suggests that this residue is closer to the AE1 natural variant Tuscaloosa (P327R in cdAE1 (20)) only in the *rev-V* conformers, whereas it is rather distant in the *V* conformers (Fig. 2 *C*, red spheres). The Tuscaloosa variant has been shown to decrease the interaction with protein 4.2 by 29% by introducing an arginine in

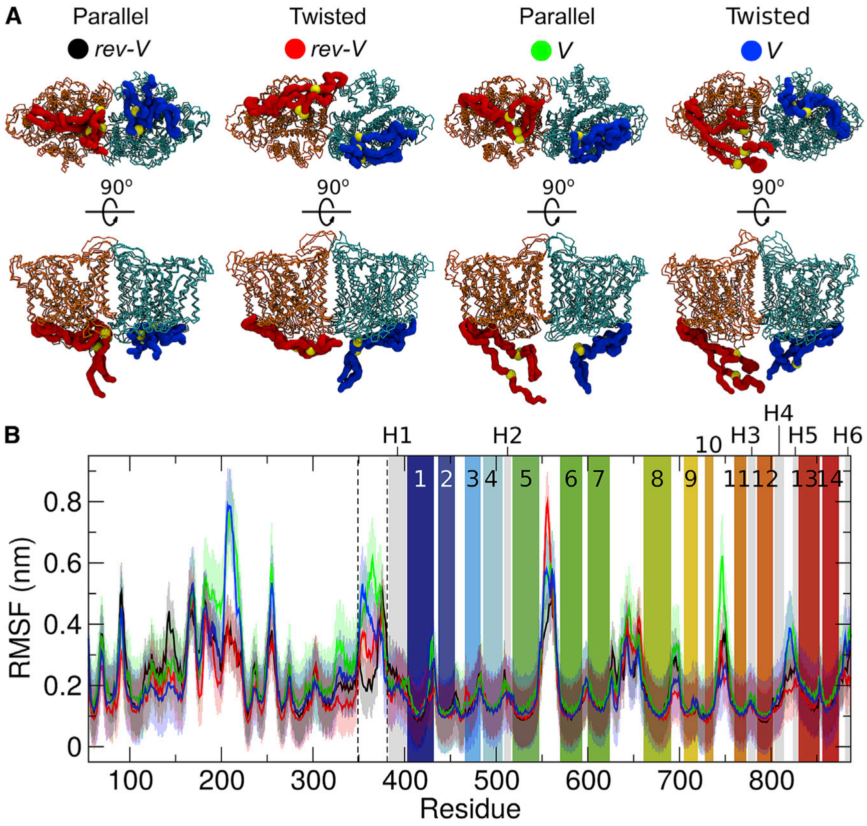

FIGURE 3 AE1 linker position. (*A*) The AE1 structures are the three most populated centroids after the cluster analyses of the CG-MD simulations. The protein has been aligned on the mdAE1 backbone atoms. The backbone particles of the mdAE1 are shown in orange and cyan with the linker regions in red and blue. The residue K360 is shown as a yellow sphere. The cdAE1 domain has been omitted for clarity. (*B*) Shown is the root mean-square fluctuation (RMSF) of the intact AE1 in the atomistic simulations. The mdAE1 TM helices (TM1 to TM14) are highlighted in rainbow, and the reentrant helices are in gray (H1 to H6). The residues of the linker regions are shown between the dotted lines. Each line represents the average and the SD over the three repeat atomistic simulations of each system. To see this figure in color, go online.

cdAE1 (20,63). This arginine may interfere with the aforementioned negative patch in mdAE1. These observations augment our previous suggestion that the *rev-V* conformers are more in agreement with available data.

Furthermore, in our models, Lys56, which is involved in the Memphis variant (i.e., K56E in cdAE1) (18,64,65), is located at the interface between the cdAE1 and mdAE1 only in the *rev-V* conformers, but it faces the cytoplasmic side in the *V* conformations (Fig. 2, *C* and *D*, blue spheres). This variant exhibits a 20% decrease in anion transport (19). The negative charge introduced by the glutamate in the Memphis variant may reduce the capacity to concentrate anionic molecules near the channel mouth (9). These functional data also suggest that the *rev-V* conformation is the more likely orientation.

Finally, the AE1 linker region can be readily cleaved by mild trypsin treatment at Lys360 (66,67). The position of the Lys360 is rather buried in all of our models, especially in the *rev-V* conformers in which the increase in compactness brings the linker very close to the mdAE1 domain (Fig. 3). The Lys360 is located approximately in the middle of the linker domain and is buried in the simulations only when the cytoplasmic domain is in complex with the TM region. The precise stage in which the trypsin cleavage occurs is currently unknown, and thus cleavage may occur in conformations in which the cdAE1 is not in a tight complex with the mdAE1 (Fig. S1 *B*). Hence, the ready exposure of this residue to trypsin cleavage suggests that although there may be one energetically preferred conformation, the interaction of cdAE1 and mdAE1 might be rather dynamic, exposing Lys360 to solvent.

We have also run CG-MD simulations in which the linker region (residues 349–381) from the first centroid of each conformer from all our models was removed (see Methods). Five repeat simulations of 5 μs each were run under the same conditions as our previous CG simulations (see Methods). Our analysis (Fig. S8) indicates that in the absence of the linker region, the mdAE1/cdAE1 complex is fairly stable. Among the four conformers, the *V* twisted structure has the highest RMSD relative to the starting structure, but alignments of the final snapshot of the simulations with the starting structure demonstrates that no major changes are observed within the mdAE1/cdAE1 complex. Interestingly, the compactness of all conformers was somewhat increased, with the *V* parallel to have the highest radius of gyration and distance between the cdAE1 and mdAE1 in the presence and absence of the linker region. Thus, the linker region may not play a major role in mediating the interaction between the two domains.

Overall, our comparisons above suggest that the *rev-V* conformation is the most consistent with available biochemical and functional data. Moreover, although it does not

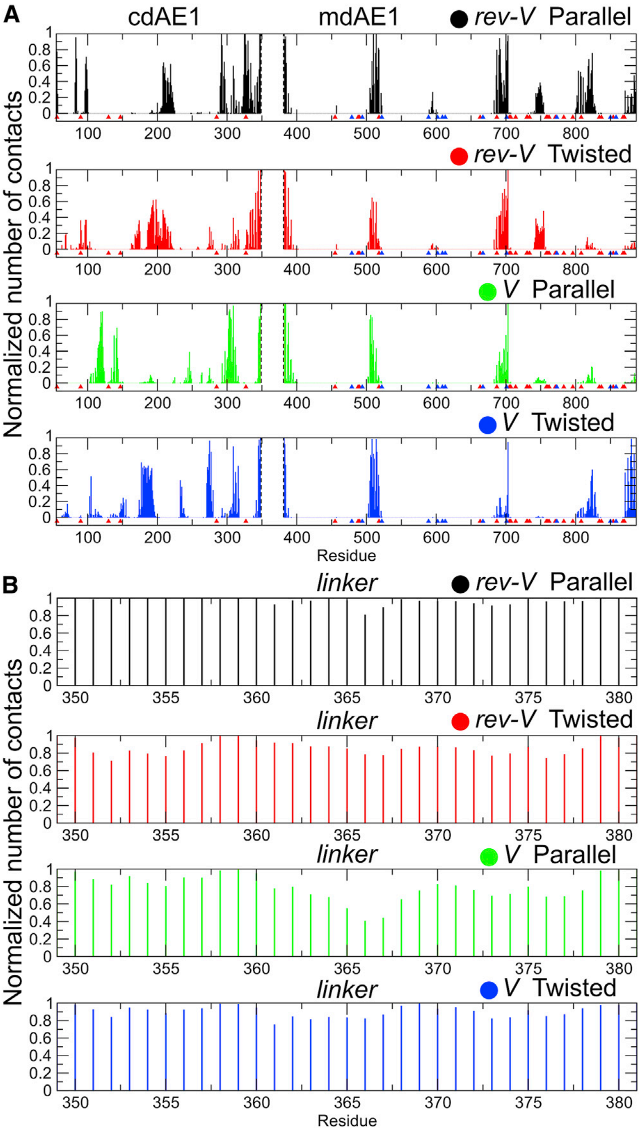

FIGURE 4 Interactions of the linker region with the cdAE1 and the mdAE1 domains. Shown is the normalized average (over all repeat CG-MD simulations) number of contacts between the cdAE1 or the mdAE1 (shown in (*A*)) and the linker region (shown in (*B*)). For this analysis, the contacts of each AE1 chain were added together. Values close to 0 represent no/low number of contacts, whereas 1 represents a high number of contacts. A cut-off distance of 0.55 nm was used. Note that for clarity, the linker region was not included in the analysis in (*A*), and for this reason, no contacts are shown between the dotted lines. Point mutations reported to cause spherocytosis (SPH) or distal renal tubular acidosis (AD-dRTA) are indicated as red and blue triangles in (*A*), respectively. See also Fig. S9. To see this figure in color, go online.

occur during our simulations, it is possible that because of the long linker, the cdAE1 is dynamic, and there is an interconversion of the parallel and twisted conformations in the native protein regulating the interaction of AE1 with partner proteins. The dynamic nature of the linker supports the view that it is not a major mediator of cdAE1-mdAE1 interactions.

Interactions between AE1 cytoplasmic and TM domains

Coarse-grained simulations

Fig. 3 *A* shows the most likely conformations adopted by the linker region during the simulations. Our analysis for the likely conformations adopted by the linker region reveals a rather flexible behavior in the *V* conformations but less flexibility of the linker in the *rev-V* conformations (see Fig. 3 *A*). Note that the flexibility of the linker region is observed both in the coarse-grained and in the atomistic simulations (Fig. 3, *A* and *B*). Additionally, our analysis suggests that the linker interacts with both the cdAE1 and the mdAE1 after the formation of the cdAE1/mdAE1 complex (Fig. 4) and, thus, may play some role in regulating the interactions between the two domains. Particularly in the *rev-V* parallel conformer, the linker interacts with the cdAE1 N-terminal residues 55, 56, 83, 292, and 293 and is also positioned next to residues 325–327, 330, 347, and 348 located in the dimerization arms (residues 314–347; Fig. 4 *A*). In the *rev-V* twisted conformer, the linker interacts strongly with residues 194, 198, 341, 344, 345, and 348. Our contact analysis, using a cut-off distance of 0.55 nm, suggests that the majority of the linker residues interact with the rest of AE1 in all conformers (Fig. 4 *B*). Interestingly, the natural variant Tuscaloosa (P327R (20)) is in contact with the linker in the *rev-V* parallel conformation. Structural studies of the isolated cdAE1 have shown that the mutation P327R directly affects the binding of the cytoskeletal protein 4.2 without destabilizing the cdAE1 dimeric structure (68). Therefore, the introduction of a positively charged residue in position 327 may also affect its interaction with the linker region.

The mdAE1 domain mostly interacts with the linker via the helical segments that connect the TM helices, particularly H2, H4, H5, H6, and the loop between TM8/9 (Fig. 4) in both the *V* and *rev-V* conformations. Within this latter region, the Gly701 when mutated in Asp (G701D) is reported to impair AE1 expression at the cell membrane and cause the autosomal dominant distal renal tubular acidosis (AD-dRTA) (69–71).

Analysis of the interactions between the two monomers of the intact AE1 dimer suggests that in both the parallel and the *rev-V* twisted conformers, residues belonging to the cdAE1 dimerization arms form the largest number of interactions between the AE1 monomers (residues 314–347, Fig. S9 *A*). This suggests that in AE1, the largest dimerization interface is found in the cdAE1 and not in the mdAE1. The cdAE1/cdAE1 interactions are formed mainly via residues 98–110, nearby residue 200, and residues 320–340 (Fig. S9 *A*). Most of the interactions between the mdAE1 monomers are formed between residues 593–600, 623–628, 690–705, and 813–824. For the residues that are embedded within the bilayer, Phe583, which is located in

TABLE 1 Buried Surface Area of the Dimer Interface of the Four Conformational States

	Parallel *rev-V*	Twisted *rev-V*	Parallel *V*	Twisted *V*
(*A*) Buried AE1 dimer interface	32.19 ± 1.02	49.92 ± 1.92	29.18 ± 1.11	49.39 ± 1.83
(*B*) Buried cdAE1.A/cdAE1.B	24.73 ± 1.03	25.74 ± 0.66	20.14 ± 0.85	22.38 ± 1.32
(*C*) Buried mdAE1.A/mdAE1.B	12.61 ± 0.77	12.31 ± 0.93	8.84 ± 0.83	9.38 ± 0.73
(*D*) Buried dimeric cdAE1/mdAE1	20.71 ± 1.90	21.6 ± 1.39	12.29 ± 1.68	17.6 ± 1.67

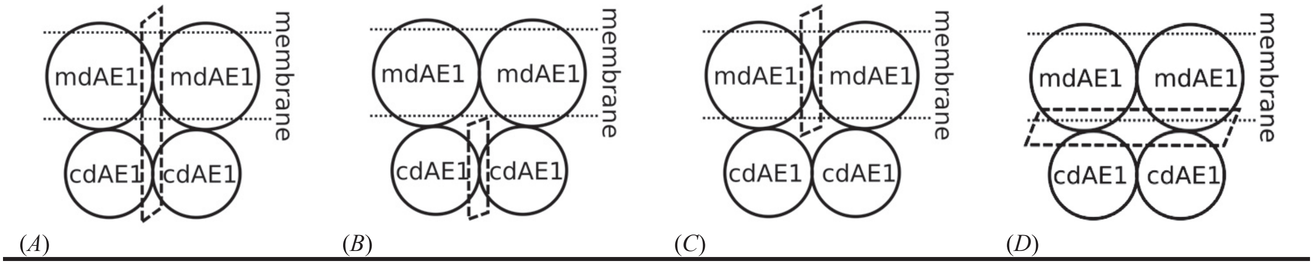

Values are as follows: mean surface (nm^2) ± SD. See also Fig. S10 and Table S2.

the middle of the TM 6, forms the highest number of mdAE1/mdAE1 interactions. Interestingly, in our simulations, even though predicted to be disordered, the linker region is part of the AE1/AE1 dimer interface in all conformers except for the *V* parallel (Fig. S9 *A*, green histograms).

Our intact AE1 models allow, for the first time to our knowledge, to identify which are the interacting residues between the cdAE1 and mdAE1 (Fig. S9 *B*). Our simulations suggest that the mdAE1 cytoplasmic loops that include residues 690–705 (between TM8, 9), 736–760 (between TM10, 11), 801–830 (H4-H5, between TM12, 13), and 873–887 (H6, in the C-terminal tail) form the main direct interactions between the mdAE1 and the cdAE1 in both the *rev-V* and *V* orientations of the cdAE1 (Fig. S9 *B*). The interacting cdAE1 residues are different between the *rev-V* and *V* conformers and are shown in Fig. S9 *B*. Interestingly, in the *rev-V* conformers, the cdAE1 N-terminal residues 55–56 interact strongly with the mdAE1 (Fig. S9 *B*, black and red histograms), but these interactions may be not present in the AE1 kidney isoform, which lacks the first 65 amino acids (72). Moreover, residues 142, 143, and 175–185 in the *V* conformers are buried and therefore not available for interacting with the ankyrin, suggesting once more that the *V* conformers may not be a likely conformation (Fig. S9 *B*, green and blue histograms).

Atomistic simulations

To characterize the interactions between the two cdAE1 and mdAE1 domains in more detail, the first centroid from the cluster analysis shown in Fig. 2 of each CG-MD system was converted to an atomistic representation (see Methods). Calculation of the buried surface area in our models suggests that the twisted conformers have a higher overall buried area compared to the parallel conformers (Fig. S10; Table 1).

The cdAE1/cdAE1 dimer interface has a larger contribution to the buried surface area compared to the mdAE1/mdAE1 dimer (Fig. S10, *B* and *C*; Table 1). The values of the buried surface area from our simulations are in agreement with the structural data of the cdAE1 (24.5, 22.8 nm^2 for PDB: 1HYN (9) and PDB: 4KY9 (10), respectively). Our analysis also shows that the value of the mdAE1/mdAE1 buried surface of the *rev-V* conformers is more similar to the buried surface of the crystal structure of the mdAE1 (10.92 nm^2) (7). Both parallel and twisted *V* conformers show lower values for the buried surface area that deviate more from the experimental buried surface area of the crystal structures. These results indicate that the orientation of the cdAE1 is indeed critical for the stabilization of the AE1 dimer.

To measure the compactness of each conformer, we measured the volume of the cavity that is formed between the membrane and the cytosolic domains of the AE1 after they form a complex (Fig. S2, *A* and *D*). This cavity is almost three times larger in the *V* conformers compared to the *rev-V* models in which the values are also more comparable between the parallel and twisted conformers. This may be because of the fact that in the *V* conformers, the V-shape groove in the cytoplasmic domains faces the mdAE1, creating a cavity between the mdAE1 and the cdAE1 (Fig. S2 *A*). This may suggest that the cdAE1/mdAE1 complex dimer is likely to be less stable in the *V* conformer compared to the *rev-V*. In line with this, the *V* conformers are also less compact compared to the *rev-V* as suggested by the radius of gyration calculated in the atomistic simulations (Fig. S2 *E*).

Calculation of the H-bonds between the two AE1 monomers in the atomistic simulations confirms that most of the interaction interface is located in the cdAE1, with the dimerization arms playing a critical role. In particular, 9 over a total of 11, 8 over 12, 9 over 12, and 12 over 15 H-bonds were found between residues of the cdAE1 in the

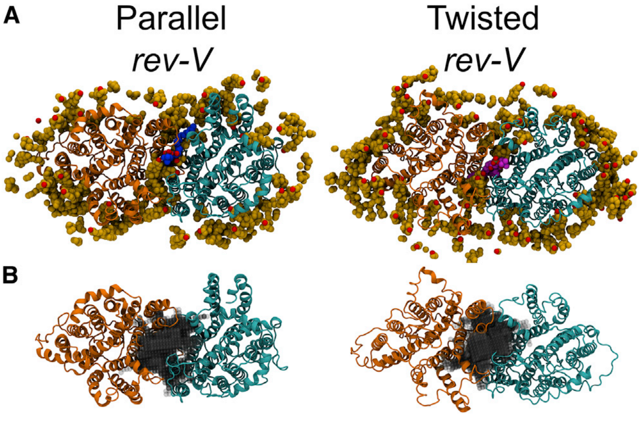

FIGURE 5 Interaction of AE1 with cholesterol. (*A*) Snapshots from the atomistic simulations of each model in the *rev-V* conformation show the interactions of the mdAE1 dimer (*orange* and *cyan ribbon*) with cholesterol (*yellow spheres*). View is from the cytoplasmic side. The cdAE1 was omitted for clarity. Molecules of SM (*blue spheres*) and POPC (*magenta spheres*) are shown. (*B*) The volume in the mdAE1 dimer interface that is filled with lipids is shown in gray. See also Figs. S11 and S12. To see this figure in color, go online.

rev-V parallel, *rev-V* twisted, *V* parallel, and *V* twisted, respectively (Table S2).

Calculation of the RMSF of the protein in the atomistic simulations reveals that in the *V* conformation, the linker region is rather flexible, whereas in the *rev-V*, it is less flexible (Fig. 3 *B*). This is because in the *rev-V* configuration, the linker is positioned between the mdAE1 and the cdAE1 (Fig. 3 *A*). This may also explain why the *V* structures are less compact. Our analysis also shows that the extracellular loop between TM5 and TM6, where most of the blood group antigens reside (6), is one of the most flexible regions across all the conformers (RMSF of 0.5–0.8 nm).

AE1-lipid interactions

Molecular dynamics simulations that contained complex bilayers have been successfully used to examine the interactions of different lipid species with membrane proteins (e.g., transporters and channels) (44,73,74). Our CG-MD simulations were run in a complex lipid bilayer that resembles the native membrane in which AE1 functions (Table S1). In this section, we focus on the interactions of the *rev-V* conformers, in the twisted and the parallel orientations, as we have shown above that they are more in agreement with the available experimental data. It should be noted, however, that there is a significant agreement in the protein-lipid interactions of the mdAE1 between the *rev-V* and the *V* conformers, indicating that apparently, the orientations of cdAE1 does not affect significantly the capacity of the mdAE1 to interact with lipids. Residues found to form lipid contacts in the CG-MD simulations are show in Fig. S11.

Our analyses reveal that the majority of the contacts between AE1 and lipids occurred, as expected, via the mdAE1 rather than the cdAE1, confirming observations we made previously with MD simulations of the mdAE1 (37). Significant interactions with lipids are observed between the loop regions connecting the TM segments as well as the first helical segment of mdAE1 (H1, residues 383–401) that lies parallel to the inner membrane surface. The helical segment H1 is preceded by the linker region. Accordingly, the C-terminal of the linker interacts moderately with lipid headgroups, especially cholesterol, 1-palmitoyl-2-oleyl-phosphtidylethanolamine (POPE), 1-palmitoyl-2-oleyl-phosphtidylserine (POPS), and phosphatidylinositol 4,5-bisphosphate (PIP_2) (Fig. S11). The residues in the linker region from the *rev-V* conformers that have the highest number of interactions with cholesterol, POPE, POPS, and PIP_2 lipids are residues 377–380 at the conserved C-terminal end of the linker, immediately proximal to H1.

AE1 interactions with cholesterol

In our CG-MD simulations, AE1 interacts strongly with cholesterol as previously suggested in MD studies of the mdAE1 (37) (Fig. S11). The cavity in the mdAE1 dimer interface that is formed by TM6 and TM12 (residues 570–594 and 785–809, respectively) is filled with cholesterol in all the conformers in all simulations. Moreover, during the CG-MD simulations, this cavity between the TM domains was also occupied by 1-palmitoyl-2-oleyl-phosphtidylcholine (POPC) or SM that were in the outer leaflet before entering the AE1 cavity (Fig. 5 *A*). Importantly, the TM6 has a sequence—VL^{577}MAGTFF^{583}FAMMLR^{589}K—that resembles a CRAC motif (75), although it has a Phe in position 583 instead of Tyr. Interestingly, in our CG-MD simulations, residue Phe584 made the highest number of interactions with cholesterol in all the conformers and is part of an "aromatic cluster" formed by Phe582, Phe583, and Phe584. Multiple sequence alignment between AE1, AE2, and AE3 in different species suggests that this sequence region is conserved (see Methods).

Conversion of the first centroids of each CG-MD repeat simulations (Fig. 2 *A*) to an atomistic representation allowed us to study in more detail the volume of the cavity in the dimer interface and its evolution (Fig. 5; Fig. S12 *A*). The volume of this cavity in the *rev-V* conformers is somewhat increased with respect to the value calculated from the crystal structure (7228 $Å^3$, see Fig. S12 *A*). Interestingly, the lipids that were already present in the TM cavity stably reside within this cavity during the simulations (Fig. 5 *A*). In particular, one SM lipid, one POPC lipid, and one POPC lipid were found in the atomistic simulations *pa_rev-V_AE1_AT_native*, *tw_rev-V_AE1_AT_native* and *pa_V_AE1_AT_native*, respectively (Fig. 5 *A*; Table S1). Noticeably, residues known to cause human diseases when mutated were also found in close contact with cholesterol (Figs. S11 and S12, *B* and *C*).

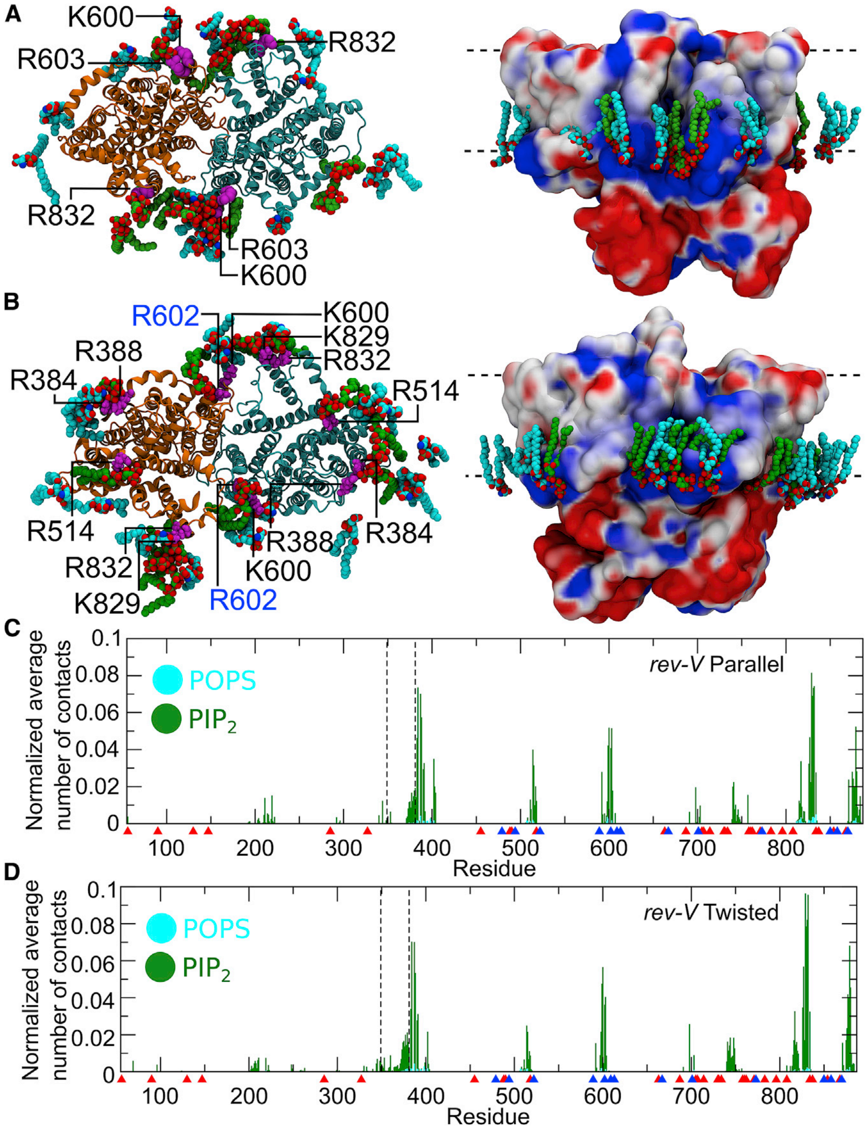

FIGURE 6 Interaction of AE1 with anionic lipids. *rev-V* models in parallel and twisted conformation are shown in (*A* and *B*), respectively. The structures are the first centroid from the cluster analysis from the atomistic simulations in the native lipid mixture. The cdAE1 has been omitted for clarity in the view from the cytoplasmic side on the left. Positively charged residues from each chain involved in H-bonds in both subunits with PIP_2 (*green*) and POPS (*cyan*) are shown in magenta (Table S3). On the right, surface representation of the AE1 dimer (*side view*), colored based on the electrostatic potential in aqueous solution at pH 7, calculated using APBS (57), with positive shown blue, neutral white, and negative red. The residue Arg602 causing AD-dRTA when mutated (76) is indicated in blue. The PIP_2 and POPS lipids from this snapshot that interact with the protein are also shown. Plots in (*C* and *D*) show the normalized average number of contacts (within a cut-off distance of 0.55 nm) between AE1 and the headgroups of PIP_2 (*green*) and POPS (*cyan*) lipids in the bilayer (across all CG-MD repeat simulations). For the normalization, the number of contacts of a residue with a lipid type was divided by the number of lipids and the number of frames. See also Fig. S11 and Table S3. To see this figure in color, go online.

AE1 interactions with anionic lipids

Contact analysis between AE1 and the headgroups of anionic lipids POPS and PIP_2 in the CG-MD simulations of all conformers reveals specific sites of interactions with anionic lipids. These are located in the H1 helix (residues 383–401), cytoplasmic loops between TM4, 5 (residues 506–518), TM6, 7 (residues 593–600), TM12, and 13 (residues 801–830), and the C-terminal region (residues 873–887) (Fig. 6, *C* and *D*).

Calculation of the electrostatic potential of the intact AE1 shows that the sites identified above are positively charged, allowing ionic interactions with the negatively charged phosphate groups of the lipids. Fig. 6 shows conserved basic residues located in these sites and at the interface of the mdAE1 dimer, which are involved in H-bonds interactions with anionic lipids for the *rev-V* conformers. Noticeably, residue Arg602 (Fig. 6), when mutated to histidine, causes recessive distal renal tubular acidosis (76). Although disease-causing mutations may also result in conformational changes within the protein, our results suggest that the positive charge in position 602 may be essential to stabilize the mdAE1 dimer interface. The importance of "interfacial lipids" in membrane protein stability has been also observed for other membrane proteins (77) as well as in the AE1 closely related eukaryotic transporter UapA from *Aspergillus nidulans* (78). Therefore, our results suggest that anionic lipids such as POPS and PIP_2 may play a possible stabilizing role for the AE1 dimer, particularly during folding in the endoplasmic reticulum, and that mutations that disrupt lipid interactions may cause misfolding and trafficking defects in AE1.

Moreover, even though the C-terminal residues in our AE1 models interact with both anionic lipid species used in this study, we noticed a strong preference for the PIP_2 headgroups (Figs. 6, *C* and *D* and S11; Table S3), especially

of the two segments that connect TM12 and TM13 (residues 801–830) and the C-terminal tail (residues 873–887). This behavior is retained in the atomistic simulations in which the same series of positively charged residues in the C-terminal region are involved in H-bonds with PIP_2 (Fig. 6, *C* and *D*; Table S3). The AE1 C-terminal region also interacted strongly with anionic lipids and POPE lipids for both *rev-V* and *V* conformers. Interestingly, residues that interact strongly with lipids in our simulation are also connected to different variants (i.e., R518 (Dresden) (79), G714 (Okinawa) (80), Trp831, Arg832, His834 (variant Birmingham) (81,82), Arg879, and R870 (Prague III variant) (82–84)) (Fig. S11; Table S3).

DISCUSSION

The cd/mdAE1 complex

In this study, we have built models of a near full-length AE1 in a complex lipid bilayer to determine the nature of the interactions of the cdAE1 with the membrane domain and with lipids. Our results demonstrate how possible orientations of the cdAE1 associate with the mdAE1 (Fig. 1). Each model has been further validated against available structural and biochemical data. This comparison suggests that the *rev-V* configuration is more likely, although a *V* arrangement may also be possible because of the long linker between the mdAE1 and the cdAE1 (21).

Several studies showed that in addition to its transport function, AE1 interacts with the cytoskeleton. This interaction is critical for maintaining the red cell membrane integrity (58–61,85,86). To evaluate our models, we mapped known sites of cdAE1 that interact with cytoskeletal proteins (i.e., ankyrin and actin) in our models (Fig. 2). As shown in Fig. 2, most of the interaction sites of cdAE1 and the aforementioned cytoskeletal proteins are found in the V-shape groove formed by the cdAE1 dimeric structure (Fig. 1 *A*), suggesting that this portion of the cdAE1 should be more exposed and accessible from the cytoplasmic side. This only occurs in our models in which the cdAE1 is in a *rev-V* conformer (Fig. 1). It should also be noted that the cdAE1 is mostly negatively charged (Fig. 6 *A*) with the exception of a positively charged region in the V-shaped groove that may be important in the recognition of cytoskeletal proteins. Accordingly, previous data showed an *in vitro* pH dependence for the AE1-ankyrin association (24).

Our results suggest that the *rev-V* models have higher compactness and stability compared to the *V* conformers. This has been confirmed by analysis of the radius of gyration and buried surface area of the AE1 dimer. The latter reveals higher values for the *rev-V* conformers that are more in agreement with the ones observed for the AE1 crystal structures (Fig. S10; Table 1). A *V* orientation of the cdAE1 results in a decrease of the mdAE1 interface buried surface (Table 1), demonstrating that the orientation/interaction between the cdAE1 and the mdAE1 affects the packing of the mdAE1. Overall, a *rev-V* configuration retains the structure of the mdAE1 seen in the recent crystal structure. Additionally, our models reveal that the highest buried surface in the dimer interface is located in the cdAE1 rather than the mdAE1. For the first time, we are able to provide an estimate for the surface of interaction between the dimeric cdAE1 and mdAE1 complexes. This estimate is $\sim$21 nm^2.

The model proposed by Rivera-Santiago et al. (23) is comparable with the *V* parallel conformer discussed in this study (Fig. 1 *A*), but this *V* model is not as compact and stable as the *rev-V* conformers (Fig. S1). Additionally, the final RMSD between the Rivera-Santiago AE1 model (23) and the first centroid from our atomistic simulations with the *V* parallel conformer (Fig. 2 *A*) is 19.5 Å.

Role of the linker region in AE1

In this study, we investigated the possible role of the linker region (residues 349–381) that connects the two AE1 main domains and possibly facilitates their interaction. Although the linker is predicted to be disordered and unstructured (residues 332–376), it has been suggested to introduce flexibility to the cytoplasmic domain (21). Our simulations showed that the linker is involved in the interaction between the cdAE1 and the mdAE1 domains, but its removal did not change the stability of the mdAE1/cdAE1 complex after it was formed (Fig. S8). Multiple sequence alignment between members of the SLC4 family (AE1, AE2, and AE3, see Methods) showed that although the N-terminal region (S349-P368) of the linker is poorly conserved, the linker C-terminal region (D369-G381) is rather conserved. Our analyses reveal that the AE1 linker region should not be considered as a mere connector between the two main domains. Its C-terminal conserved region is involved in specific lipid contacts (Figs. 4 and 6, *C* and *D*). Moreover, in our CG-MD simulations, linker residues are involved in the interface between cdAE1 and mdAE1 (Fig. S9) and residues Lys353 and Asp363 were found to form interchain H-bonds in the atomistic simulations (Table S2).

Interaction of AE1 with lipids

The complex bilayer in our simulations allowed us to examine and identify lipid species that form a key interaction with intact AE1 (Fig. S11). Our results show that the pattern of interactions of the mdAE1 with lipids was similar with the different cdAE1 orientations. This may suggest that the two domains function independently (10,12–14). The cdAE1 has only a small number of interactions with POPE and PIP_2 lipids (Fig. S11).

Our simulations revealed that the cavity of the mdAE1 dimer interface is filled in with cholesterol (Fig. 5 *A*) in good agreement with a previous study of the isolated mdAE1 (37). This may suggest a role of cholesterol in

AE1 stability and function. An increase in the level of cholesterol has been linked to a decrease in AE1 anion transport (87–89). This is probably due to an increase in the stiffness of the red blood cell plasma membrane, restricting the conformational change in AE1 associated with transport. Moreover, cholesterol affects the aggregation state of AE1 (90). Our simulations showed that a conserved CRAC-like domain (residues 577–589), which interestingly is situated in the mdAE1 dimer interface, interacts with cholesterol. This region is also characterized by an aromatic cluster of phenylalanines (Phe582, Phe583, and Phe584). Binding of the sterol to this site could affect the relative movement of the gate and core domains that is required for the anion transport, suggesting a regulatory role for cholesterol.

Interestingly, our simulations also showed that the cavity between the gate subdomains is also occupied by a molecule of SM or POPC (in the *rev-V* parallel, *rev-V* twisted, and *V* parallel conformers, respectively) (Fig. 5 *A*). Both of these lipids were in the outer leaflet before entering the AE1 cavity. Interestingly, SM and POPC were shown to compete with cholesterol to negatively (SM) or positively (POPC) alter the anion transport in AE1 (87). The POPC molecule enters the cavity in the interface during the CG-MD simulation run and after the equilibration phase, whereas the SM headgroup migrates within the cavity during the initial equilibration step. Reconstituted AE1 in lipid vesicles indicate that an increased level of SM or POPS negatively affects AE1 transport (91). Higher levels of SM are known to increase the rigidity of the membrane (92,93), and a high concentration of polar negative headgroups may affect the fluidity of the membrane (94), preventing the access of anions to the entrance of the transporter (95).

Despite their relatively low concentration in the inner leaflet (Table S1), PIP_2 molecules were found to interact strongly with AE1 in our simulations (Fig. 6). Similarly, PIP_2 lipids as well as cholesterol were also found to have a possible role in the interfaces for other transporters (44,74,96). In red blood cells, PIP_2 molecules inhibit the binding of protein 4.1 to AE1 and enhance binding to glycophorin C (97,98). As a consequence, the interaction of AE1 with glycophorin C enhances AE1 trafficking from the endoplasmic reticulum to the plasma membrane and stimulates anion transport activity (99,100). A previous computational study using the mdAE1 also suggested that PIP_2 lipids interact preferentially with the protein (37). In our simulations, we observed the intact AE1 interacting with PIP_2 in three main cytoplasmic regions during the CD-MD simulations: 1) the helical segment H1, 2) the region between TM6 and TM7, and 3) the C-terminal tail (Fig. 6). The H1 lies on the surface of the inner leaflet and is the continuation of the linker domain that connects cdAE1 and mdAE1. In some cases, we observe PIP_2 headgroups protruding on top of H1 and toward the linker region.

AE1 mutations in a lipid context

Comparison of our simulations with available experimental data demonstrates that many residues that were shown to affect the function of AE1 when mutated are involved directly in the interactions with lipids, mostly with cholesterol and anionic lipids (Figs. S11 and S12, *B* and *C*). Noticeably, many mutations result in the change of the net charge of the mutated residue, usually the loss of a positive charge (e.g., H834P (variant Birmingham (81,82)), R870W (variant Prague III (82–84)), G714R (variant Okinawa (80)), R518C (variant Dresden (79)) and R602H (76)). Therefore, some of the folding and functional effects that were seen with AE1 mutants may be due to changes of protein/lipid interactions. The lipid interactions may be particularly important as AE1 must fold up properly in a lipid bilayer during its biosynthesis in the endoplasmic reticulum; lipids, in this case, act as membrane protein chaperones (101,102). Our results also suggest that residues 397–404, distal to the helical segment H1 at the beginning of mdAE1, interact strongly with PIP_2 but also with POPE and cholesterol (Fig. S11). These residues are part of a segment that, when deleted (residues 400–408), causes the Southeast Asian ovalocytosis disease (103,104). These interactions are found in the simulations of both conformers, highlighting that some interactions with the lipids occur irrespective of the cdAE1 orientation.

Calculation of the electrostatic potential of the intact AE1 revealed a positively charged patch that is located at the interface between the two mdAE1 chains (Fig. 6, *A* and *B*). This region is comprised of a short segment that connects the TM6 and TM7 (residues 593–600). This segment interacts mainly with PIP_2 (Fig. 6, *C* and *D*) and with some other lipid species present in our native lipid mixture (Fig. S11). A series of conserved positive residues (Arg388, Arg384, Arg514, Lys600, Arg602, Arg603, Lys829, and Arg832) (Fig. 6, *A* and *B*) are involved in H-bonds with anionic lipids in both chains (Table S3), suggesting that these interactions may be important in stabilizing the AE1 dimer. Lipids in similar positions were shown to regulate the homodimerization of other membrane transporters (77,78,105,106), suggesting that the aforementioned lipids may play a similar role in AE1. Point mutations R602H (76), G609R (107), S613F (108), and R589C/H/S (108) in the AE1-kidney isoform that are close to this region have been associated with the AD-dRTA disease. Interestingly, the missense mutation in position 609 retains a normal transport function but the protein is mistargeted to the apical membrane (107), therefore suggesting that this region might be a possible sensor for membrane composition. Moreover, when S613F was expressed in *Xenopus* oocytes, the mutant protein showed a significant chloride transport activity, indicating that the disease is not related simply to the anion transport activity of the mutant protein (108).

Additionally, in our CG-MD and atomistic simulations, we found that anionic lipids and POPE are strongly associated with AE1 C-terminal residues. Interestingly, the AE1 C-terminal segment 812–830 has been defined as the red cell senescent antigen (6,109,110). Accordingly, this region should be available at the extracellular side at the end of the erythrocyte's lifespan toward a presumed conformational rearrangement of the mdAE1 C-terminal tail. One of the main markers of aged erythrocytes is the externalization of the POPS that lead to the naturally occurring accumulation of anti-AE1 antibodies on red blood cell plasma membrane during aging (111,112). It is possible that the interactions of the POPS lipids with the C-terminal region observed in our CG-MD simulations may trigger the exposure to the extracellular surface of this normally cytoplasmic region, but further investigation of this potential mechanism is needed.

CONCLUSIONS

In summary, this study proposes possible models for the near full-length AE1 and reveals critical regions of interactions between cdAE1 and mdAE1 and the role of the linker region. The validity of different conformers has been assessed using available biochemical, functional, and structural data. Our findings suggest that when the cdAE1 is in complex with mdAE1, it is more likely that its V-shaped groove faces the cytoplasm but does not rule out a dynamic interaction that may change the orientation of the two domains. Our results also provide new insights into how the intact AE1 interacts with lipids in its membrane environment. Interestingly, several residues that are linked to diseases and antigens interact strongly with lipids, suggesting that these mutations may change such interactions, resulting in a deficient AE1 folding, stability, and function.

SUPPORTING MATERIAL

Supporting Material can be found online at https://doi.org/10.1016/j.bpj.2019.08.029.

AUTHOR CONTRIBUTIONS

The research was designed by D.D.V., R.A.F.R., and A.C.K.; and D.D.V. performed the research and acquired and analyzed all the data. A.C.K. and R.A.F.R. supervised the project. D.D.V., R.A.F.R., and A.C.K. wrote the article.

ACKNOWLEDGMENTS

D.D.V. and A.C.K. were supported by a Springboard Award from the Academy of Medical Sciences (Great Britain) and the Wellcome Trust (grant number: SBF002\1031). This research was enabled in part by support provided by Scinet (https://www.scinethpc.ca), Compute Canada (www.computecanada.ca), and MARC1, part of the High Performance Computing and Leeds Institute for Data Analytics facilities at the University of Leeds (Leeds, UK).

REFERENCES

1. Reithmeier, R. A. 2001. A membrane metabolon linking carbonic anhydrase with chloride/bicarbonate anion exchangers. *Blood Cells Mol. Dis.* 27:85–89.
2. Sterling, D., R. A. Reithmeier, and J. R. Casey. 2001. A transport metabolon. Functional interaction of carbonic anhydrase II and chloride/bicarbonate exchangers. *J. Biol. Chem.* 276:47886–47894.
3. Lux, S. E., K. M. John, ..., H. F. Lodish. 1989. Cloning and characterization of band 3, the human erythrocyte anion-exchange protein (AE1). *Proc. Natl. Acad. Sci. USA.* 86:9089–9093.
4. Jennings, M. L. 1984. Oligomeric structure and the anion transport function of human erythrocyte band 3 protein. *J. Membr. Biol.* 80:105–117.
5. Casey, J. R., and R. A. Reithmeier. 1991. Analysis of the oligomeric state of Band 3, the anion transport protein of the human erythrocyte membrane, by size exclusion high performance liquid chromatography. Oligomeric stability and origin of heterogeneity. *J. Biol. Chem.* 266:15726–15737.
6. Reithmeier, R. A., J. R. Casey, ..., S. Iwata. 2016. Band 3, the human red cell chloride/bicarbonate anion exchanger (AE1, SLC4A1), in a structural context. *Biochim. Biophys. Acta.* 1858:1507–1532.
7. Arakawa, T., T. Kobayashi-Yurugi, ..., S. Iwata. 2015. Crystal structure of the anion exchanger domain of human erythrocyte band 3. *Science.* 350:680–684.
8. Low, P. S. 1986. Structure and function of the cytoplasmic domain of Band 3: center of erythrocyte membrane-peripheral protein interactions. *Biochim. Biophys. Acta.* 864:145–167.
9. Zhang, D., A. Kiyatkin, ..., P. S. Low. 2000. Crystallographic structure and functional interpretation of the cytoplasmic domain of erythrocyte membrane band 3. *Blood.* 96:2925–2933.
10. Shnitsar, V., J. Li, ..., R. A. Reithmeier. 2013. A substrate access tunnel in the cytosolic domain is not an essential feature of the solute carrier 4 (SLC4) family of bicarbonate transporters. *J. Biol. Chem.* 288:33848–33860.
11. Appell, K. C., and P. S. Low. 1982. Evaluation of structural interdependence of membrane-spanning and cytoplasmic domains of band 3. *Biochemistry.* 21:2151–2157.
12. Lepke, S., and H. Passow. 1976. Effects of incorporated trypsin on anion exchange and membrane proteins in human red blood cell ghosts. *Biochim. Biophys. Acta.* 455:353–370.
13. Grinstein, S., S. Ship, and A. Rothstein. 1978. Anion transport in relation to proteolytic dissection of band 3 protein. *Biochim. Biophys. Acta.* 507:294–304.
14. Rice, W. R., and T. L. Steck. 1977. Pyruvate transport into inside-out vesicles isolated from human erythrocyte membranes. *Biochim. Biophys. Acta.* 468:305–317.
15. Salhany, J. M., K. A. Cordes, and E. D. Gaines. 1980. Light-scattering measurements of hemoglobin binding to the erythrocyte membrane. Evidence for transmembrane effects related to a disulfonic stilbene binding to band 3. *Biochemistry.* 19:1447–1454.
16. Hsu, L., and M. Morrison. 1983. The interaction of human erythrocyte Band 3 with cytoskeletal components. *Arch. Biochem. Biophys.* 227:31–38.
17. Macara, I. G., S. Kuo, and L. C. Cantley. 1983. Evidence that inhibitors of anion exchange induce a transmembrane conformational change in band 3. *J. Biol. Chem.* 258:1785–1792.
18. Yannoukakos, D., C. Vasseur, ..., E. Bursaux. 1991. Human erythrocyte band 3 polymorphism (band 3 Memphis): characterization of the structural modification (Lys 56——Glu) by protein chemistry methods. *Blood.* 78:1117–1120.
19. Ideguchi, H., K. Okubo, ..., N. Hamasaki. 1992. Band 3-Memphis is associated with a lower transport rate of phosphoenolpyruvate. *Br. J. Haematol.* 82:122–125.
20. Jarolim, P., J. Palek, ..., C. M. Cohen. 1992. Band 3 Tuscaloosa: Pro327——Arg327 substitution in the cytoplasmic domain of

erythrocyte band 3 protein associated with spherocytic hemolytic anemia and partial deficiency of protein 4.2. *Blood.* 80:523–529.

21. Jiang, J., N. Magilnick, …, I. Kurtz. 2013. Single particle electron microscopy analysis of the bovine anion exchanger 1 reveals a flexible linker connecting the cytoplasmic and membrane domains. *PLoS One.* 8:e55408.
22. Blackman, S. M., E. J. Hustedt, …, A. H. Beth. 2001. Flexibility of the cytoplasmic domain of the anion exchange protein, band 3, in human erythrocytes. *Biophys. J.* 81:3363–3376.
23. Rivera-Santiago, R., S. L. Harper, …, D. W. Speicher. 2017. Full-length anion exchanger 1 structure and interactions with Ankyrin-1 determined by zero length crosslinking of erythrocyte membranes. *Structure.* 25:132–145.
24. Thevenin, B. J., and P. S. Low. 1990. Kinetics and regulation of the ankyrin-band 3 interaction of the human red blood cell membrane. *J. Biol. Chem.* 265:16166–16172.
25. Van Dort, H. M., R. Moriyama, and P. S. Low. 1998. Effect of band 3 subunit equilibrium on the kinetics and affinity of ankyrin binding to erythrocyte membrane vesicles. *J. Biol. Chem.* 273:14819–14826.
26. Smith, B. D., and T. N. Lambert. 2003. Molecular ferries: membrane carriers that promote phospholipid flip-flop and chloride transport. *Chem. Commun. (Camb.).* 21:2261–2268.
27. Leidl, K., G. Liebisch, …, G. Schmitz. 2008. Mass spectrometric analysis of lipid species of human circulating blood cells. *Biochim. Biophys. Acta.* 1781:655–664.
28. Boutet, E., D. Lieberherr, …, I. Xenarios. 2016. UniProtKB/Swiss-Prot, the manually annotated section of the UniProt KnowledgeBase: how to use the entry view. *Methods Mol. Biol.* 1374:23–54.
29. Jones, D. T., and D. Cozzetto. 2015. DISOPRED3: precise disordered region predictions with annotated protein-binding activity. *Bioinformatics.* 31:857–863.
30. Wei, Y., J. Thompson, and C. A. Floudas. 2012. CONCORD: a consensus method for protein secondary structure prediction via mixed integer linear optimization. *Proc. R. Soc. A. Math. Phys. Eng. Sci.* 468:831–850.
31. Jones, D. T. 1999. Protein secondary structure prediction based on position-specific scoring matrices. *J. Mol. Biol.* 292:195–202.
32. Yan, R., D. Xu, …, Y. Zhang. 2013. A comparative assessment and analysis of 20 representative sequence alignment methods for protein structure prediction. *Sci. Rep.* 3:2619.
33. Lee, B. S., R. B. Gunn, and R. R. Kopito. 1991. Functional differences among nonerythroid anion exchangers expressed in a transfected human cell line. *J. Biol. Chem.* 266:11448–11454.
34. Alper, S. L. 2006. Molecular physiology of SLC4 anion exchangers. *Exp. Physiol.* 91:153–161.
35. Alper, S. L. 2009. Molecular physiology and genetics of Na+-independent SLC4 anion exchangers. *J. Exp. Biol.* 212:1672–1683.
36. Notredame, C., D. G. Higgins, and J. Heringa. 2000. T-Coffee: a novel method for fast and accurate multiple sequence alignment. *J. Mol. Biol.* 302:205–217.
37. Kalli, A. C., and R. A. F. Reithmeier. 2018. Interaction of the human erythrocyte Band 3 anion exchanger 1 (AE1, SLC4A1) with lipids and glycophorin A: molecular organization of the Wright (Wr) blood group antigen. *PLoS Comput. Biol.* 14:e1006284.
38. Šali, A., and T. L. Blundell. 1993. Comparative protein modelling by satisfaction of spatial restraints. *J. Mol. Biol.* 234:779–815.
39. Fiser, A., and A. Šali. 2003. Modeller: generation and refinement of homology-based protein structure models. *Methods Enzymol.* 374:461–491.
40. Shen, M. Y., and A. Sali. 2006. Statistical potential for assessment and prediction of protein structures. *Protein Sci.* 15:2507–2524.
41. Van Der Spoel, D., E. Lindahl, …, H. J. Berendsen. 2005. GROMACS: fast, flexible, and free. *J. Comput. Chem.* 26:1701–1718.
42. Marrink, S. J., H. J. Risselada, …, A. H. de Vries. 2007. The MARTINI force field: coarse grained model for biomolecular simulations. *J. Phys. Chem. B.* 111:7812–7824.
43. de Jong, D. H., G. Singh, …, S. J. Marrink. 2013. Improved parameters for the martini coarse-grained protein force field. *J. Chem. Theory Comput.* 9:687–697.
44. Marrink, S. J., V. Corradi, …, M. S. P. Sansom. 2019. Computational modeling of realistic cell membranes. *Chem. Rev.* 119:6184–6226.
45. Wassenaar, T. A., H. I. Ingólfsson, …, S. J. Marrink. 2015. Computational lipidomics with *insane*: a versatile tool for generating custom membranes for molecular simulations. *J. Chem. Theory Comput.* 11:2144–2155.
46. Bussi, G., D. Donadio, and M. Parrinello. 2007. Canonical sampling through velocity rescaling. *J. Chem. Phys.* 126:014101.
47. Berendsen, H. J. C., J. P. M. Postma, …, J. R. Haak. 1984. Molecular dynamics with coupling to an external bath. *J. Chem. Phys.* 81:3684–3690.
48. Wassenaar, T. A., K. Pluhackova, …, D. P. Tieleman. 2014. Going backward: a flexible geometric approach to reverse transformation from coarse grained to atomistic models. *J. Chem. Theory Comput.* 10:676–690.
49. Lee, S., A. Tran, …, J. B. Klauda. 2014. CHARMM36 united atom chain model for lipids and surfactants. *J. Phys. Chem. B.* 118:547–556.
50. Parrinello, M., and A. Rahman. 1981. Polymorphic transitions in single crystals: a new molecular dynamics method. *J. Appl. Phys.* 52:7182–7190.
51. Darden, T., D. York, and L. Pedersen. 1993. Particle mesh Ewald: an N·log(N) method for Ewald sums in large systems. *J. Chem. Phys.* 98:10089–10092.
52. Hess, B., H. Bekker, …, J. G. E. M. Fraaije. 1997. LINCS: a linear constraint solver for molecular simulations. *J. Comput. Chem.* 18:1463–1472.
53. Humphrey, W., A. Dalke, and K. Schulten. 1996. VMD: visual molecular dynamics. *J. Mol. Graph.* 14:33–38, 27–28.
54. Pettersen, E. F., T. D. Goddard, …, T. E. Ferrin. 2004. UCSF Chimera–a visualization system for exploratory research and analysis. *J. Comput. Chem.* 25:1605–1612.
55. Daura, X., K. Gademann, …, A. E. Mark. 1999. Peptide folding: when simulation meets experiment. *Angew. Chem. Int. Ed.* 38:236–240.
56. Paramo, T., A. East, …, P. J. Bond. 2014. Efficient characterization of protein cavities within molecular simulation trajectories: trj_cavity. *J. Chem. Theory Comput.* 10:2151–2164.
57. Baker, N. A., D. Sept, …, J. A. McCammon. 2001. Electrostatics of nanosystems: application to microtubules and the ribosome. *Proc. Natl. Acad. Sci. USA.* 98:10037–10041.
58. Anong, W. A., T. Franco, …, P. S. Low. 2009. Adducin forms a bridge between the erythrocyte membrane and its cytoskeleton and regulates membrane cohesion. *Blood.* 114:1904–1912.
59. Chang, S. H., and P. S. Low. 2003. Identification of a critical ankyrin-binding loop on the cytoplasmic domain of erythrocyte membrane band 3 by crystal structure analysis and site-directed mutagenesis. *J. Biol. Chem.* 278:6879–6884.
60. Grey, J. L., G. C. Kodippili, …, P. S. Low. 2012. Identification of contact sites between ankyrin and band 3 in the human erythrocyte membrane. *Biochemistry.* 51:6838–6846.
61. Lux, S. E., IV 2016. Anatomy of the red cell membrane skeleton: unanswered questions. *Blood.* 127:187–199.
62. Moura Lima, P. R., T. S. Salles, …, S. T. Saad. 2003. α-cardiac actin (ACTC) binds to the band 3 (AE1) cardiac isoform. *J. Cell. Biochem.* 89:1215–1221.
63. Bustos, S. P., and R. A. Reithmeier. 2011. Protein 4.2 interaction with hereditary spherocytosis mutants of the cytoplasmic domain of human anion exchanger 1. *Biochem. J.* 433:313–322.

64. Bruce, L. J., H. C. Robinson, …, G. W. Stewart. 2005. Monovalent cation leaks in human red cells caused by single amino-acid substitutions in the transport domain of the band 3 chloride-bicarbonate exchanger, AE1. *Nat. Genet.* 37:1258–1263.

65. Bruce, L. J., D. J. Anstee, …, M. J. Tanner. 1994. Band 3 Memphis variant II. Altered stilbene disulfonate binding and the Diego (Dia) blood group antigen are associated with the human erythrocyte band 3 mutation Pro854–>Leu. *J. Biol. Chem.* 269:16155–16158.

66. Steck, T. L., J. J. Koziarz, …, H. Köhler. 1978. Preparation and analysis of seven major, topographically defined fragments of band 3, the predominant transmembrane polypeptide of human erythrocyte membranes. *Biochemistry.* 17:1216–1222.

67. Steck, T. L., B. Ramos, and E. Strapazon. 1976. Proteolytic dissection of band 3, the predominant transmembrane polypeptide of the human erythrocyte membrane. *Biochemistry.* 15:1153–1161.

68. Bustos, S. P., and R. A. Reithmeier. 2006. Structure and stability of hereditary spherocytosis mutants of the cytosolic domain of the erythrocyte anion exchanger 1 protein. *Biochemistry.* 45:1026–1034.

69. Tanphaichitr, V. S., A. Sumboonnanonda, …, S. L. Alper. 1998. Novel AE1 mutations in recessive distal renal tubular acidosis. Loss-of-function is rescued by glycophorin A. *J. Clin. Invest.* 102:2173–2179.

70. Bruce, L. J., O. Wrong, …, M. J. Tanner. 2000. Band 3 mutations, renal tubular acidosis and South-East Asian ovalocytosis in Malaysia and Papua New Guinea: loss of up to 95% band 3 transport in red cells. *Biochem. J.* 350:41–51.

71. Ungsupravate, D., N. Sawasdee, …, P. T. Yenchitsomanus. 2010. Impaired trafficking and intracellular retention of mutant kidney anion exchanger 1 proteins (G701D and A858D) associated with distal renal tubular acidosis. *Mol. Membr. Biol.* 27:92–103.

72. Richards, S. M., M. E. Jaconi, …, M. Pucéat. 1999. A spliced variant of AE1 gene encodes a truncated form of Band 3 in heart: the predominant anion exchanger in ventricular myocytes. *J. Cell Sci.* 112:1519–1528.

73. Friedman, R., S. Khalid, …, C. Domene. 2018. Understanding conformational dynamics of complex lipid mixtures relevant to biology. *J. Membr. Biol.* 251:609–631.

74. Corradi, V., B. I. Sejdiu, …, D. P. Tieleman. 2019. Emerging diversity in lipid-protein interactions. *Chem. Rev.* 119:5775–5848.

75. Fantini, J., and F. J. Barrantes. 2013. How cholesterol interacts with membrane proteins: an exploration of cholesterol-binding sites including CRAC, CARC, and tilted domains. *Front. Physiol.* 4:31.

76. Sritippayawan, S., A. Sumboonnanonda, …, P. T. Yenchitsomanus. 2004. Novel compound heterozygous SLC4A1 mutations in Thai patients with autosomal recessive distal renal tubular acidosis. *Am. J. Kidney Dis.* 44:64–70.

77. Gupta, K., J. A. C. Donlan, …, C. V. Robinson. 2017. The role of interfacial lipids in stabilizing membrane protein oligomers. *Nature.* 541:421–424.

78. Pyle, E., A. C. Kalli, …, A. Politis. 2018. Structural lipids enable the formation of functional oligomers of the eukaryotic purine symporter UapA. *Cell Chem. Biol.* 25:840–848.e4.

79. Eber, S. W., J. M. Gonzalez, …, S. E. Lux. 1996. Ankyrin-1 mutations are a major cause of dominant and recessive hereditary spherocytosis. *Nat. Genet.* 13:214–218.

80. Yawata, Y., A. Kanzaki, …, S. W. Eber. 2000. Characteristic features of the genotype and phenotype of hereditary spherocytosis in the Japanese population. *Int. J. Hematol.* 71:118–135.

81. Jarolim, P., J. L. Murray, …, J. Palek. 1996. Characterization of 13 novel band 3 gene defects in hereditary spherocytosis with band 3 deficiency. *Blood.* 88:4366–4374.

82. Quilty, J. A., and R. A. Reithmeier. 2000. Trafficking and folding defects in hereditary spherocytosis mutants of the human red cell anion exchanger. *Traffic.* 1:987–998.

83. Jarolim, P., H. L. Rubin, …, J. Palek. 1995. Mutations of conserved arginines in the membrane domain of erythroid band 3 lead to a decrease in membrane-associated band 3 and to the phenotype of hereditary spherocytosis. *Blood.* 85:634–640.

84. Bracher, N. A., C. A. Lyons, …, T. L. Coetzer. 2001. Band 3 Cape Town (E90K) causes severe hereditary spherocytosis in combination with band 3 Prague III. *Br. J. Haematol.* 113:689–693.

85. Korichneva, I., M. Pucéat, …, G. Vassort. 1995. Cl- -HCO3- exchange in developing neonatal rat cardiac cells. Biochemical identification and immunolocalization of band 3-like proteins. *Circ. Res.* 77:556–564.

86. Bruce, L. J., S. M. Ring, …, M. J. Tanner. 1999. South-east asian ovalocytic (SAO) erythrocytes have a cold sensitive cation leak: implications for in vitro studies on stored SAO red cells. *Biochim. Biophys. Acta.* 1416:258–270.

87. Schubert, D., and K. Boss. 1982. Band 3 protein-cholesterol interactions in erythrocyte membranes. Possible role in anion transport and dependency on membrane phospholipid. *FEBS Lett.* 150:4–8.

88. Grunze, M., B. Forst, and B. Deuticke. 1980. Dual effect of membrane cholesterol on simple and mediated transport processes in human erythrocytes. *Biochim. Biophys. Acta.* 600:860–869.

89. Gregg, V. A., and R. A. Reithmeier. 1983. Effect of cholesterol on phosphate uptake by human red blood cells. *FEBS Lett.* 157:159–164.

90. Mühlebach, T., and R. J. Cherry. 1982. Influence of cholesterol on the rotation and self-association of band 3 in the human erythrocyte membrane. *Biochemistry.* 21:4225–4228.

91. Köhne, W., B. Deuticke, and C. W. M. Haest. 1983. Phospholipid dependence of the anion transport system of the human erythrocyte membrane. Studies on reconstituted band 3/lipid vesicles. *Biochim. Biophys. Acta.* 730:139–150.

92. Pascher, I. 1976. Molecular arrangements in sphingolipids. Conformation and hydrogen bonding of ceramide and their implication on membrane stability and permeability. *Biochim. Biophys. Acta.* 455:433–451.

93. Barenholz, Y., and T. E. Thompson. 1980. Sphingomyelins in bilayers and biological membranes. *Biochim. Biophys. Acta.* 604:129–158.

94. Browning, J. L. 1981. Motions and interactions of phospholipid head groups at the membrane surface. 3. Dynamic properties of amine-containing head groups. *Biochemistry.* 20:7144–7151.

95. Nałecz, M. J., J. Zborowski, …, L. Wojtczak. 1980. Effect of phospholipid composition on the surface potential of liposomes and the activity of enzymes incorporated. *Eur. J. Biochem.* 112:75–80.

96. Periole, X., T. Zeppelin, and B. Schiøtt. 2018. Dimer interface of the human serotonin transporter and effect of the membrane composition. *Sci. Rep.* 8:5080.

97. Gascard, P., T. Pawelczyk, …, C. M. Cohen. 1993. The role of inositol phospholipids in the association of band 4.1 with the human erythrocyte membrane. *Eur. J. Biochem.* 211:671–681.

98. An, X., X. Zhang, …, N. Mohandas. 2006. Phosphatidylinositol-4,5-biphosphate (PIP2) differentially regulates the interaction of human erythrocyte protein 4.1 (4.1R) with membrane proteins. *Biochemistry.* 45:5725–5732.

99. Groves, J. D., and M. J. Tanner. 1994. Role of N-glycosylation in the expression of human band 3-mediated anion transport. *Mol. Membr. Biol.* 11:31–38.

100. Groves, J. D., and M. J. Tanner. 1994. The effects of glycophorin A on the expression of the human red cell anion transporter (band 3) in Xenopus oocytes. *J. Membr. Biol.* 140:81–88.

101. Dowhan, W., and M. Bogdanov. 2009. Lipid-dependent membrane protein topogenesis. *Annu. Rev. Biochem.* 78:515–540.

102. Mitchell, D. C. 2012. Progress in understanding the role of lipids in membrane protein folding. *Biochim. Biophys. Acta.* 1818:951–956.

103. Jarolim, P., J. Palek, …, S. C. Liu. 1991. Deletion in erythrocyte band 3 gene in malaria-resistant Southeast Asian ovalocytosis. *Proc. Natl. Acad. Sci. USA.* 88:11022–11026.

104. Schofield, A. E., M. J. Tanner, …, W. B. Gratzer. 1992. Basis of unique red cell membrane properties in hereditary ovalocytosis. *J. Mol. Biol.* 223:949–958.

105. Lourdel, S., T. Grand, …, J. Teulon. 2012. ClC-5 mutations associated with Dent's disease: a major role of the dimer interface. *Pflugers Arch.* 463:247–256.

106. Sun, J., J. R. Bankston, …, N. Zheng. 2014. Crystal structure of the plant dual-affinity nitrate transporter NRT1.1. *Nature.* 507:73–77.

107. Rungroj, N., M. A. Devonald, …, F. E. Karet. 2004. A novel missense mutation in AE1 causing autosomal dominant distal renal tubular acidosis retains normal transport function but is mistargeted in polarized epithelial cells. *J. Biol. Chem.* 279:13833–13838.

108. Bruce, L. J., D. L. Cope, …, M. J. Tanner. 1997. Familial distal renal tubular acidosis is associated with mutations in the red cell anion exchanger (Band 3, AE1) gene. *J. Clin. Invest.* 100:1693–1707.

109. Kay, M. M. 1992. Molecular mapping of human band 3 aging antigenic sites and active amino acids using synthetic peptides. *J. Protein Chem.* 11:595–602.

110. Badior, K. E., and J. R. Casey. 2018. Molecular mechanism for the red blood cell senescence clock. *IUBMB Life.* 70:32–40.

111. Mandal, D., A. Mazumder, …, J. Basu. 2005. Fas-, caspase 8-, and caspase 3-dependent signaling regulates the activity of the aminophospholipid translocase and phosphatidylserine externalization in human erythrocytes. *J. Biol. Chem.* 280:39460–39467.

112. Antonelou, M. H., A. G. Kriebardis, and I. S. Papassideri. 2010. Aging and death signalling in mature red cells: from basic science to transfusion practice. *Blood Transfus.* 8 (*Suppl 3*):s39–s47.

Biophysical Journal, Volume *117*

Supplemental Information

Molecular Simulations of Intact Anion Exchanger 1 Reveal Specific Domain and Lipid Interactions

Dario De Vecchis, Reinhart A.F. Reithmeier, and Antreas C. Kalli

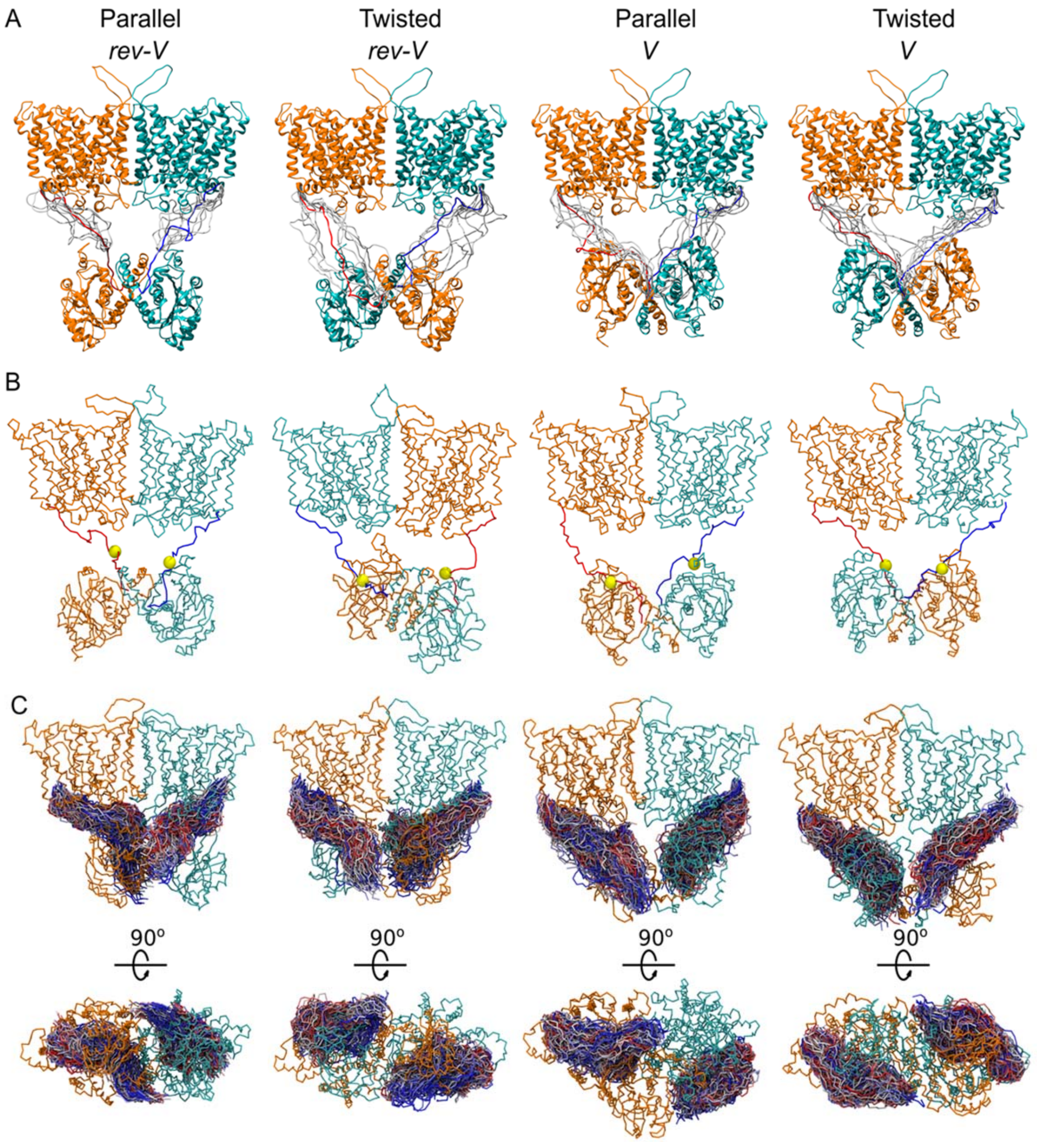

Figure S1. Conformational dynamics of the AE1 linker region. Related to Figures 1, 2 and Methods. (A) Structures are side views of all dimeric AE1 conformations after the modeling stage, showing the relative positions of the mdAE1, the cdAE1, and the linker region. The conformation of the linker of the structures that were used to initiate our simulations are shown in *red* and *blue*. All other conformations of the linker region that were generated during modelling are shown in *grey*. (B) The less sampled conformation for each conformer during the CG-MD simulations. The Lys360 residue is shown as *yellow* sphere. (C) The most sampled conformation for each conformer in the CG-MD simulations. The position of the linker region from all the centroids from all clusters are shown colored from *red* (more populated) to *blue* (less populated). In all panels, the two AE1 subunits are represented in *orange* and *cyan*.

Table S1. Summary of simulations with AE1 in native lipid mixture. Related to Methods.

Simulation	Composition Outer leaflet	Composition Inner leaflet	Duration
Coarse-grained:			
pa_rev-V_AE1-native	POPC:SM:POPE:CHOL (23:22:5:50)	POPC:SM:POPE:CHOL:POPS:PIP_2 (8:4:23:50:13:2)	5 × 5 µs
tw_rev-V_AE1-native	POPC:SM:POPE:CHOL (23:22:5:50)	POPC:SM:POPE:CHOL:POPS:PIP_2 (8:4:23:50:13:2)	5 × 5 µs
pa_V_AE1-native	POPC:SM:POPE:CHOL (23:22:5:50)	POPC:SM:POPE:CHOL:POPS:PIP_2 (8:4:23:50:13:2)	5 × 5 µs
tw_V_AE1-native	POPC:SM:POPE:CHOL (23:22:5:50)	POPC:SM:POPE:CHOL:POPS:PIP_2 (8:4:23:50:13:2)	5 × 5 µs
pa_rev-V_AE1-nolinker	POPC:SM:POPE:CHOL (23:22:5:50)	POPC:SM:POPE:CHOL:POPS:PIP_2 (8:4:23:50:13:2)	5 × 5 µs
tw_rev-V_AE1-nolinker	POPC:SM:POPE:CHOL (23:22:5:50)	POPC:SM:POPE:CHOL:POPS:PIP_2 (8:4:23:50:13:2)	5 × 5 µs
pa_V_AE1-nolinker	POPC:SM:POPE:CHOL (23:22:5:50)	POPC:SM:POPE:CHOL:POPS:PIP_2 (8:4:23:50:13:2)	5 × 5 µs
tw_V_AE1-nolinker	POPC:SM:POPE:CHOL (23:22:5:50)	POPC:SM:POPE:CHOL:POPS:PIP_2 (8:4:23:50:13:2)	5 × 5 µs
Atomistic:			
pa_rev-V_AE1_AT_native	POPC:SM:POPE:CHOL (23:22:5:50)	POPC:SM:POPE:CHOL:POPS:PIP_2 (8:4:23:50:13:2)	3 × 500 ns
tw_rev-V_AE1_AT_native	POPC:SM:POPE:CHOL (23:22:5:50)	POPC:SM:POPE:CHOL:POPS:PIP_2 (8:4:23:50:13:2)	3 × 500 ns
pa_V_AE1_AT_native	POPC:SM:POPE:CHOL (23:22:5:50)	POPC:SM:POPE:CHOL:POPS:PIP_2 (8:4:23:50:13:2)	3 × 500 ns
tw_V_AE1_AT_native	POPC:SM:POPE:CHOL (23:22:5:50)	POPC:SM:POPE:CHOL:POPS:PIP_2 (8:4:23:50:13:2)	3 × 500 ns

Lipid species are: 1-palmitoyl-2-oleyl-phosphtidylcholine (POPC), 1-palmitoyl-2-oleyl-phosphtidylethanolamine (POPE), 1-palmitoyl-2-oleyl-phosphtidylserine (POPS), phosphatidylinositol 4,5-bisphosphate (PIP_2), sphingomyelin (SM) and cholesterol (CHOL).

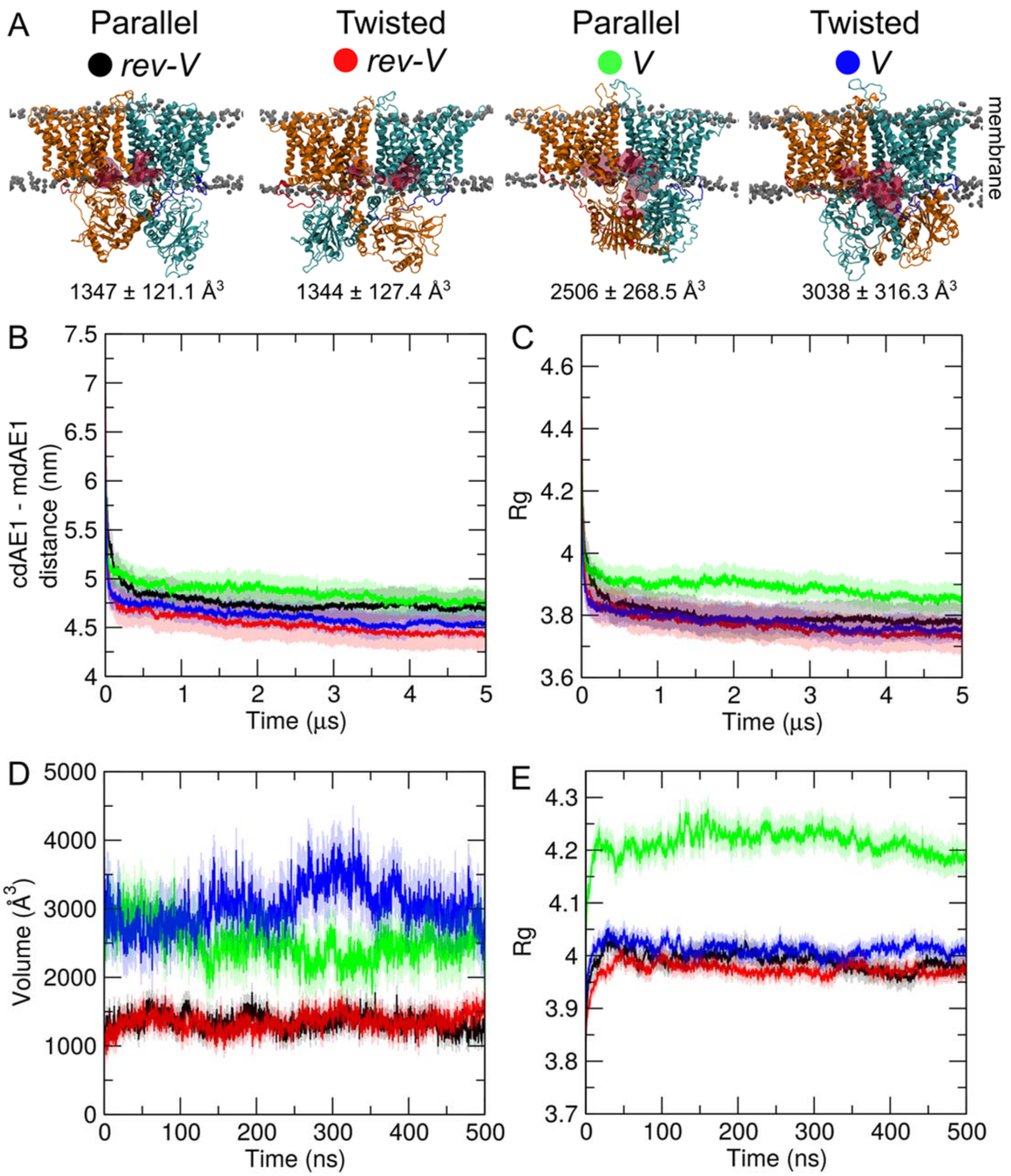

Figure S2. Distance and radius of gyration. Related to Figure 1. (A) Volume of the cavity between the cdAE1 and the mdAE1 dimers. The cavity is shown as a red surface in snapshots from our atomistic simulations. The average value ± standard deviation of the volume is also shown. The AE1 dimer subunits are shown in *orange* and *cyan* ribbon with the linker regions in *red* and *blue*. Phosphate groups from the lipid bilayer are shown as *grey* spheres. (B) Average distance between the cdAE1 and the mdAE1 center of mass during the CG-MD simulations. (C) Radius of gyration of AE1 during the CG-MD simulations. (D) Volume of the cavity shown in A (between the cdAE1 and the mdAE1). (E) Radius of gyration of the AE1 dimer in the atomistic simulations. In all plots the errors are shown and each timeline represents the average over the five repeat CG-MD simulations or the three repeats of the atomistic simulations.

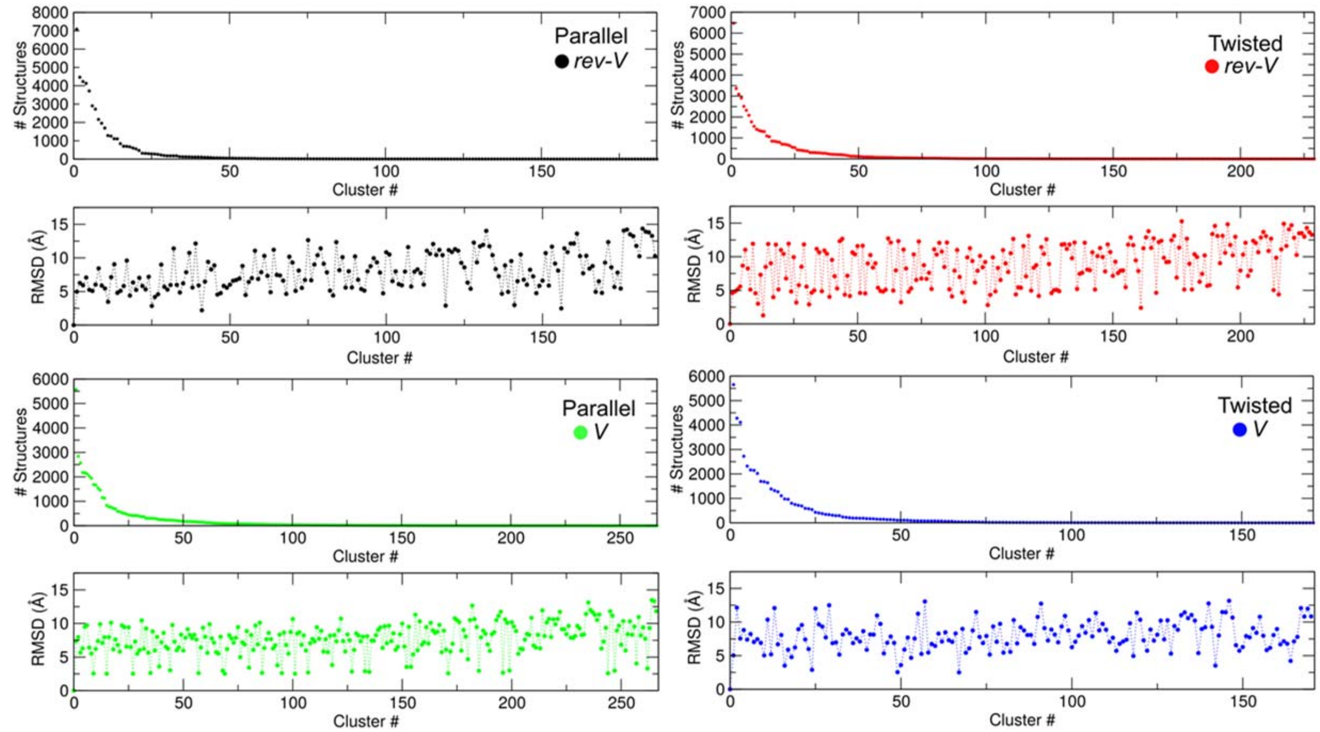

Figure S3. Details of the cluster analysis. Related to Figure 2. For each AE1 conformer (indicated with four different colors) two plots are shown. The plot at the top shows the total number of structures for each cluster. The plots at the bottom show the RMSD variation between the clusters. The structure of the first most populated cluster, has been used as a reference structure to calculate the RMSD of the protein in each cluster.

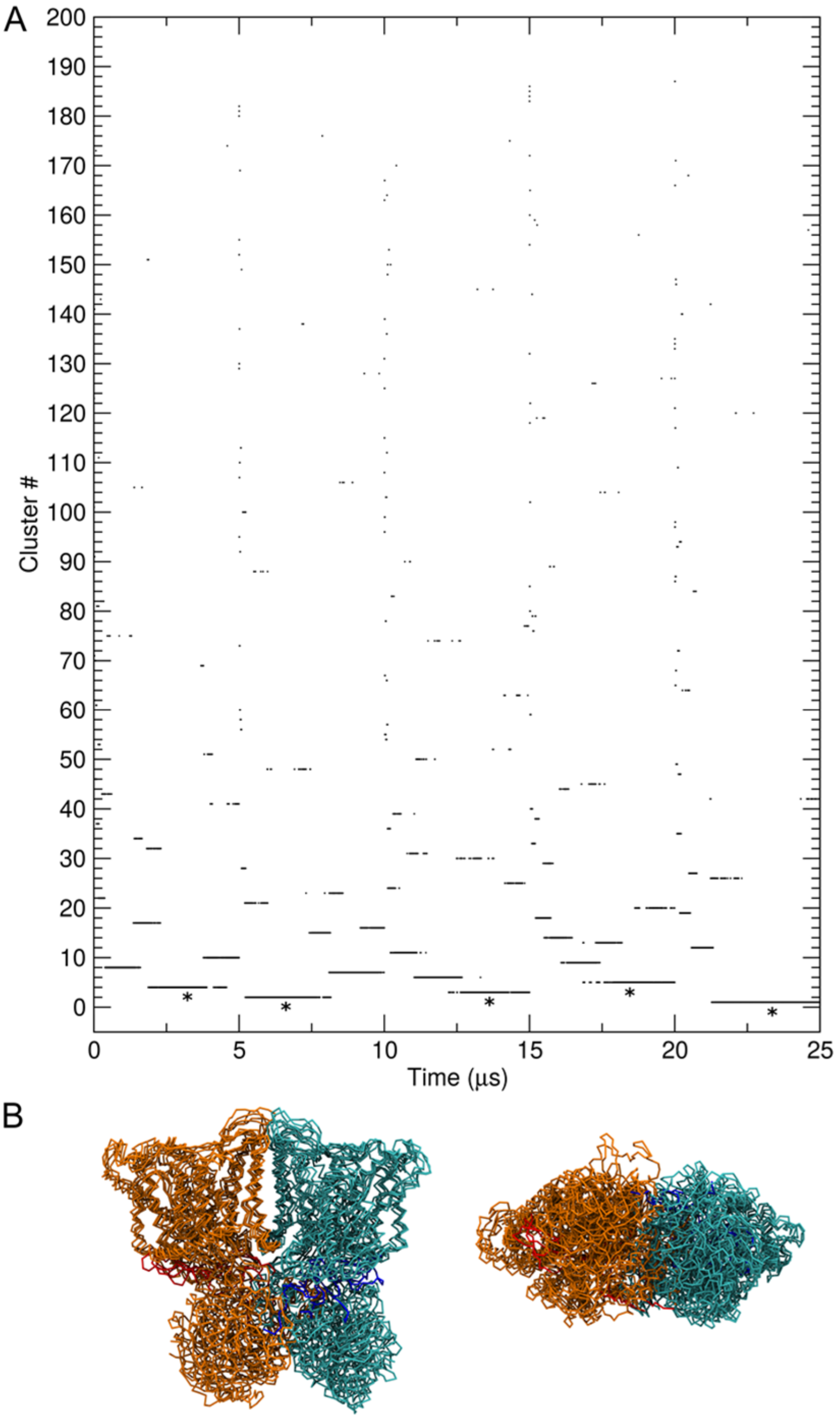

Figure S4. Cluster analysis for the *rev-V parallel* conformer. Related to Figure 2. (A) Occurrence of each cluster as a function of time over the total 25 μs of simulation. The asterisks indicate the first cluster for each repeat CG simulation (every 5 μs). (B) Structures of the centroid of the biggest cluster from each CG-MD repeat simulations (shown using an asterisks). The proteins have been aligned using the backbone atom of the mdAE1. The two AE1 chains are shown in *orange* and *cyan*, with the linker region in *red* and *blue*, respectively.

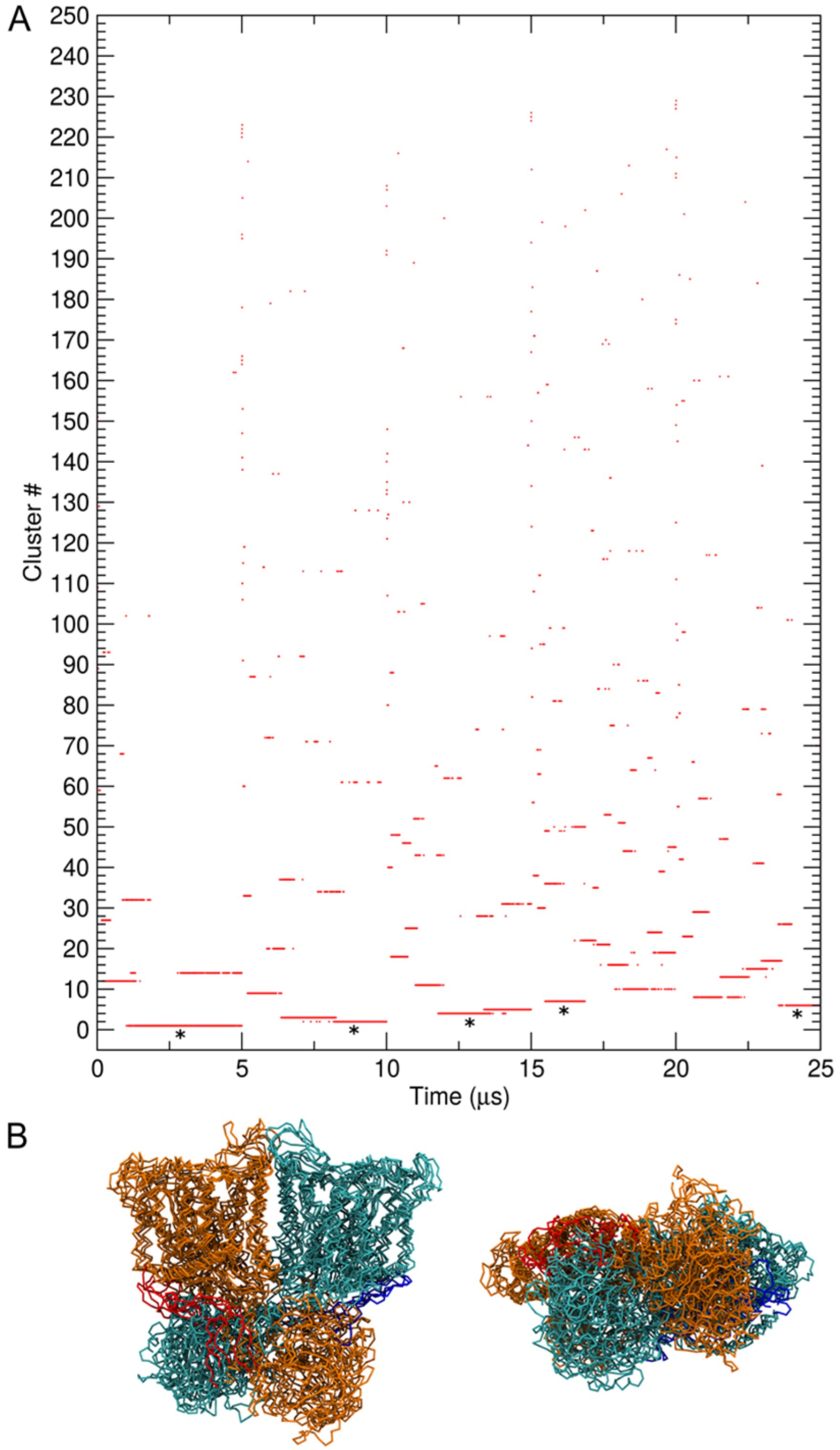

Figure S5. Cluster analysis for the *rev-V Twisted* conformer. Related to Figure 2. A) Occurrence of each cluster as a function of time over the total 25 µs of simulation. The asterisks indicate the first cluster for each repeat CG simulation (every 5 µs). (B) Structures of the centroid of the biggest cluster from each CG-MD repeat simulations (shown using an asterisks). The proteins have been aligned using the backbone atom of the mdAE1. The two AE1 chains are shown in *orange* and *cyan*, with the linker region in *red* and *blue*, respectively.

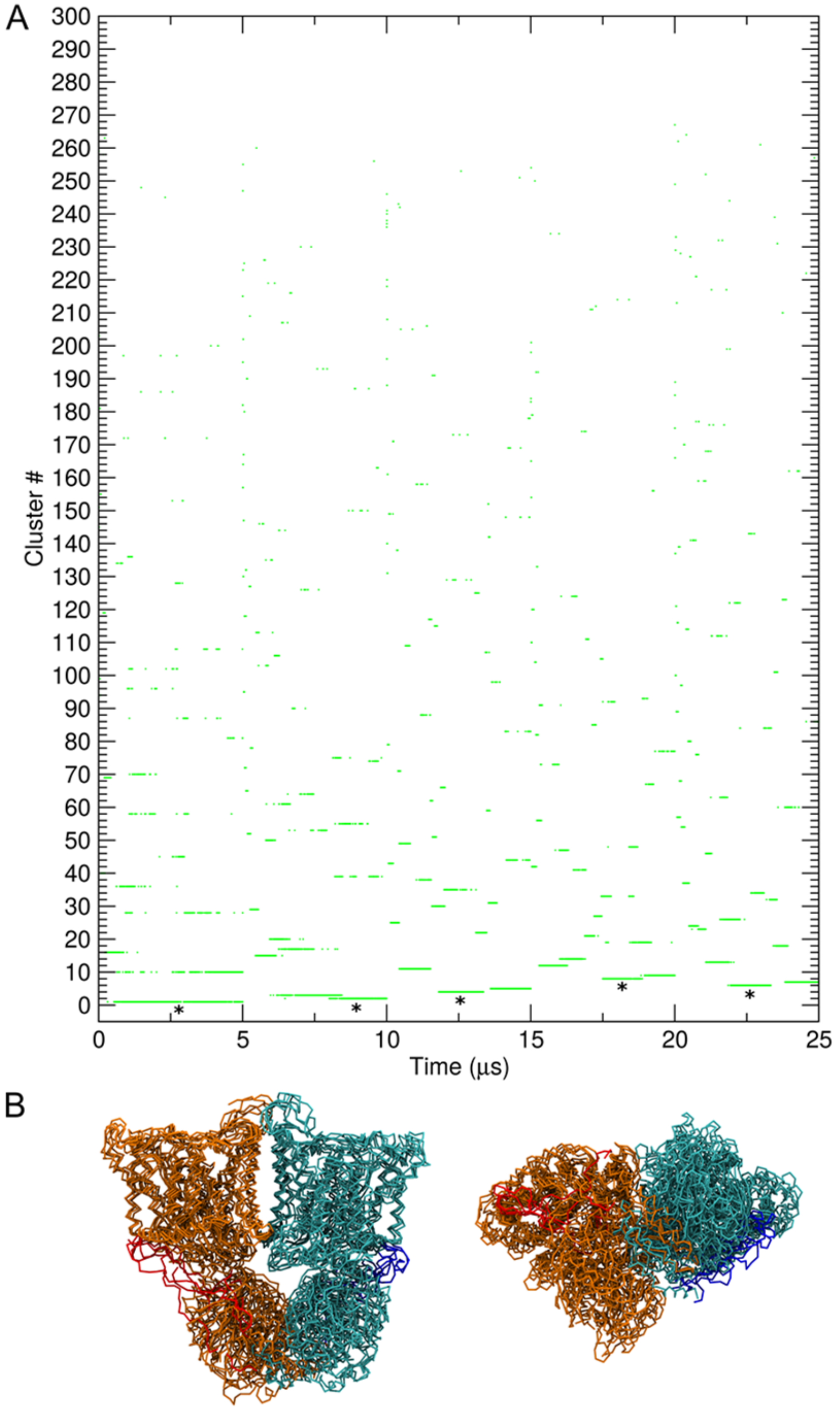

Figure S6. Cluster analysis for the *V Parallel* conformer. Related to Figure 2. A) Occurrence of each cluster as a function of time over the total 25 μs of simulation. The asterisks indicate the first cluster for each repeat CG simulation (every 5 μs). (B) Structures of the centroid of the biggest cluster from each CG-MD repeat simulations (shown using an asterisks). The proteins have been aligned using the backbone atom of the mdAE1. The two AE1 chains are shown in *orange* and *cyan*, with the linker region in *red* and *blue*, respectively.

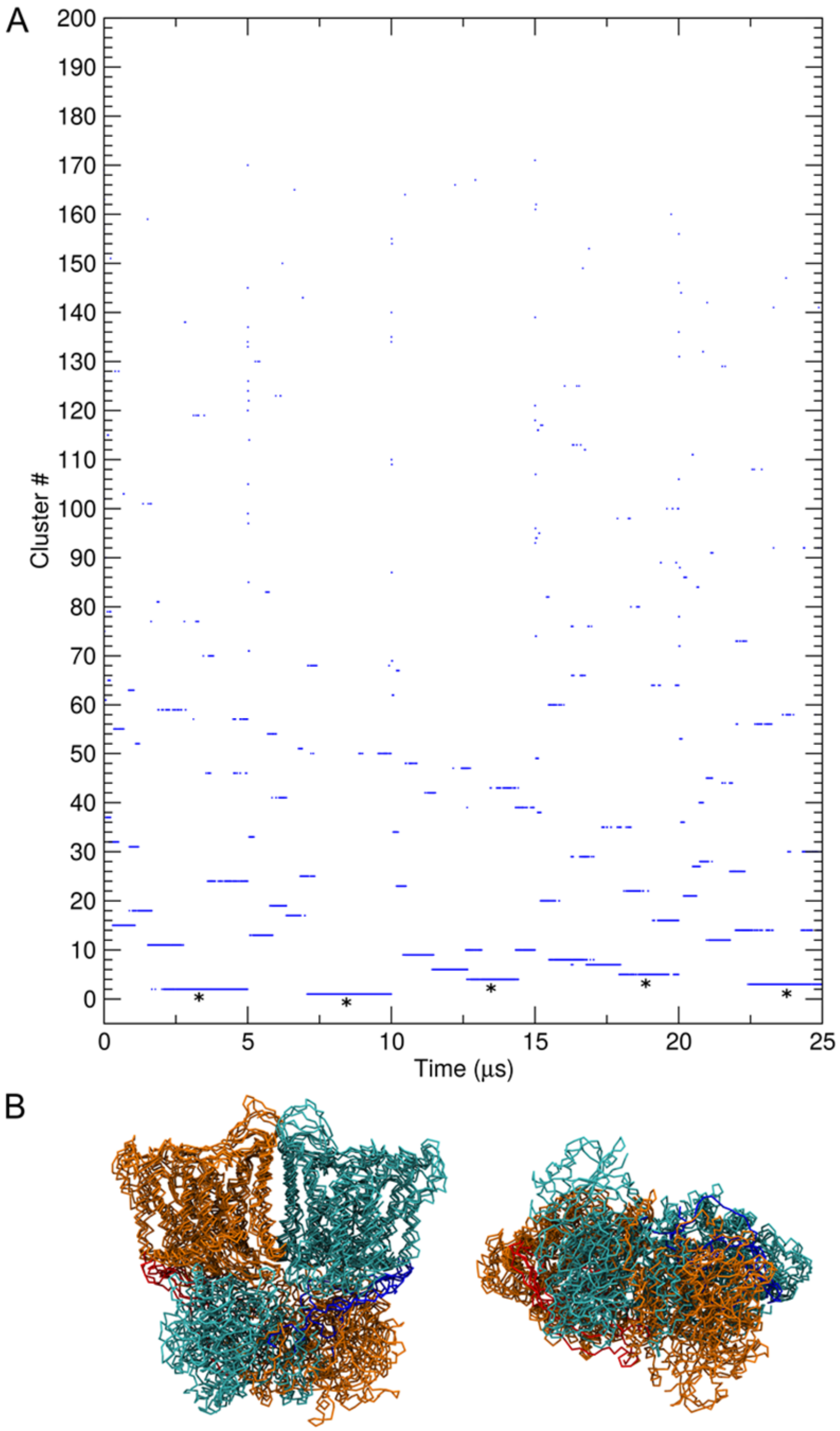

Figure S7. Cluster analysis for the *V Twisted* conformer. Related to Figure 2. A) Occurrence of each cluster as a function of time over the total 25 µs of simulation. The asterisks indicate the first cluster for each repeat CG simulation (every 5 µs). (B) Structures of the centroid of the biggest cluster from each CG-MD repeat simulations (shown using an asterisks). The proteins have been aligned using the backbone atom of the mdAE1. The two AE1 chains are shown in *orange* and *cyan*, with the linker region in *red* and *blue*, respectively.

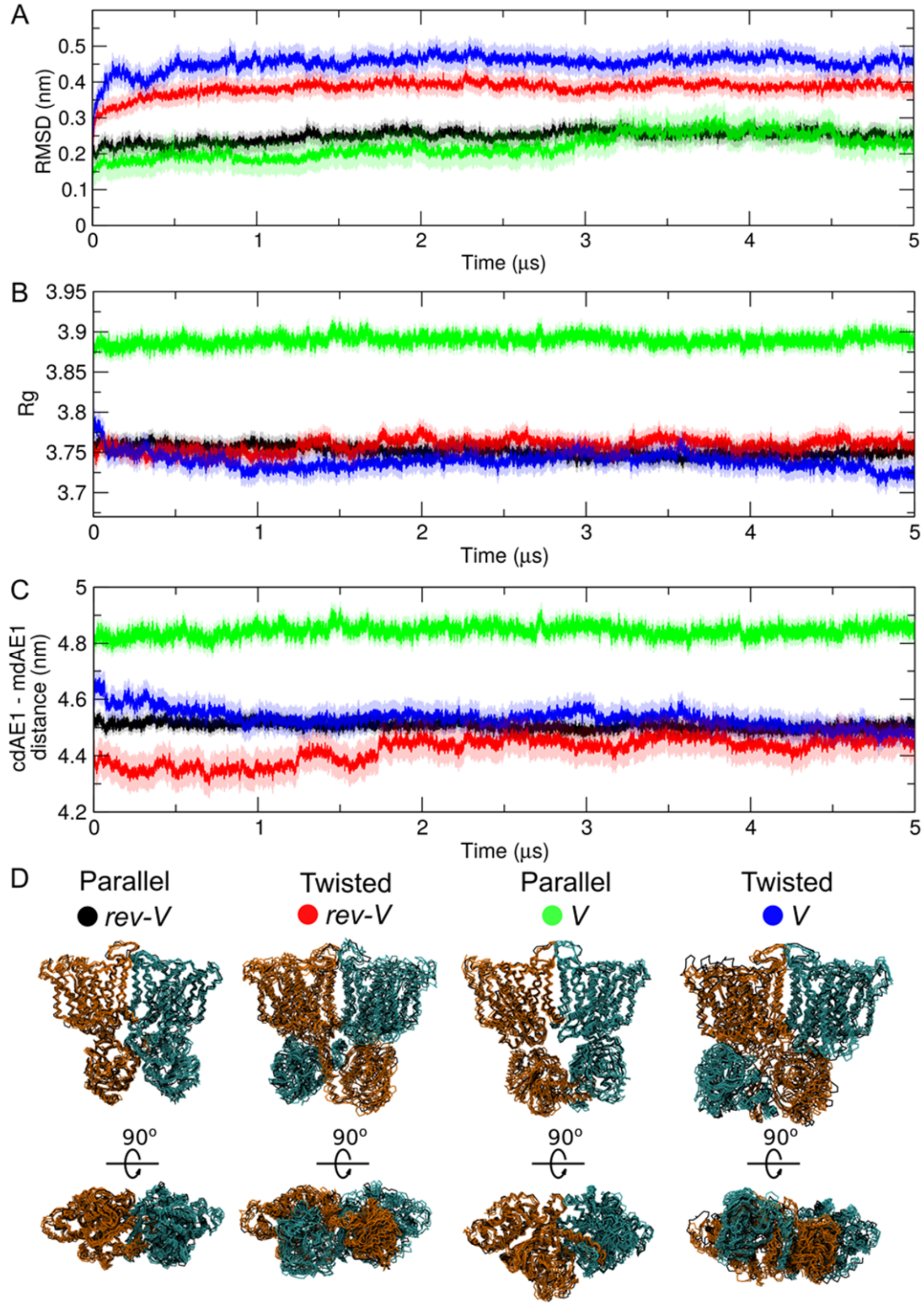

Figure S8 Molecular dynamics simulations without the linker region. Related to Figure 2. (A) RMSD and (B) radius of gyration, both calculated using the backbone particles of the protein. The color for each conformer is shown in D. (C) Distance between the center of mass of the cytoplasmic (cdAE1) and of the transmembrane (mdAE1) domain for each conformer. In all plots the errors are shown and each timeline represents the average over the five repeat CG simulations. (D) Alignment using the backbone particles of the last frame from each of the five repeat simulations and the first selected cluster from which simulations were initiated (in *black*). The two AE1 chains are shown in *orange* and *cyan*.

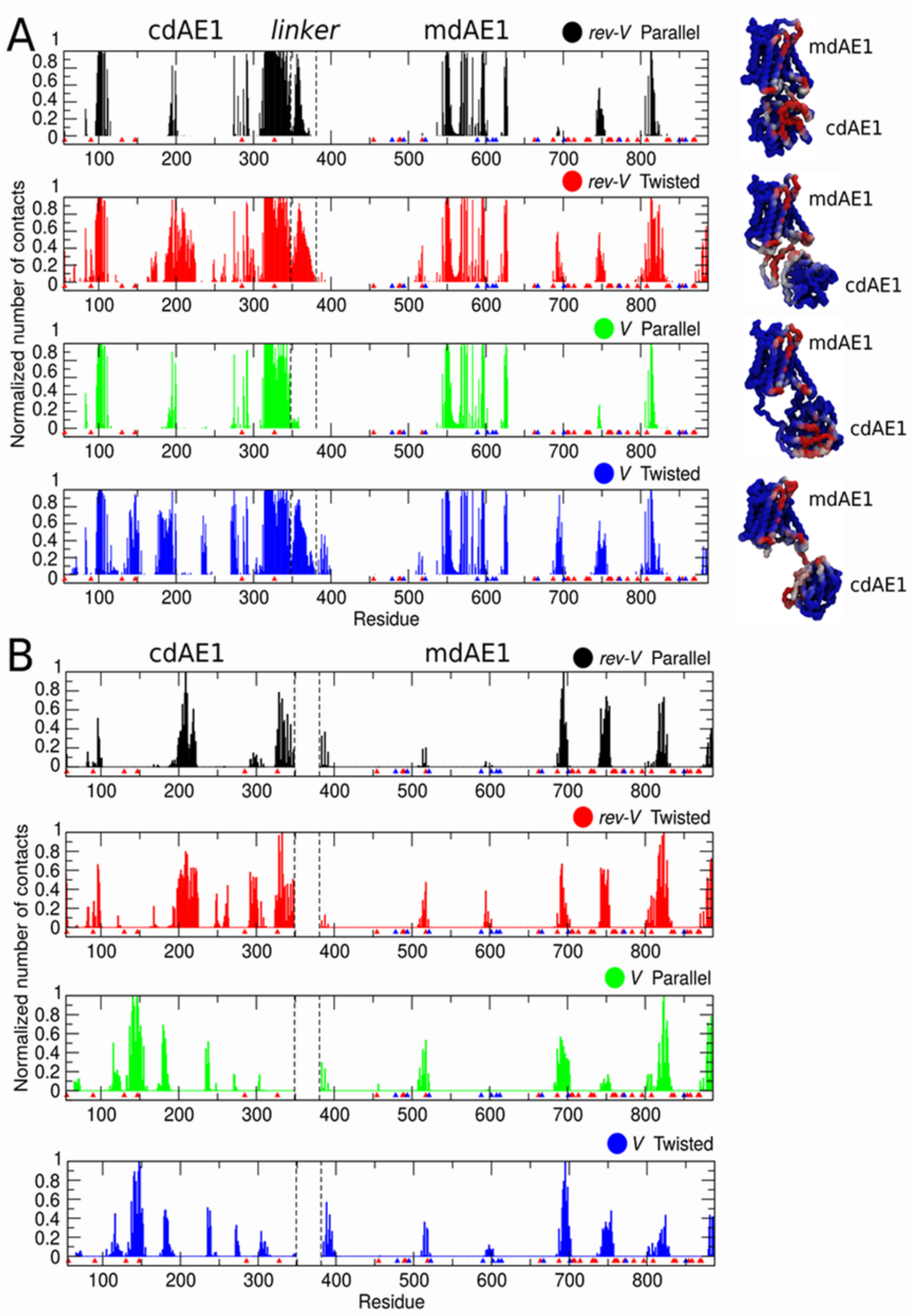

Figure S9. Interactions within the AE1 dimer. Related to Figure 4. Normalized average (over all repeat CG-MD simulations) number of contacts between the two AE1 monomers of the AE1 dimer (A) and between the cdAE1 and the mdAE1 domains (B). Note that for clarity the linker region was not included in the analysis in B and for this reason no contacts are shown between the dotted lines. The contact of the linker region with the mdAE1 and the cdAE1 are shown in the main text. The protein structures in A are snapshots from each conformer in which, the surface of the backbone beads is colored from *blue* (low number of contacts) to *red* (high number of contacts). The mdAE1 is positioned in the same orientation in all structures. In both analyses, the contacts of each AE1 chain were added together. Values close to 0 represents no/low number of contacts whereas 1 represents high number of contacts. A cutoff distance of 0.55 nm was used. Point mutations reported to cause spherocytosis (SPH) or distal renal tubular acidosis (AD-dRTA) are indicated as *red* and *blue* triangles, respectively.

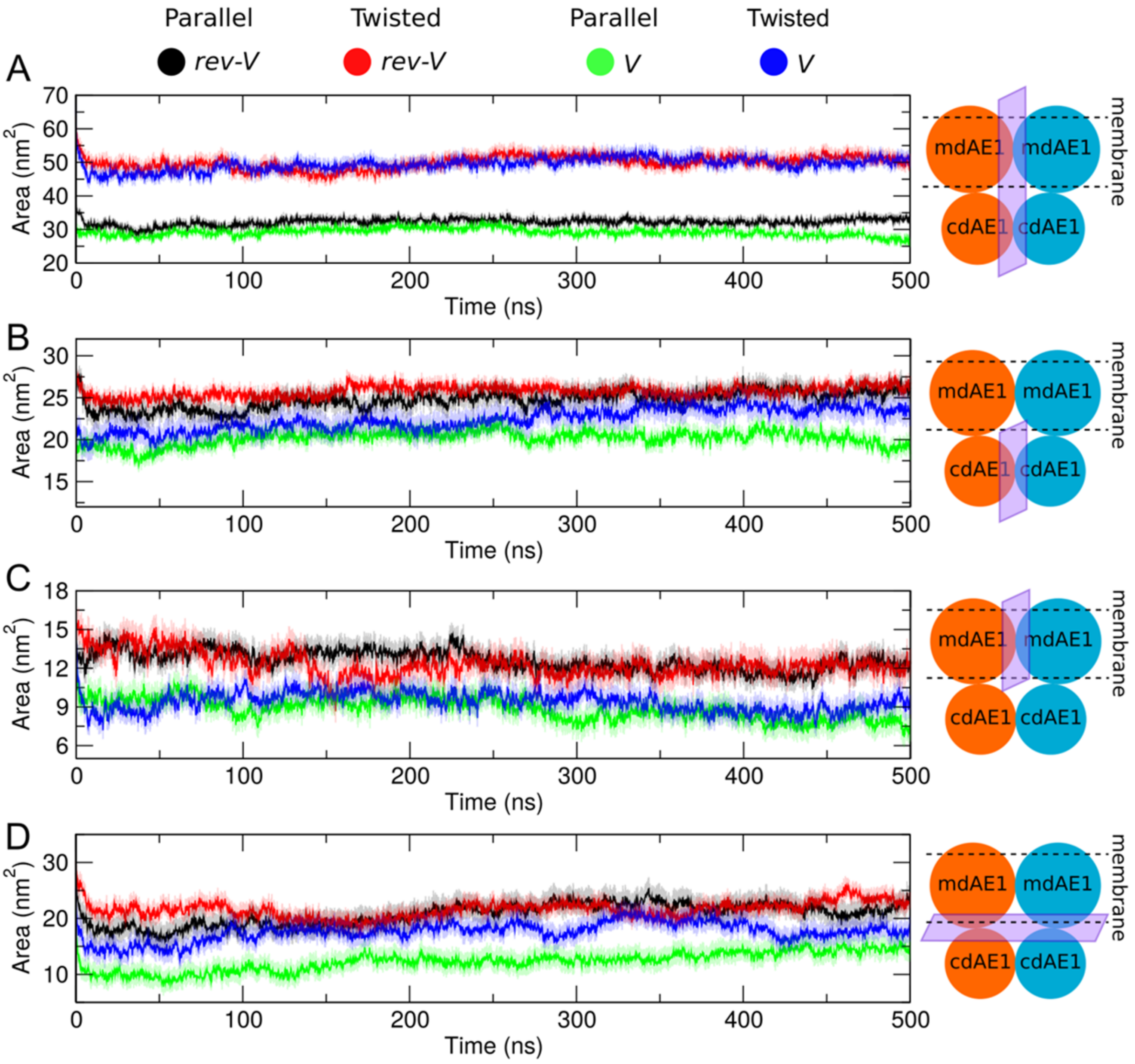

Figure S10. Buried surface area as a function of time for each of the AE1 conformers. Related to Table 1. Buried surface area of the AE1 dimer interface (A), the cdAE1.A/cdAE1.B (B), the mdAE1.A/mdAE1.B (C) and the dimeric cdAE1/mdAE1 (D). The cartoons on the right show the location of the calculated buried areas in a parallel orientation. In all plots the error bars are shown and each timeline represents the average over the five repeat CG simulations.

Table S2. H-bonds between the AE1 dimer in the atomistic simulations. Related to Table 1.

**Parallel** *rev-V*	**Twisted** *rev-V*	**Parallel** *V*	**Twisted** *V*
**Leu321.A – Leu319.B 99.29**	**Leu321.A – Leu319.B 99.07**	**Leu321.B – Leu319.A 99.45**	**Leu321.A – Leu319.B 98.96**
**Leu321.B – Leu319.A 98.16**	**Leu321.B – Leu319.A 95.47**	**Leu321.A – Leu319.B 99.36**	**Leu321.B – Leu319.A 95.46**
**Leu102.B – Val320.A 90.37**	His55.B – Asp887.A 86.15	**Phe597.B – Phe813.A 93.96**	Phe104.A – Ser318.B 87.44
**Leu319.A – Leu321.B 87.38**	Lys691.B – Asp205.A 56.51	Val320.B – Leu534.A 90.71	**Leu319.B – Leu321.A 80.17**
**Val320.A – Leu102.B 78.73**	Arg292.B – Asp887.A 55.62	**Val320.A – Leu102.B 86.95**	**Val320.A – Leu102.B 79.55**
Arg340.B – Aap325.A 54.26	Arg340.B – Glu329.A 55.26	**Leu319.A – Leu321.B 61.93**	Thr324.A – Glu291.B 68.50
**Phe597.A – Phe813.B 52.3**	Lys56.B – Asp887.A 52.31	**Leu319.B – Leu321.A 59.23**	**Val320.B – Leu102.A 63.04**
Arg340.A – Glu329.B 51.79	Val320.A – His101.B 51.56	**Leu102.B – Val320.A 56.52**	Lys691.A – Glu151.B 55.52
**Leu319.B – Leu321.A 48.72**	**Leu102.B – Val320.A 48.74**	Thr324.B – Gln339.A 54.61	**Leu102.B – Val320.A 54.30**
Thr324.B – Glu291.A 44.97	Arg340.A – Glu329.B 43.78	**Trp105.B – Glu312.A 44.97**	Lys691.A – Asp149.B 51.46
Asn569.B – Thr552.A 42.86	**Leu319.B – Leu321.A 43.49**	Phe104.A – Ser318.B 39.87	Lys353.B– Asp277.A 43.96
	Gln191.A – Asp363.B 41.48	Lys551.A – Asp626.B 39.72	Lys698.B – Glu238.A 42.03
			**Trp105.A – Asp316.B 40.34**
			**Trp105.B – Asp316.A 39.88**
			**Leu319.A – Leu321.B 39.70**

Values indicate the percentage of occurrence in the atomistic simulations for each conformer. Residues are donor and acceptor, respectively. Interactions highlighted in bold have been found in the crystal structures PDBs: 1HYN (Zhang et al., 2000), 4KY9 (Shnitsar et al., 2013) and 4YZF (4YZF) using UCSF Chimera with default settings (Pettersen et al., 2004).

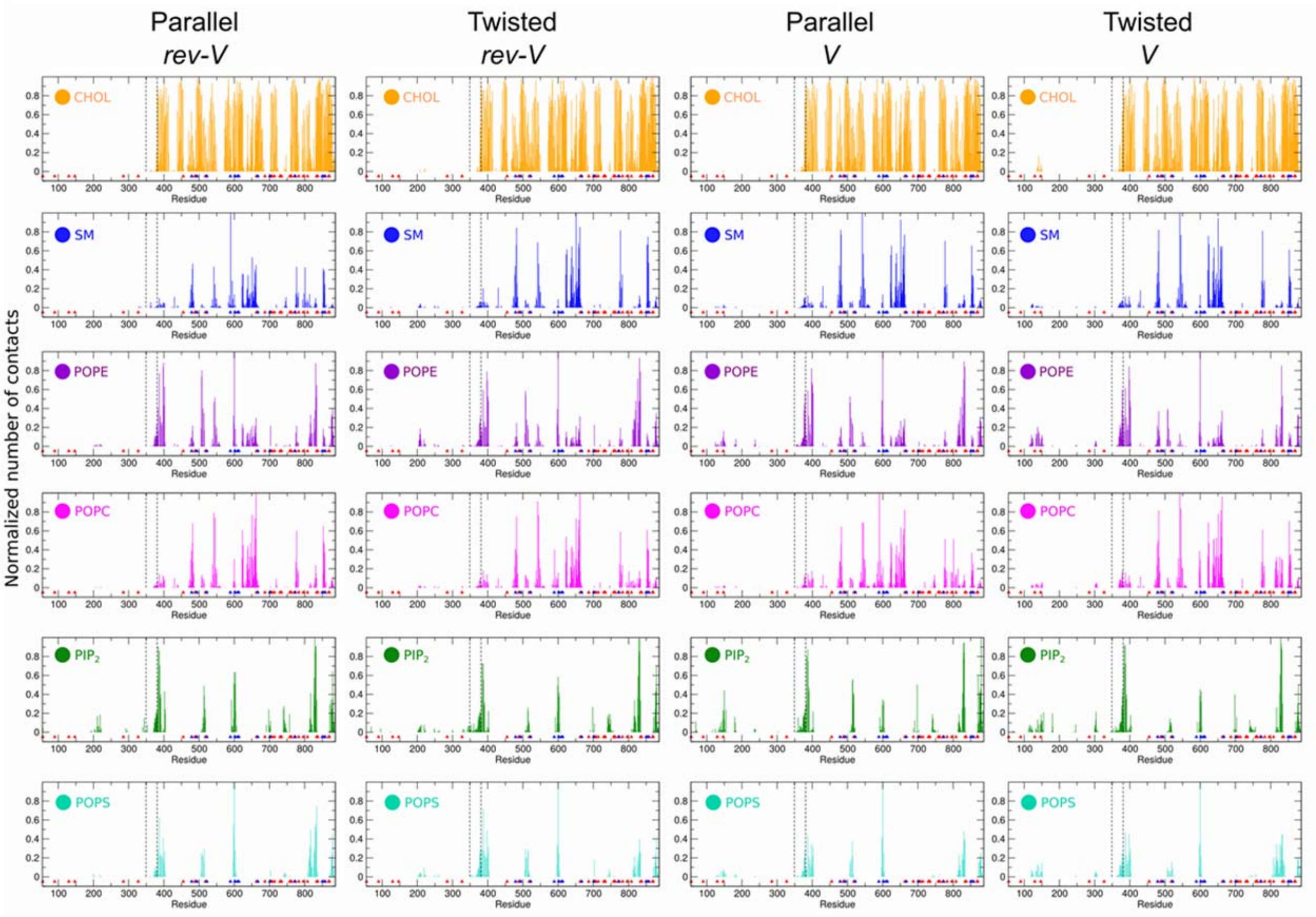

Figure S11. Normalized average number of contacts between AE1 and lipids. Related to Figures 5 and 6. The number of contacts between the protein and the lipids was averaged for each simulation system and merged for the two AE1 chains. The linker domain is shown by dotted lines. Point mutations reported to cause spherocytosis (SPH) or distal renal tubular acidosis (AD-dRTA) are indicated as *red* and *blue* triangles, respectively. Lipid species are: 1-palmitoyl-2-oleyl-phosphtidylcholine (POPC), 1-palmitoyl-2-oleyl-phosphtidylethanolamine (POPE), 1-palmitoyl-2-oleyl-phosphtidylserine (POPS), Phosphatidylinositol 4,5-bisphosphate (PIP_2), sphingomyelin (SM) and cholesterol (CHOL).

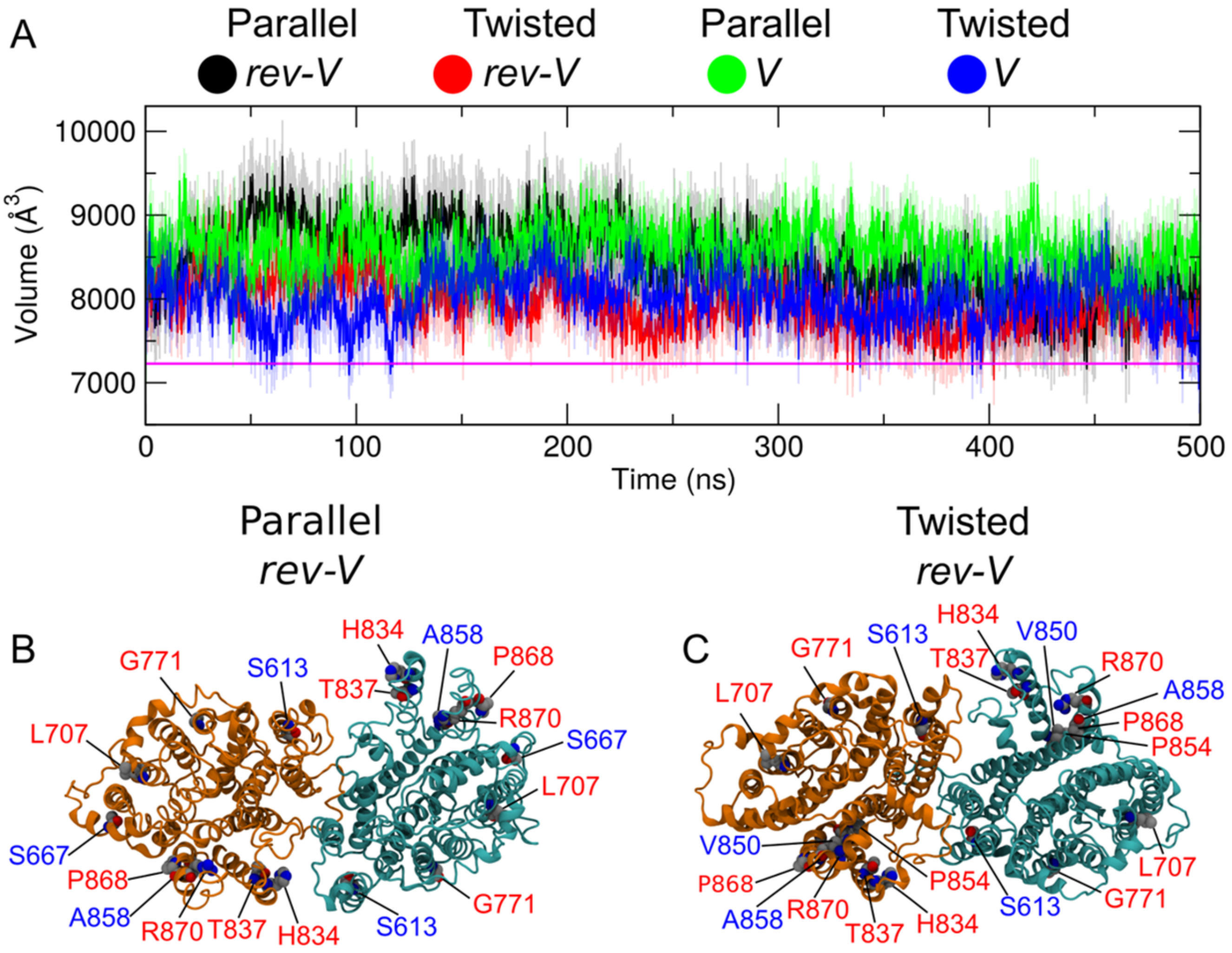

Figure S12. Cavity between the mdAE1 dimer interface and critical residues associated with diseases. Related to Figure 5. (A) Volume of the cavity at the interface of the mdAE1 in all conformers during the atomistic simulations. The calculation has been conducted considering residues 519-546, 569-26 and 785-816 as the boundary of the cavity. The *pink* line is the value calculated, over the same selection of residues, in the mdAE1 crystal structure. The error bars are shown and each timeline represents the average over the three repeat atomistic simulations. (B and C) Residues with normalized number of contacts with cholesterol above 0.5 which have been shown to cause human spherocytosis (SPH) and distal renal tubular acidosis (AD-dRTA) when mutated are shown in *red* and *blue*, respectively.

Table S3. H-bonds between AE1 and lipids in the atomistic simulations. Related to Figure 6.

**Parallel** *rev-V*		**Twisted** *rev-V*		**Parallel** *V*		**Twisted** *V*	
PIP_2 Lys600.B	95.82	PIP_2 Arg387.B	100	PIP_2 Arg832.B	99.31	PIP_2 Arg387.A	99.96
PIP_2 Arg879.A	95.04	PIP_2 Arg388.B	99.85	PIP_2 Arg387.A	98.71	PIP_2 Arg384.A	98.96
PIP_2 Arg603.A	93.82	PIP_2 Arg384.A	99.68	PIP_2 Arg388.A	94.33	PIP_2 Arg384.B	98.42
PIP_2 Lys600.B	95.55	PIP_2 Arg384.B	99.40	PIP_2 Arg827.B	89.46	PIP_2 Arg603.A	96.58
PIP_2 Lys829.A	93.42	PIP_2 Arg871.A	98.95	PIP_2 Arg871.A	84.37	PIP_2 Arg155.A	94.95
PIP_2 Arg384.B	92.55	PIP_2 Arg388.A	98.47	PIP_2 Lys829.A	75.62	PIP_2 Arg832.A	85.89
PIP_2 Lys600.A	91.89	PIP_2 Arg603.A	97.45	PIP_2 Arg514.A	74.71	PIP_2 Lys829.B	85.57
PIP_2 Arg387.B	91.84	PIP_2 Arg387.A	96.32	PIP_2 **Arg870.A**	72.99	PIP_2 Arg387.B	80.68
PIP2 Arg344.B	88.42	PIP_2 Arg344.B	91.50	POPE Arg387.A	72.88	PIP_2 Arg879.B	80.60
PIP_2 Arg832.A	73.93	PIP_2 Arg832.A	78.20	POPE Arg387.B	70.57	PIP_2 Lys600.A	78.37
PIP_2 Arg603.B	67.62	PIP_2 Arg832.B	77.29	CHOL**Gly714.B**	61.92	POPS Arg832.A	75.80
PIP_2 Arg384.B	66.57	PIP_2 Lys817.A	71.05	PIP_2 Lys829.B	61.19	PIP_2 Arg180.A	71.28
CHOL **His834.B**	60.79	PIP_2 Lys600.B	70.50	PIP_2 Lys817.B	60.17	POPC Arg646.A	67.53
PIP_2 Lys817.B	58.17	CHOL **His834.B**	69.41	PIP_2 Arg879.A	56.59	SM Arg782.A	66.97
PIP_2 Arg832.B	58.13	PIP_2 Lys829.A	69.14	PIP_2 Arg832.A	53.44	POPS **Arg870.A**	66.66
POPE Arg387.A	55.28	PIP_2 **Arg602.A**	65.96	POPE Arg832.B	46.79	PIP_2 Lys826.B	62.12
PIP_2 Arg340.B	53.70	PIP_2 Arg514.B	65.74	POPS Lys600.A	43.94	POPE Arg832.B	61.48
PIP_2 Val383.B	50.06	PIP_2 Gly599.B	65.56	PIP_2 **Arg518.A**	43.70	PIP_2 Trp831.A	56.99
SM Lys590.A	49.57	PIP_2 Arg514.A	65.36	POPE Arg827.B	43.21	PIP_2 Arg388.A	56.30
POPS Arg832.B	47.81	PIP_2 Lys829.B	64.02	POPC Lys590.A	42.66	POPS Lys829.A	56.21
POPE Glu658.B	45.14	PIP_2 Lys698.A	59.34	POPS Lys600.B	41.99	POPE Arg387.A	55.61
POPE Arg832.A	42.85	PIP_2 Arg879.B	58.71			PIP_2 Arg827.A	50.32
POPS Lys600.A	42.72	PIP_2 **Arg602.B**	58.67			POPS Lys817.A	48.23
POPS Arg514.B	42.28	POPS Lys600.B	58.56			PIP_2 Trp830.B	48.05
POPS Arg871.B	42.16	PIP_2 Trp831.A	56.61			POPS Lys600.B	46.57
PIP_2 Thr830.B	41.70	PIP_2 Arg827.B	55.19			POPS Glu882.A	43.76
PIP_2 Arg514.A	41.70	PIP_2 Arg345.B	54.34			POPS Lys600.A	39.34
POPS Arg879.B	41.37	POPC Arg782.A	49.61				
POPC Thr853.B	40.81	POPC Arg646.A	48.09				
POPC Arg387.A	40.43	PIP_2 Lys600.A	45.56				
POPE Arg879.A	39.94	PIP_2 Asn880.B	42.69				
SM Arg646.B	39.94						

Numbers are the percentage of occurrence in trajectories. Residues in red and blue cause SPH and AD-dRTA, respectively.
